# Insights into next generation sequencing guided antibody selection strategies

**DOI:** 10.1038/s41598-023-45538-w

**Published:** 2023-10-26

**Authors:** M. Frank Erasmus, Fortunato Ferrara, Sara D’Angelo, Laura Spector, Camila Leal-Lopes, André A. Teixeira, Jesper Sørensen, Suhani Nagpal, Kathryn Perea-Schmittle, Alok Choudhary, William Honnen, David Calianese, Luis Antonio Rodriguez Carnero, Simon Cocklin, Victor Greiff, Abraham Pinter, Andrew R. M. Bradbury

**Affiliations:** 1 Specifica LLC, a Q2 Solutions Company, Santa Fe, USA; 2 New Mexico Consortium, Los Alamos, USA; 3 OpenEye, Cadence Molecular Sciences, Santa Fe, USA; 4 Public Health Research Institute, New Jersey Medical School, Rutgers, The State University of New Jersey, Newark, NJ 07103 USA; 5 University of Oslo, Oslo, Norway

**Keywords:** Bioinformatics, Next-generation sequencing, Antibody therapy, High-throughput screening, Functional clustering, Antibody therapy, Next-generation sequencing, Machine learning, Software

## Abstract

Therapeutic antibody discovery often relies on in-vitro display methods to identify lead candidates. Assessing selected output diversity traditionally involves random colony picking and Sanger sequencing, which has limitations. Next-generation sequencing (NGS) offers a cost-effective solution with increased read depth, allowing a comprehensive understanding of diversity. Our study establishes NGS guidelines for antibody drug discovery, demonstrating its advantages in expanding the number of unique HCDR3 clusters, broadening the number of high affinity antibodies, expanding the total number of antibodies recognizing different epitopes, and improving lead prioritization. Surprisingly, our investigation into the correlation between NGS-derived frequencies of CDRs and affinity revealed a lack of association, although this limitation could be moderately mitigated by leveraging NGS clustering, enrichment and/or relative abundance across different regions to enhance lead prioritization. This study highlights NGS benefits, offering insights, recommendations, and the most effective approach to leverage NGS in therapeutic antibody discovery.

## Introduction

In the therapeutic antibody field, in-vitro display is one of the commonest technologies used to generate antibody leads. Selective pressure (e.g., target concentration) is applied during a selection campaigns, using appropriate antibody libraries, to select antibodies with favorable properties. We recently showed that a carefully crafted antibody library^1^ coupled with sequential in-vitro phage and yeast display^2^ is able to directly identify drug-like leads with favorable developability properties^1,3^, strong binding affinities, and in vitro efficacy by picking and testing random clones. We were able to isolate 31 anti-SARS-CoV-2 antibodies from this library in less than a month, some of which demonstrated potent live virus neutralization, high affinities, and excellent biophysical properties^3^, comparable to the best SARS-CoV-2 antibodies described^4^.

One limitation of random colony screening in selection pipelines is the sampling. While colony picking is effective at identifying therapeutic antibody candidates in a short timeframe^3^, this approach introduces an inherent bias towards the more abundant clones in a selection output. Even high throughput picking campaigns (≥ 10,000 clones) do no more than scratch the surface of the full available diversity in a selection output, particularly when there is clonal dominance. We have found the nonlinear relationship between diversity and sequencing depth is best revealed by next-generation sequencing (NGS), which shows that marginal diversity gains in selection campaigns require substantially more sequencing reads in accordance with a power function. However, questions remain as to the degree this increased diversity is real, or a consequence of PCR amplification and sequencing errors, and whether computational tools, NGS heuristics and machine learning can be used to distinguish functional clones from artifactual ones. Early NGS platforms were limited to short reads allowing analysis of single domains or CDRs, but without full VH/VL pairing, a problem resolved by long-read sequencing platforms such as the PacBio Sequel II system^5^.

Machine learning (ML) has been applied to several applications in antibody discovery and molecular engineering, including prediction of antigen binders from in silico libraries^6,7^, identification of molecular descriptors to predict developability properties^8^, and learning important functional representations of B-cell receptors (BCRs)^9^. ML is usually divided into supervised (e.g. classification, regression) and unsupervised (e.g. clustering) approaches^10^. An example of classification and regression in the context of antibody discovery would be to parse out binders from non-binders or to predict affinity measurements, respectively. In these cases, the aim of the ML algorithm is to minimize the objective (loss) function so that predicted labels or values accurately capture the “ground truth” of experimental data. If no feedback information is available to classify populations (e.g., sequence data without a label defining the associated experimental epitope bin population), unsupervised ML-based clustering can be applied using metrics such a sequence-based similarity to assign antibodies to different clusters.

**Figure 1:**
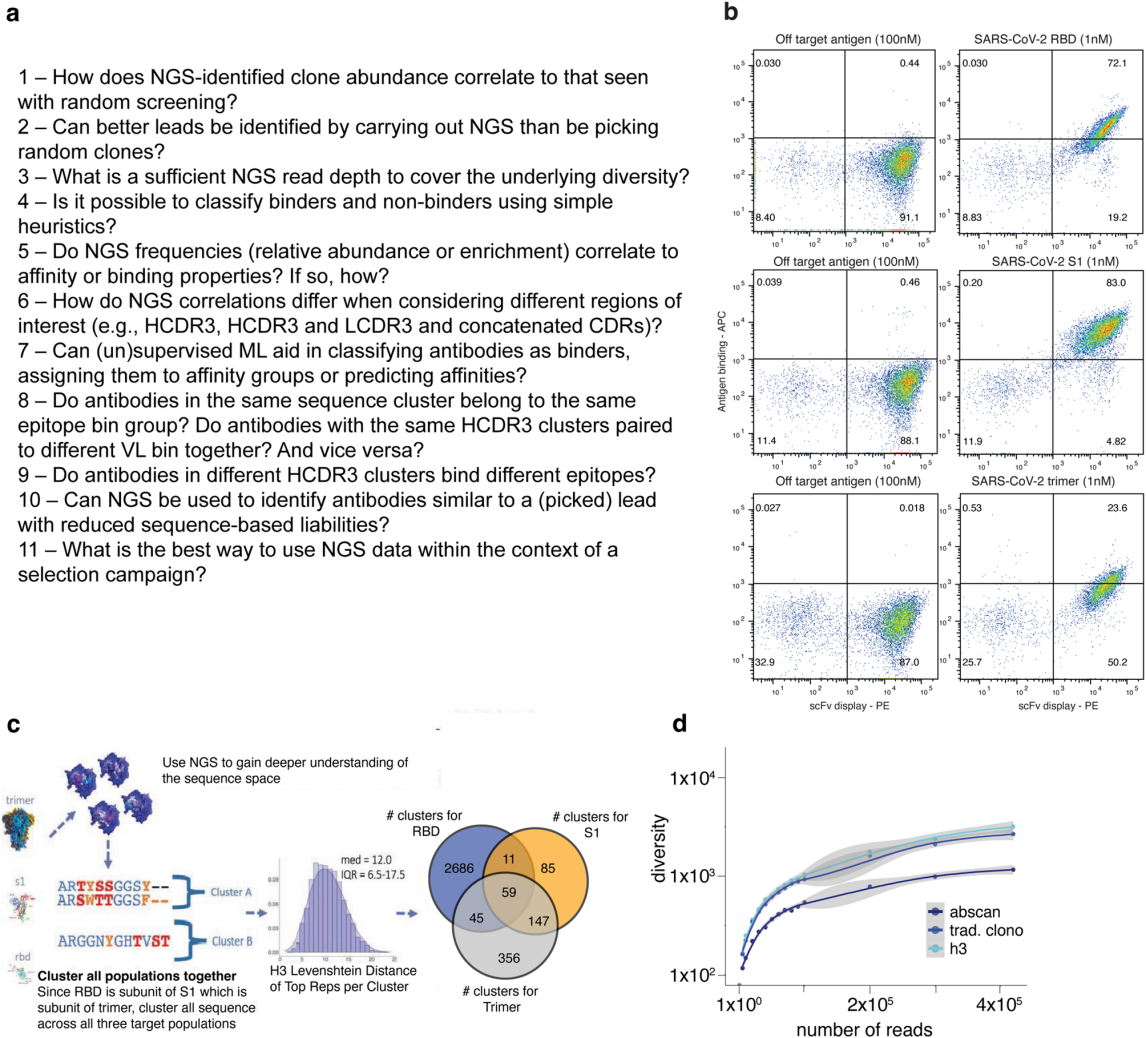
NGS-guided strategy. (**a**) Key questions relevant to any NGS-guided selection campaign (**b**) Final flow plots of yeast displayed selection outputs against RBD, S1 and trimer. (**c**) NGS-guided selection strategy and median differences among different sequences in cluster population. (**d**) Diversity accumulation by read count by given region or clustering method.

In this study, we set out to understand how heuristics and ML methods applied to NGS datasets derived from in vitro discovery campaigns can assist lead prioritization efforts. Using a large SARS-CoV-2 selection campaign as a dataset, our aim was to address the most important questions related to the use of NGS in discovery campaigns (Fig. 1a). Although all these questions were addressed within the context of this SARS-CoV-2 study, the ultimate objective was to identify broad principles generally applicable to all selection campaigns.

## Results

### Selection campaign

We carried out three selection campaigns using our scFv Gen3 semi-synthetic library platform^1^ against the original SARS-CoV-2 spike trimer protein, its monomer S1, and the receptor binding domain (RBD). The campaign was conducted similarly to our previously reported efforts^3^. Biotinylated proteins were used to select antibodies using two rounds of phage scFv panning followed by yeast display^3^. A clear binding population against all three targets was observed (Fig. 1b). Sorting at progressively lower target antigen concentrations (10 and 1 nM) yielded polyclonal populations that were prepared for NGS sequencing using 5′and 3′ in-line NGS barcodes (Supplementary Table 1). A total of six populations (two antigen concentrations for three target antigens) were processed. Additionally, random colonies from the 1 nM sorted populations for each of the three targets were also sequenced by Sanger sequencing.

### NGS-guided strategy

Our NGS-guided strategy was aimed at exploring the functionality of a broad range of antibodies across the entire NGS frequency, enrichment, and sequence space (Fig. 1c). Typical selection campaigns tend to push for high affinity binders, while we set out to test antibodies addressing the questions in Fig. 1a. VH and VL sequences for 200 non-redundant antibodies were synthesized, cloned into mammalian IgG expression vectors, expressed and purified as full length IgG. Of these 200 tested antibodies, 169 (84.5%) bound the RBD, S1 or trimer with affinities < 1 µM, (see “Supplementary Information 3”), mirroring our previously described (74–92%) scFv to IgG conversion rates^1^. The 200 non-redundant antibody sequences were chosen from 57 highly distinct clusters (derived as described below) found at the intersection of all three target populations (41), only in the S1 (1) or RBD (1) populations, or 14 clusters derived from the trimer NGS population, regardless of whether they intersected with S1 or RBD and based on the most abundant representative per cluster.

**Figure 2:**
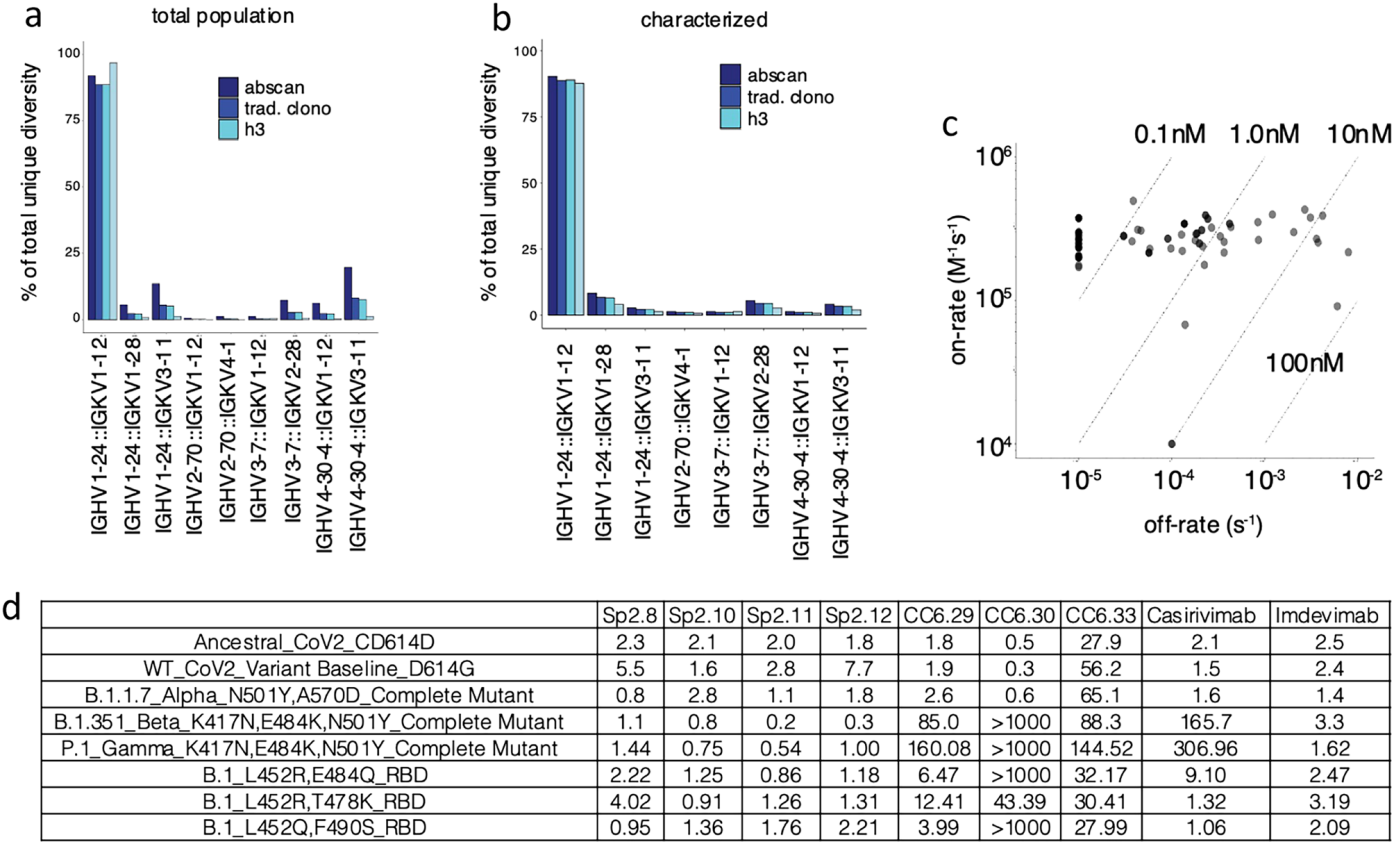
NGS-guided diversity and neutralization. (**a**) Scaffold distribution from selection output condensed on the total unique clonal diversity in the NGS population. (**b**) Scaffold distribution from selection output condensed on the total unique clonal diversity in the characterized population. (**c**) Isoaffinity plot of unique antibodies binding to RBD (all dots are the same color, although overlapping dots appear darker as these are antibodies with similar kinetic profiles). (**d**) IC50’s of selected antibodies (Sp2.x) against different strains, compared to some published and emergency use authorized antibodies.

Thousands of unique sequences across individual or concatenated CDRs were captured by NGS (Supplementary Table 2), but this reported diversity may be artificially inflated by sequencing and PCR artifacts. To investigate this we used three different bioinformatic clustering approaches, which provides a more realistic picture of the underlying diversity. Clustering methods were defined as 100% identity, traditional clonotyping^11^ or unsupervised clustering (descriptions in methods). A distinct diversity plateau is achieved at ~ 4.0 × 10^5^ reads using unsupervised clustering of the HCDR3 (AbScan; see methods), while 100% identity (turquoise) or traditional clonotyping (blue) methods continue to accumulate diversity, suggesting a greater level of artificial diversity in the dataset (Fig. 1d). Although a mixed pool of germline scaffolds was used in the campaign^1^, most of the selected diversity belonged to the IGHV1-24::IGKV1-12 germline, with a modest representation from other VH/VL pairs, highlighting a potential target-driven preference for specific scaffolds^12^ (Fig. 2a). A similar distribution (Fig. 2b) is observed in the 200 characterized antibodies. For those recognizing the RBD, affinities were determined by surface plasmon resonance (Fig. 2c) and, for a subset, neutralization data on different SARS-CoV-2 variants were measured and compared with previously described antibodies (Fig. 2d)^13–15^.

### Correlation of random clone picking and sanger to NGS frequencies

**Figure 3:**
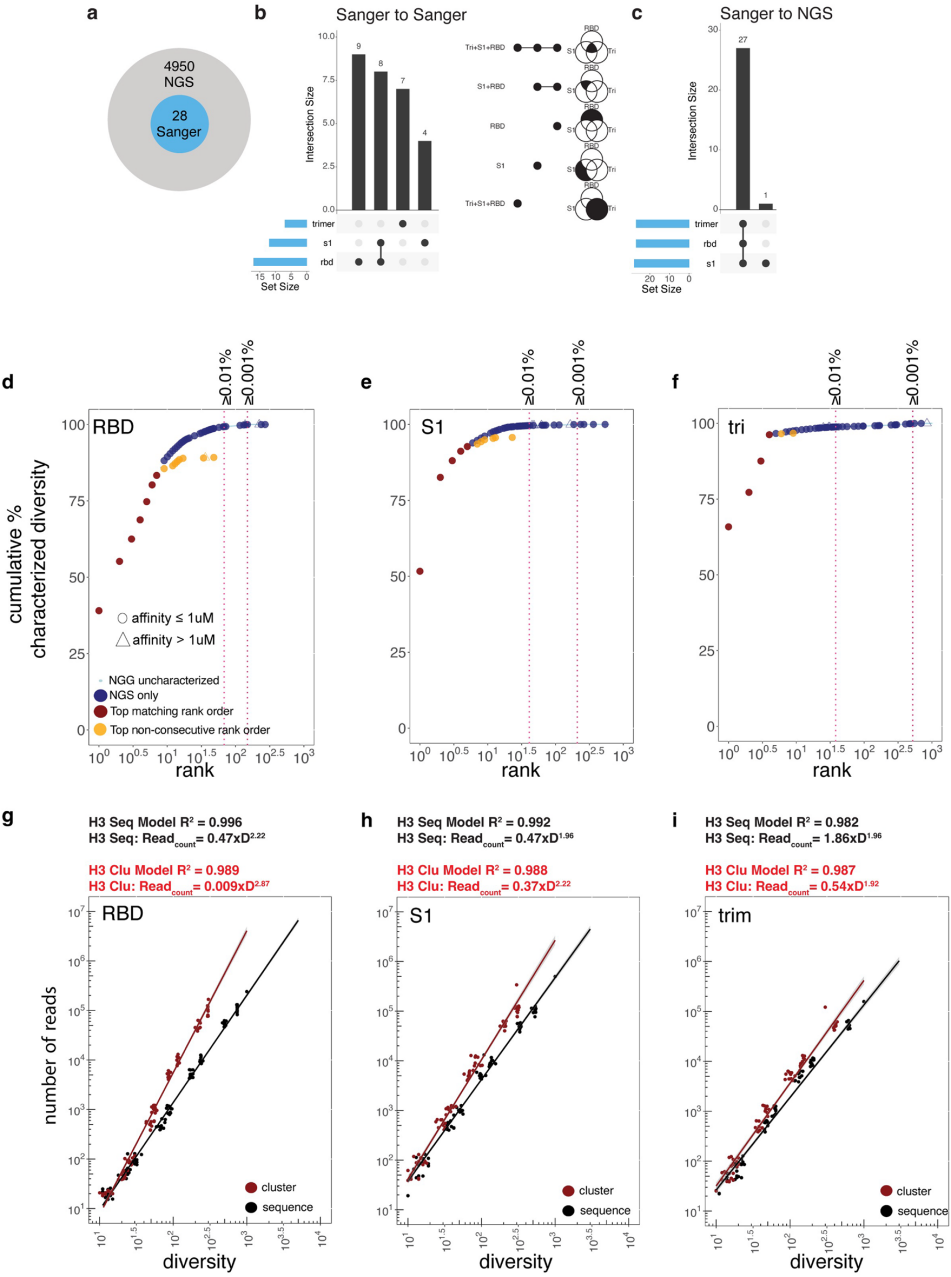
Advantages of deeper read depth. (**a**) Venn diagram showing that all Sanger HCDR3s are identified within the NGS population. (**b**) Extent of overlap using 96-well colony picking across different selection arms using Sanger only or (**c**) within context of same set of clones in context of NGS. (**d**–**f**) Cumulative abundance by the most abundant full-length clone representative in each cluster for populations selected against RBD, S1 and trimer—1 nM concentrations. Colors indicate uncharacterized NGS populations (light blue), characterized clones identified by NGS but not in random colony screen (dark blue), clones identified in NGS and Sanger in consistent rank ordered identified by NGS (dark red) and clones found in random colony screen that begin to deviate from the consecutive rank order identified by NGS (orange). (**g**–**i**) Log_x_-Log_y_ plot of diversity plotted against the total number of reads for the different targets, based random shuffling and sampling per read number and fit to equation above the plot. The color indicates if population is HCDR3 AbScan cluster (dark red) or HCDR3 sequence (black).

As expected, all Sanger clone sequences could be found within the NGS dataset (Fig. 3a), and clones that appeared distinct across the three independent selection arms (trimer, S1 and RBD) by Sanger sequencing (Fig. 3b) were predominantly found across all three populations by NGS (Fig. 3c), due to the increased sampling. To assess how well NGS statistics (e.g., relative abundance) can assist in the ranking of Sanger clones or be used in lieu of Sanger, we plotted the rank order by cumulative frequency gathered by NGS (Fig. 3d–f). The most clonally abundant antibodies from Sanger sequencing were also identified by NGS (dark red in Fig. 2d–f and Supplementary Table 3) with the Sanger clone rank order corresponding directly to NGS abundance, and deviating only at low abundance. The most dominant clone in each cluster is found at 39.0, 51.7 and 65.9% (of the total population) for the RBD, S1 and trimer populations, respectively. The cumulative abundances from the 1 nM sort rounds from RBD, S1, and trimer of the top 10 HCDR3 sequences are 90.5, 97.1 and 97.9%, respectively. All abundant clones (≥ 0.1% full-length abundance) were binders (affinities ≤ 1 µM) to one of the three targets, as were some that were found significantly below the 0.001% threshold (Figs. 3d–f).

To understand how many random clones needed to be picked to capture a particular level of diversity, D, we calculated the diversity (D; x-axis) using a randomly sampled number of reads (f(D); y-axis), then fit the data to a power function of the form:

1 $$f\left( D \right) = C*D^{k}$$

where C and k are constants derived from non-linear fit. The results for both clusters and sequences are shown in Figs. 3g-i for each target. The power factors (k) are quite similar for each of the three targets (1.96–2.22 for HCDR3 sequences and 1.92–2.87 for clusters).

### Correlation of NGS statistics to kinetics

**Figure 4:**
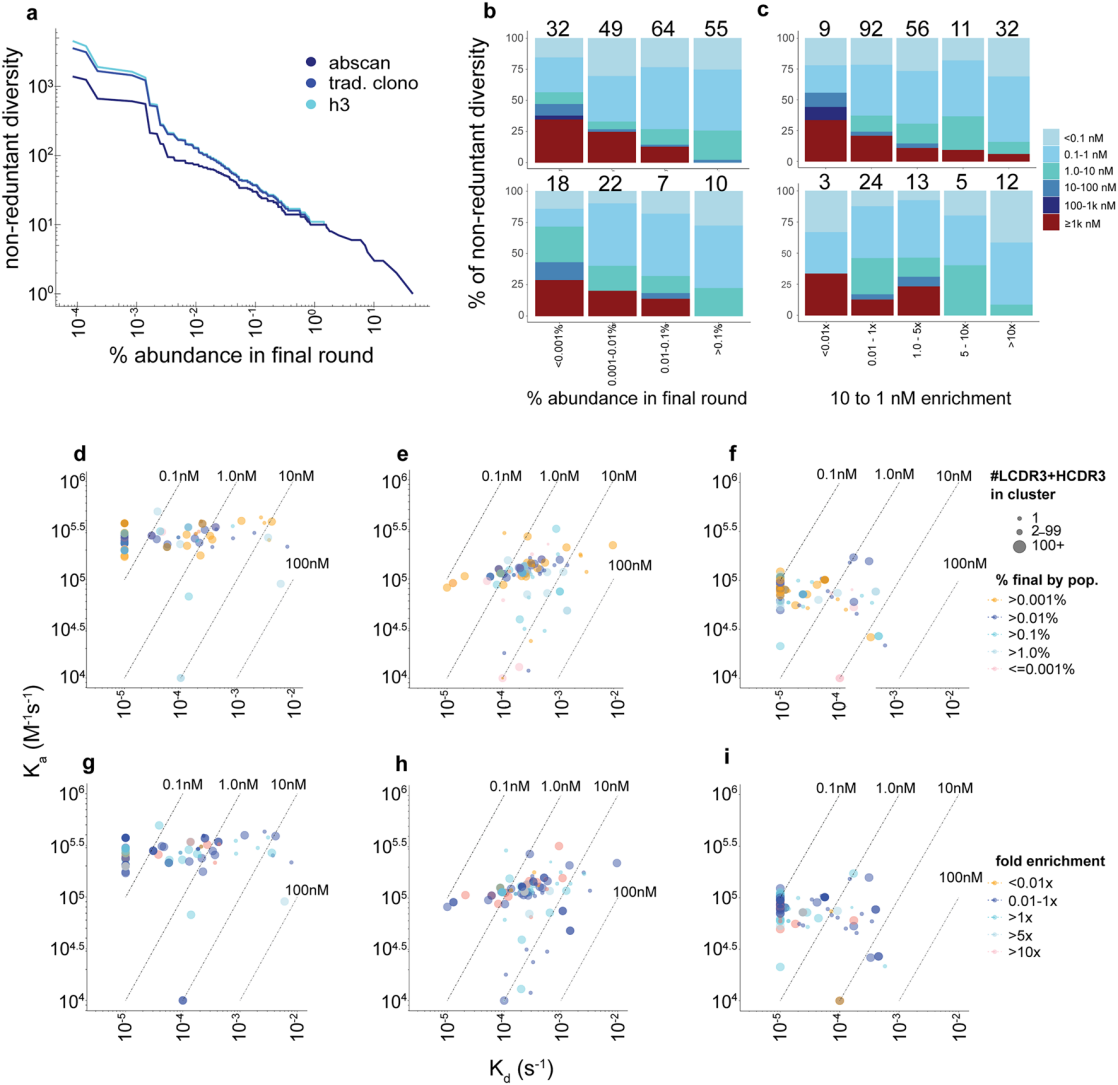
Functionality by relative abundance. (**a**) # of unique HCDR3s, traditional clonotypes or clusters (y-axis) at increasing concatenated CDRs relative abundance cutoffs (x-axis) (**b**, top panel) Relative abundance in 1 nM sort plotted by % of total diversity of all antibodies in the population, with numbers atop plot indicating the total number of antibodies in each bin. (**b**, bottom panel) Relative abundance in 1 nM sort plotted by % of total diversity of most abundant antibodies per AbScan cluster, with numbers atop plot indicating the total number of antibodies in each bin (**c**, top panel) Fold enrichment from 10 to 1 nM sorted populations for all antibodies in population. (**c**, bottom panel) Fold enrichment from 10 to 1 nM sorted populations of the most enriched antibodies found in each AbScan cluster. The numbers above the stacked barplot indicate the number of full-length representatives in each plot bin. d-f) Isoaffinity plots with each point representative of an antibody and the color indicative of the measured relative abundance in NGS across different targets of (**d**) RBD, (**e**) S1 and (**f**) trimer. (**g**–**i**) Isoaffinity plots with each point representative of an antibody and the color indicative of the measured 10 to 1 nM fold enrichment in NGS across different targets of (**g**) RBD, (**h**) S1 and (**i**) trimer. The size of the point in all isoaffinity plots indicates the number of unique HCDR3 + LCDR3 sequences in each cluster.

While diversity provides a comprehensive overview of the number of clones within a given selection output, it does not provide clear information on binding activity. Over 500 distinct (AbScan) antibody clusters were identified at ≥ 0.001% relative abundances (Fig. 4a), and strong binding affinities were measured for many of the synthetized 200 clones (Fig. 2c). Dividing the antibodies into distinct affinity groups using monomeric binding affinities (Supplementary Fig. 1a) revealed that 64% of the antibodies selected from the RBD or S1 populations exhibited sub-nanomolar affinities (≤ 1 nM), and ≥ 75% below 10 nM. 19% of the antibodies exhibited affinities ≥ 1 µM, which we classify as non-binders.

Using a stacked bar plot, we plotted affinity ranges for NGS relative abundance (Fig. 4b, top panel) and fold enrichment (Fig. 4c, top panel) distributions, respectively. The percentage of binders was directly proportional to relative NGS abundance at the 1 nM antigen sort concentration (Fig. 4b, top panel) as well as across increasing enrichment from 10 to 1 nM (Fig. 4c, top panel), with many, and often most, binders in all abundance and enrichment groups exhibiting sub-nanomolar affinities. The numbers of non-functional (≥ 1 µM) binders was improved across the different relative frequency and enrichment bins by selecting the top representative per cluster (Fig. 4b–c, bottom panel). Only at very low abundance (< 0.001%) or fold enrichment (< 0.01x) were substantial non-binding populations (27–30%) found. Nonetheless, antibodies with sub-nanomolar affinities were also found in these depleted or low frequency populations. The % binders by abundance and fold-enrichment is impacted by the target (Supplementary Figs. 1b–d–2a–c).

The isoaffinity plots for relative abundance (Fig. 4d–f) or enrichment (Fig. 4g–i), reveal that binned frequencies across concatenated CDRs are somewhat randomly distributed, with no clear trends across the kinetic profile to suggest correlation of affinity to NGS metrics. Within the RBD population, we identified 30 antibodies with ≤ 100 pM affinities. The number of ≤ 100 pM binders against RBD were 3/30 (10%) below 0.001% abundance, 11/30 (37%) below 0.01%, 17/30 (57%) below 0.1%, 27/30 (90%) below < 1.0%, and 3/30 (10%) above 1.0%, reflecting the apparent lack of correlation between abundance and affinity. The total number of clones depleted in the 1 nM population relative to the 10 nM population was 11/30 (37%), with one binding clone 1/30 (3.3%) depleted < 0.01x, while the number enriched was 19/30 (63%), with 5/30 (17%) clones enriched more than tenfold.

### Using unsupervised machine learning to efficiently explore sequence diversity

**Figure 5:**
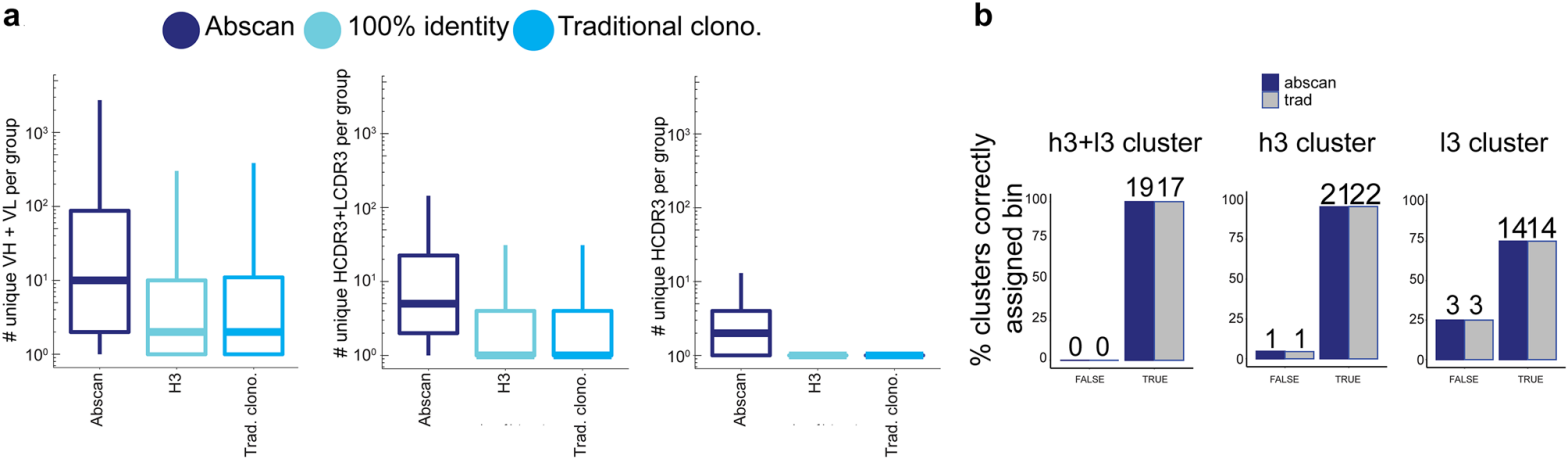
AbXtract clustering relevance to experimental binning data. (**a**) The number of non-redundant full-length (left), HCDR3 + LCDR3 (middle), and HCDR3 (right) sequences by cluster type. (**b**) The percent of correctly assigned experimental bins with at least two non-redundant sequences (VH + VL) per cluster, using different criteria: 100% identity for HCDR3 + LCDR3 (left), HCDR3 (middle), or LCDR3 (right).

Sequence-based CDR clustering reduces the complexity of the NGS output, minimizing redundancy and maximizing the exploration of paratopic diversity. We used AbScan, an unsupervised machine learning algorithm based on OPTICS (see methods), to cluster antibody CDRs. The AbScan clustering typically results in a larger number of non-redundant VH + VL, concatenated HCDR3 + LCDR3 and HCDR3 sequences per AbScan cluster relative to other traditional clonotyping (see methods) approaches (Fig. 5a).

An essential concept behind clustering is that antibodies belonging to the same cluster (HCDR3 and/or LCDR3) bind the same epitope. As shown in Fig. 5b, we quantified the number of clusters using either AbScan (dark blue) (Supplementary Tables 6–7), or traditional clonotyping methods (gray). All antibodies using HCDR3 + LCDR3 clusters showed similar experimental bin profiles—i.e. they bound the same epitope within the constraints of SPR binning (Fig. 5b, left). Antibodies belonging to very distinct HCDR3 clusters exhibit broad binding kinetics (Supplementary Fig. 3a–c). In this dataset, 22/23 (96%) of the traditional clonotypes and 21/22 (95%) of the AbScan clusters bind to the same epitope as defined by SPR binning (Fig. 5b, middle). This contrasts with the LCDR3 population where 14/17 (82%) and 14/17 (82%) of the LCDR3 AbScan clusters and traditional clonotypes bind to the same epitope, respectively (Fig. 5b, right). In conclusion, antibodies in the same HCDR3 cluster almost always bin together (Fig. 5b, middle, Supplementary Fig. 6), antibodies in different HCDR3 clusters may or may not bin separately (Supplementary Figs. 3d–f–4–5), while antibodies in the same LCDR3 cluster frequently bin together, but less consistently than HCDR3 clusters (Fig. 5b, right). Furthermore, with NGS we were able to expand the paratope coverage and identify a total of five SPR bins comprising four RBD/S1 bins (Supplementary Fig. 7) and one additional specific for the trimer (Supplementary Fig. 8a–c), compared to only two bins identified by picking clones.

One additional advantage of clustering sequences is the ability to identify additional antibodies within a cluster with reduced numbers of sequence-based liabilities (see Table 1 from Teixeira et al.^1^), relative to the most abundant clone in cluster (Supplementary Fig. 9a–b). We identified seven clusters containing additional sequences with reduced numbers of sequence liabilities (Supplementary Fig. 9b; blue) relative to the most abundant sequence within the cluster (Supplementary Fig. 9b; dark red), some of which also exhibited improved, similar or worse affinities.

### Correlation of NGS metrics to kinetics

**Figure 6:**
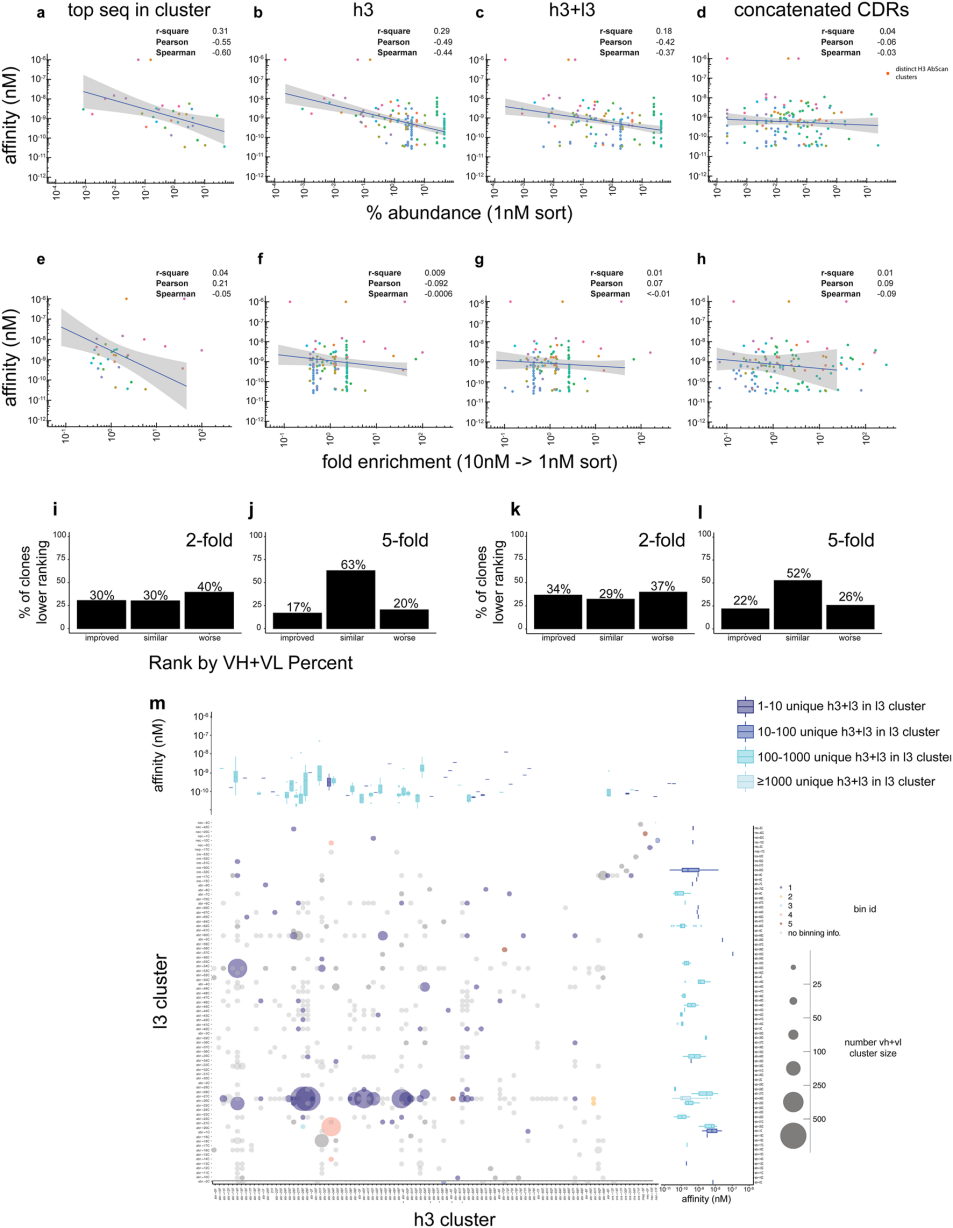
Correlation of NGS to Affinity. Scatterplot of relative abundance versus affinity for (**a**) top representative in given cluster, (**b**) all unique HCDR3, (**c**) all unique concatenated HCDR3 + LCDR3, and (**d**) concatenated CDRs. Scatterplot of fold enrichment (10 nM to 1 nM) versus affinity for (**e**) top representative in given cluster, (**f**) all unique HCDR3, (**g**) all unique concatenated HCDR3 + LCDR3, and (**h**) concatenated CDRs. The number of additional clones in each cluster with lower relative abundance for the full-length sequence with improved, similar or worse affinities using (**i**) twofold and (**j**) fivefold threshold. The number of additional clones in each cluster with lower fold enrichment for the full-length sequence with improved, similar or worse affinities using (**k**) twofold and (**l**) fivefold threshold. (**m**) Paired sequence profile of the HCDR3 clusters (x-axis) versus the LCDR3 clusters (y-axis). All selected antibodies are color-coded according to their corresponding bin group, except gray which are the non-selected NGS clones. The size of dot is reflective of number of additional full-length sequences within a given cluster. Two additional boxplots show the affinity range across each respective cluster with top boxplot showing same HCDR3 clusters paired with different LCDR3 clusters. Right boxplot shows same LCDR3 cluster paired to many different HCDR3 clusters. Boxplot are color coded to show the number of unique HCDR3 and LCDR3 in the cluster population.

One promise of NGS has been the idea that simple NGS heuristics (relative abundance or enrichment) can be used to rank antibody populations or clones for binding activity. We plotted relative abundance and enrichment versus affinity for HCDR3, HCDR3 + LCDR3, concatenated CDRs, and the most abundant VH + VL sequence in each AbScan cluster. Figure 6a–h show that the abundance of the top representative in each AbScan cluster population (Fig. 6a) gave the best correlation (r-square = 0.31, Pearson = − 0.55, Spearman = − 0.60) to affinity, off-rate and on-rate (Supplementary Figs. 10−12), with a dramatic reduction in correlation coefficients to affinity as more CDR regions are included when calculating relative abundance (Fig. 6b–d; Supplementary Figs. 10–12). The 10 nM to 1 nM enrichment ratio was not correlated across any region of interest (ROI; e.g., HCDR3, HCDR3 + LCDR3, concatenated CDRs) (Fig. 6e–h). No antibody ROI abundance or enrichment parameters could be well correlated to kinetics of antibodies selected against the trimer (Supplementary Figs. 10q–x, 11q–x and 12q–x), reflecting the important role avidity and epitope play in multimeric targets.

While selecting the top representatives of each cluster provides reasonable correlations for affinity, this was not the case for less abundant clones belonging to the same AbScan cluster: 70% of clones with lower NGS abundance relative to the top clone in the cluster have similar (K_D_ within twofold) or worse affinities, while 30% exhibited affinities at least two-fold better than the top clone in the cluster (Fig. 6i), and 17% had affinities at least five-fold better than the top clone in the cluster (Fig. 6j). Applying a similar analysis to the 10 nM to 1 nM enrichment values, 66% of those clones which were less enriched than the top clone in a cluster had similar or twofold worse affinities, while 34% exhibited at least two-fold improved affinities (Fig. 6k), and 22% had five-fold improved affinities (Fig. 6l). Antibodies within HCDR3 clusters can have broad affinity ranges when paired with different LCDR3 clusters (Fig. 6m).

### Discriminating and ranking antibodies with XGBoost

**Figure 7:**
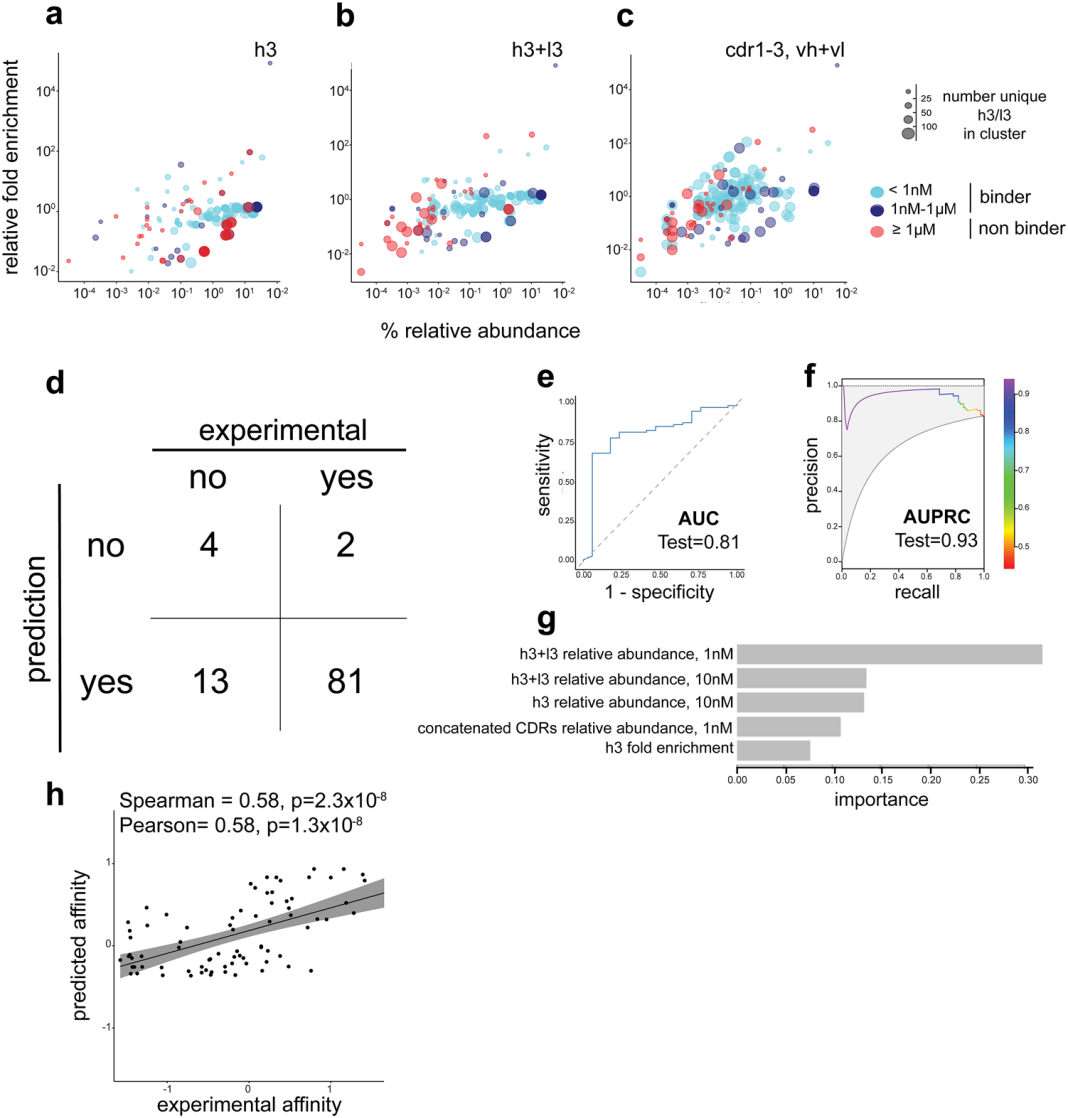
ML Classification, Regression & Key Descriptors. Relative abundance (x-axis) is plotted against the 10 nM to 1 nM fold enrichment using values obtained from regions of interest of the (**a**) HCDR3, (**b**) HCDR + LCDR3 and (**c**) concatenated CDRs. (**d**) Confusion matrix from the XGBoost binary classification using no sequence information (NGS stats only) prediction of binders versus non-binders. Performance is shown via (**e**) area under the ROC curve (AUC = 0.921) and (**f**) area under the precision/recall curve (AUPRC = 0.966). (**g**) Top 5 non-sequence descriptors contributing to the model. (**h**) Regression analysis from XGBoost predicted outputs with experimental data using only NGS population features (e.g., relative abundance across different ROIs), and no sequence-based features (e.g., biophysical properties or one-hot encoding). Performance is evaluated using Pearson or Spearman (top of plot).

While the data above suggests selecting the most abundant full-length sequence within each different cluster performs well at correlating affinity to a single feature of NGS relative abundance (Fig. 6a), this correlation to affinity is ablated using the relative abundance of concatenated CDRs (Fig. 6d). When we combine features from different regions of interest, e.g., plotting the fold enrichment against relative abundance across different ROIs, we start to see differential patterns discriminating the binders (< 1 µM) from the non-binders (≥ 1 µM) (Fig. 7a–c). We hypothesized that the use of a well performing shallow learning method (XGBoost), with decision tree capabilities, with a broad set of features (Supplementary Tables 8–9) as input, would not only be able to discriminate binders from non-binders but also perform well by showing stronger correlations of predicted affinities to experimental affinities. This would allow one to use this predicted affinity to rank order full-length antibodies across the entire population (including within clusters) as opposed to just selecting the most abundant clone across distinct clusters (Fig. 7a).

The XGBoost model trained on an expanded feature set (Supplementary Tables 8–9) performed moderately well for both binary classficiation (discrimination of binders from non-binders) and regression (correlation of predicted affinities to measured affinities) setting. Owing to the limited size of the dataset, we implemented an equal train/test partition (Supplementary Fig. 13a) for conducting bootstrap resampling (Supplementary Fig. 13b–c), which facilitated a more accurate evaluation of the model variability within the training set and enabled hyperparameter optimization (Supplementary Table 10). The confusion matrix (Fig. 7d) provides an overview of the classification of binders and non-binders in the final test set of non-redundant antibodies, achieving precision and recall values of 0.86 and 0.98, respectively. The model exhibited AUC (area under the curve) of 0.81 (Fig. 7e). Due to the highly unbalanced nature of the dataset, we used AUPRC (area under precision recall curve) which exhibited a value of 0.93 (Fig. 7f). From the model, we extracted the top five most important predictors (Fig. 7g), with the relative abundance of paired CDR3 (HCDR3 + LCDR3) at 1 nM sort concentrations ranked as the most important feature for binder versus non-binder prediction. The XGBoost model under regression showed moderate correlations of predicted affinities to experimental affinities when using the expanded feature set as input with Pearson and Spearman coefficients of 0.58 (*p* = 2.3 × 10^–6^) and 0.58 (*p* = 1.3 × 10^–6^), respectively (Fig. 7h).

## Discussion

The traditional approach used to generate antibody leads in an in vitro discovery campaign has been to carry out selections, and pick random clones for testing. Although it is generally assumed that NGS expands the number of possible leads, few in depth studies have explored this. In Fig. 1a, we have listed questions we wished to tackle within the context of this study, and addressed below.

1. **How does NGS-identified clone abundance correlate to that seen with random screening?** Since we have an upper limit of diversity based on the total number of sorted events in FACS (10,000 events per concentration per target in this study), any diversity exceeding this represents errors introduced during sorting, (PCR) sample preparation and/or sequencing. As selection outputs are generally distributed according to a power law (a few clones are highly represented in the population, and most are extremely rare), the likelihood of obtaining 10,000 unique clones by clone picking is beyond most capabilities. If NGS-derived frequency is taken as the ground truth, statistically the Sanger clones from a 96-well plate should appear at the upper frequency thresholds in the NGS population. This is what we find in Fig. 3d–f and Supplementary Table 3, where there is 100% correspondence between the most abundant 4–9 of the non-redundant Sanger HCDR3 sequences and the top NGS HCDR3s. Only at the lower (NGS derived) frequency values do we observe NGS clones not sampled in the 96-well format, indicating that NGS clone abundance correlates relatively well with random screening.
2. **Can better leads be identified by NGS than picking random colonies?** In a previous work^16^, antibodies selected by random picking exhibited very high affinities, but they do not compare to those found here. When NGS was incorporated into the discovery campaign we were able to isolate over 30 antibodies with affinities below 100 pM (Fig. 2c). Furthermore, while all the picked antibodies^16^ appeared to recognize the same epitope, there was greater epitope diversity among those identified by NGS, demonstrating the value of NGS to expand antibody properties in discovery campaigns, in terms of both affinity and epitope diversity.
3. **What is a sufficient read depth to cover underlying diversity?** Apart from fully exploring diversity for screening purposes, a minimum diversity is often required for bioinformatic purposes, such as machine learning. Using the data outlined in Fig. 3g–i, we obtained a rough estimate of the underlying number of reads required to obtain a particular level of desired antibody diversity. For example, if we wanted 1,000 unique HCDR3s upon repeat selection against these three targets at 10 nM and 1 nM, 215 K to 402 K sequence reads per target population would be required. If the total number of desired clones (or desired clusters) remains small, random colony picking is usually more than sufficient, providing dominance is taken into account (where NGS can provide a clearer picture). Further, these campaigns show that a linear increase in the number of unique sequences or clusters desired, requires the number of picked clones to be approximately squared (e.g. if 10 unique clones/clusters are found by picking 50 clones, ~ 2500 clones (50^2^) need to be picked to obtain 100 unique clones/clusters). It goes without saying that NGS cannot conjure up functional diversity that does not exist in a selection output. In Fig. 1d the number of identified AbScan identified clusters comes to a plateau, reflecting the maximum number of potential binders.
4. **Is it possible to classify binders and non-binders using simple heuristics?** Essential to any NGS selection campaign is understanding how rare a given clone can be before the bulk of sequences exhibit declines in functionality. We were rather surprised to find subnanomolar binders at very low NGS frequencies (< 0.001%) (Figs. 3d–f and 4b). While the data suggest relative abundance and fold enrichment could be used to discriminate antibodies into binders and non-binders, the level of discrimination was highly dependent on the target (Supplementary Figs. 1b–d and 2a–c). For example, most trimer-selected clones with ≤ 0.01% relative frequency were non-binders, whereas most clones across all frequency distributions were binders for the RBD and S1. This may be due to the integrity and folding of the larger trimer complex, or its trimeric nature, although this will need to be further explored. Notwithstanding different results obtained with different targets, empirically we set an abundance threshold of 0.005% of the concatenated CDRs to generally distinguish binders from non-binders in our selection campaigns, understanding that high affinity antibodies can still be found at lower abundances, recommending deeper NGS diversity exploration when more antibodies are required.
5. **Do NGS frequencies (relative abundance or enrichment) correlate to affinity or binding properties? And if so, how?** One interesting finding from our study was the weak to moderate correlation between affinity and abundance and/or enrichment for most of the regions of interest when antibodies were assessed following a single 10 to 1 nM selective step (Fig. 6b–d, 6e–h). This has been previously reported^17–20^, with good correlations between enrichment ratios and *binding activity*, but not between enrichment ratios and *antibody affinities*. While it is possible that the high affinities (< 1 nM) of many of the binding antibodies in our RBD population would prevent a selective advantage under our experimental conditions, we also found that when different affinity bands (< 100 pM; 100–1000 pM; 1–10 nM; 10–100 nM and > 100 nM—Supplementary Fig. 14) were examined individually, there was no enrichment of antibodies in the 1–10 nM affinity range, which would have been expected. Selection from in vitro libraries is clearly highly effective, given that high affinity binders can be isolated after a number of selection rounds from naïve libraries with > 10^10^ diversity. However, enrichment over a single selection step, particularly relatively late in the selection process (going from 10 to 1 nM in yeast display) appears insufficient to demonstrate statistical significance. It is possible significant enrichment more correlated to affinity may occur earlier in the selection process, or over a number of rounds. These are possibilities we are further investigating separately.
6. **How do NGS correlations differ when considering different regions of interest (e.g. HCDR3, HCDR3 and LCDR3, concatenated CDRs)?** One question that leads to uncertainty is which region of interest correlates best to affinities. Naturally, the full-length sequence of antibodies presents the most straightforward path from NGS-to-clone, yet when we use full-length sequence metrics of relative abundance (Fig. 6d) and fold enrichment (Fig. 6h) we show the worst correlations to affinities. Since decent correlations were obtained by selecting the top representative between clusters (Fig. 6a), this approach presents a simple strategy to select antibodies in the population. It is rather peculiar that the top most relative abundance does not provide the most straightforward path to clone selection. We hypothesize that greater selective pressure (lowering antigen concentration) with the corresponding reduction in diversity results in a heavily skewed population with few antibodies dominating the output. This may result in an elevated number of sequences per cluster that are not derived from the “true” sorted population but PCR or sequencing artifacts. By selecting the top clone per cluster, we are better able to not only segregate binders based on paratope diversity, but also, reduce the number of aberrant sequences that may or may not be impacted by selective pressures and round-to-round enrichment during yeast sorting. If additional leads with improved affinities are desired, particularly within a particular cluster, additional antibodies can be synthesized and tested, with the expectation that (at least in this dataset) ≤ 30% (17%) of additional clones will exhibit twofold (fivefold) improved affinities (Fig. 6i–j), but the majority will not.
7. **Can supervised ML aid in classifying antibodies as binders, assigning them to affinity groups or predicting affinities?** When using ML, selection outputs can be analyzed without any prior binding information, or within a context in which initial binding information has been gathered across select sample populations. To be successful without specific binding information, generalizable statistics, such as relative abundance of given regions of interest, or enrichment across multiple concentration rounds, should provide insights into ranking. We acknowledge that the present study is limited by the total number of observations (N = 200) and interrelated targets. Nonetheless, these data reveal a decent model performance can still be obtained under both the binary classification and regression settings. Using features restricted to the NGS stats and framework regions (Supplementary Tables 8–9), we were able to classify antibodies as binders or non-binders (AUC ≥ 0.81; AUPRC ≥ 0.93—Fig. 7e–f) and improve correlations to affinities (Pearson = 0.58; Spearman = 0.58—Fig. 7h). However, since these are interrelated targets, the generalizability of this model to different antigen classes remains to be determined.
8. **Do antibodies in the same sequence cluster bin together? Do antibodies with the same HCDR3 clusters paired to different VL bin together? And vice versa?** Traditional clonotyping uses a % identity threshold (80–100%), the same HCDR3 length and V/J call^11^. This is less practical in selection analysis, as there may be too little/much collapsing of edit distance if threshold is set too low/high, respectively. We developed AbScan within our AbXtract module to take an unbiased clustering approach that relies on amino acid chemical properties (Supplementary Table 5) over identity as well as the population densities of the sequenced populations. This provides AbScan with several advantages over traditional clonotyping strategies: (1) no hard edit distance cutoffs are required (unbiased), (2) population densities are utilized, and (3) physicochemical reduction reduces complexity. This allows the underlying data structure and diversity distribution to drive the most optimal cluster cutoffs with reduced bias. In all these instances we observed that antibodies belonging to the same cluster almost always (95%) engaged the same epitope, including some highly dissimilar HCDR3 sequences (58–60% identity) (Supplementary Figs. 4–5). A big weakness of this study was the fact that the binning space was severely limited relative to the total number of clusters identified. Nevertheless, LCDR3s clustered similarly behaved differently, with only 82% of antibodies in an LCDR3 cluster binning together, indicating HCDR3 clusters are far better at predicting similar epitope binning. Although this reflects the greater importance of the HCDR3, compared to the LCDR3, in target binding^21^, additional targets, particularly with many distinct epitopes, will be needed to validate this concept more thoroughly.
9. **Do antibodies in different HCDR3 clusters bind different epitopes?** One outcome from this study, was the observation that antibodies in many distinct HCDR3 clusters show similar binning profiles as determined by experimental SPR, confirming that antibodies in different clusters can bind similar epitopes. While such binning is rather crude in its ability to identify subtleties in epitope space, this mirrors data obtained from X-ray crystallography of complexes between antibodies from Covid patients and the RBD, in which antibodies with quite different HCDR3 sequences, but similar germlines, can bind the same epitope almost identically^22^. We were able to identify five different epitope bins by SPR, 4 against the RBD (Supplementary Fig. 7) and one additional against the trimer (Supplementary Fig. 8), in the NGS identified antibody population, some of which are known to be neutralizing^16^. In this study antibodies binning differently came from different HCDR3 clusters in all but one case, allowing us to conclude that while antibodies from different HCDR3 clusters may bind the same epitope, antibodies binding different epitopes are generally derived from different HCDR3 clusters. The one exception recalls previous data indicating that the HCDR3 is necessary, but insufficient for specific binding^23^. The antibody binding modes for any SPR-defined bin can vary significantly and are best revealed using detailed epitope analysis techniques, such as alanine scanning, deep mutational scanning^24–26^, or deep mutational learning^27^. The literature describing different SARS-CoV-2 spike epitopes is complex^4,28,29^, with six main antigenic sites and 16 epitopes, 50% of which are found in the RBD. Whether the five bins we identify by SPR correspond to any of these 16 epitopes awaits further study.
10. **Can NGS be used to identify antibodies similar to a lead, with reduced sequence liabilities?** Working under the assumption that antibodies in the same cluster exhibit similar specificities (epitope) and are similarly functional (improved or minimal deviation from top representative) then NGS offers the means to select additional sequences that can potentially have reduced number of sequence-based liabilities. In Supplementary Fig. 9a, we revealed the extent to which non-redundant sequences with reduced numbers of sequence liabilities can be selected if using NGS. To showcase this point, we plotted antibodies with fewer liabilities relative to most abundant sequence in a given cluster by their RBD binding affinities, Supplementary Fig. 9b, revealing that additional sequences can be selected that not only have reduced sequence-based liabilities but also similar or improved affinities.
  Finally, and perhaps most importantly, is:
11. **What is the best way to use NGS data within the context of a selection campaign?** We find NGS to be particularly useful in assigning selected antibodies into HCDR3 clusters. This provides additional paratopic and epitopic diversity over and above the more restricted diversity found by random picking, particularly when there is strong clonal dominance. In general, applying a threshold abundance of 0.005% using the concatenated CDRs as a basic heuristic allows good discrimination between binders and non-binders, although many antibodies with lower abundances are often binders if greater diversity is required. Within any cluster our top choice for testing is the most abundant *full-length* sequence, which most often binds the target. In the present study, we noted that populations that were heavily skewed (clonally dominant; Fig. 3f) resulted in a significantly poorer binding performance for less abundant clones (e.g., trimer; Supplementary Fig. 1d), though we are currently exploring how important this skewedness is to binding prediction from one antigen population. Lastly, in campaigns where selective pressure is applied with decreasing antigen concentrations, training data on NGS statistics across multiple regions of interest (HCDR3, HCDR3 + LCDR3, concatenated CDRs) collectively combined with shallow learning model like XGBoost can assist in lead ranking on affinity and classify antibodies from NGS as binders or non-binders.

Although we acknowledge this study was limited to the selection of antibodies against SARS-CoV-2 as well as the lack of independent replicates from each of the populations, we believe the NGS analyses described will provide a valuable resource in other campaigns, whether in vitro or in vivo.

## Materials and methods

### Antibodies selection

scFv were selected against SARS-CoV-2-RBD (SPD-C52H3, ACROBiosystem), SARS-CoV-2-S1 (SIN-C82E8, ACROBiosystem) and SARS-CoV-2-Trimer (SPN-C82E9, ACROBiosystem), using a strategy that combines phage and yeast display as decribed in Ferrara et al.^16^ Details on the semi-synthetic library that we used in the study has been detailed previously^1^. Briefly, we embedded CDRs derived from natural deep sequenced pools of human donors into a diverse panel of developable clinical antibody scaffolds. Because the HCDR3 diversity is well above the capacity of oligonucleotide array-based synthesis, these were generated directly from CD19 + B-cells from donor LeukoPaks. All remaining CDRs were derived from replicated natural diversity post-analysis of NGS sequencing of a previously published library comprised of 40 donors^30^.

### Pseudovirus neutralization assay

The neutralization assays with pseudoviruses expressing the different variants of SARS-CoV-2 were peformed as previously described^16^.

### NGS preparation and barcoding

ScFvs from miniprepped samples were amplified with primers annealing to the 5′ and 3′ of VL and VH, respectively (the scFv format is VL-linker-VH). Primers contain in-line barcodes enabling demultiplexing of populations according to Supplementary Table 1. After amplification, samples were purified using Zymo HT at the correct size associated with the scFv nucleotide sequence, including flanking regions (~ 850 bp). Purified products were outsourced 2 separate PacBio Sequel II (Brigham Young University) sequencing performed on Pool A and B consisting of over 2.1 M reads. Consensus building was conducted at the sequencing facility and FASTQ files were generated. Similarly, individual colonies were picked from round 3 for each of the selections carried out against the individual targets (equivalent of RBD-3, S1-3 and trimer-3 in NGS**)**, miniprepped and processed for Sanger sequencing at Genewiz, Inc.

### NGS sequence processing and annotation

We built a streamlined set of bioinformatics workflows in the cloud called AbXtract™ (eyesopen.com/orion/abxtract), which are end-to-end solutions for antibody discovery using in vitro display. The workflows were built on the Orion® platform (eyesopen.com/orion) in collaboration with OpenEye, Cadence Molecular Sciences. The processing is done as a series of steps from demultiplexing, FASTQ filtering, annotation, clustering, enrichment, and liability quantification. Briefly, FASTQ sequences were processed through our quality filter tool ensuring 100% sequences must retained a Phred value of ≥ 40 (P = − log_10_(Q)). Next the sequences are annotated and demultiplexed with our IgMatcher tool to identify the scaffold (germline) assignment and to annotate using IMGT® annotation for LCDR1, LCDR3 and HCDR1-3 and KABAT for LCDR2. Demultiplexing uses the barcode table outlined in Supplementary Table 1, with a % identity of ≥ 70% (maximum of 2 out of 8 mismatches for 8mer barcode) for both 5’ and 3’ barcodes. In Orion, each workflow produces reports with relevant visual summaries and statistics, as well as datasets that are easily analyzed and can be used to sub select, and/or carried through subsequent workflows, or exported to human readable formats, when necessary, e.g. when needing to send final sequences to vendors for production of the selected antibodies.

### Clustering of antibody sequences

Select regions of interest (e.g., HCDR3) are clustered using an internal unsupervised machine learning approach based upon sequence-based properties, NGS statistics (e.g., relative abundance and round-to-round enrichment) based on different regions of interest (HCDR3, HCDR3 + LCDR3, concatenated CDRs) and algorithms (Elbow method^31,32^; Ordering Points to Identify the Clustering Structure (OPTICS)^33^, physicochemical reduction of the amino acid space, traditional clonotyping and Levenshtein distance (LD). The combination of all these features is our clustering method we call AbScan*.* All annotated CDRs at the amino acid level into eleven representative physicochemical pseudo-sequences (Supplementary Table 5). Next, using the LD of this reduced space, we generated matrices of pairwise LD across all defined CDR regions of interest, which were subject to the OPTICS algorithm (sklearn.cluster.OPTICS) to identify core samples of high density, which takes two hyperparameters of min_pts and max_eps. While OPTICS does not require the max_eps parameter (default t = np.inf), which would effectively identify clusters across all scales, we restrict the max_eps to 10 × the optimal epsilon value, determined using the Elbow method on a k-nearest neighbor distances in a matrix of points. The basic premise is to determine the average distances of every point to the k-nearest neighbor (set to min_pts = 2). Plotting k-distances in ascending order, the “knee” of the plot indicates the optimal eps parameter, which is the sharp change that occurs along the k-distance curve. Clusters are then extracted using the ‘xi’ parameter, which determines the minimum steepness on the reachability plot, with a default value of 0.01. Due to inherent biases of population distributions from different selection campaigns and target concentrations, we also incorporate varying iterations of the relative abundance frequency to optimize clustering of populations based on both physicochemical properties and the Levenshtein distance. To this end, we perform multiple iterations of clustering based on decreasing the percent relative frequency of the region of interest, whereby seed rounds of clustered sequence require an abundance of ≥ 0.005% followed by subsequent drop in frequency for each iteration. To ensure that highly dominant, yet distinct sequences are not rejected by OPTICS (− 1 assignment), we institute additional requirements, whereby a given CDR must exhibit minimum count within its respective population (e.g., reduced space representation of AA for the HCDR3 region with count ≥ 2) or within a pre-defined threshold (e.g., traditional clonotyping defined by same length and ≥ 90% identity), which is then assigned its own unique cluster ID. Any sequence not fulfilling these additional requirements are considered “True” noise points and discarded. All CDRs are assigned a cluster ID according to Supplementary Table 4 for the specified region(s), with HCDR3 the region of interest used in much of the paper for clustering unless specified otherwise.

### XGBoost classification and regression

We determined the NGS-based statistic for all SPR (LSA) characterized antibodies within our dataset. Due to the small size of the dataset and need to validate model through a bootstrap method, the dataset were partitioned into 100/100 train/test split using 200 characterized antibodies within the dataset (Supplementary Fig. 13). To get a better sense of model variability in the training set we employed a bootstrap resampling of the training dataset (Supplementary Fig. 13a) with AUC and PRAUC reported in (Supplementary Fig. 13b–c) under binary classification and regression settings**.**

The descriptors used in the supervised XGBoost model include (1) non-sequence-based NGS descriptors by the region of interest (Supplementary Table 8), (2) relatively constant sequence-based features using one-hot encoding of the framework regions (Supplementary Table 9). For binary classification, antibodies were considered binders if an antibody exhibited < 1 µM affinity against any one of the RBD, S1 or trimer antigen. For regression prediction, the response labels for each characterized clone used the LSA-derived affinity against the monomeric target of RBD or S1.

### Enrichment and % region of interest calculation (ROI)

With the annotated records, we used the read count and different ROIs (e.g., HCDR3, HCDR3 + LCDR3, LCDR1-3/HCDR1-3) across separate barcode populations to calculate the relative frequency and enrichment. Using HCDR3 ROI as an example: for each unique HCDR3 belonging to a distinct barcode group (RBD, S1, trimer) and concentration (1 nM or 10 nM), though distinct in other regions of the antibody, we calculated the relative frequency by condensing all identical (100% identity) HCDR3s and summing the counts that belonged to each unique full-length (VH + VL) sequence in the population. We then used the simple frequency equation to tabulate the relative frequency of the ith unique ROI by barcode group and concentration:

$${\text{Relative frequency}}_{i} = \frac{{ROI_{{count_{i} }} }}{{SUM\left( {ROI_{count} } \right)}} \times 100$$

where $RO{I}_{coun{t}_{i}}$ is the sum of all the read counts that share the same ROI in given barcode group and SUM(ROIcount) is the sum of all counts in the corresponding barcode group. The ROI relative frequencies from the late round population (1 nM) were then compared to early round populations (10 nM) to obtain a relative enrichment. Again, using HCDR3 ROI as an example: matching HCDR3 from the 1 nM round that share identical (100% identity) HCDR3 in 10 nM ROI had enrichment calculated as follows:

$${\text{Enrichment}}_{i} = \frac{{{\text{Relative frequency}}_{{1nM_{i} }} }}{{{\text{Relative frequency}}_{{10nM_{i} }} }} \times 100$$

where relative_frequency_1nM_i_ is the relative_frequency tabulated using a given ROI by barcode group (RBD, S1, trimer) at the 1 nM concentration while relative_frequency_10nM_i_ is the relative_frequency tabulated using a given ROI by barcode group (RBD, S1, trimer) at the 10 nM concentration. We apply a correction factor to ROIs that appear in one population (e.g., 1 nM) but not the other (e.g. 10 nM) by taking the minimum ROI count from population it does not appear and dividing by this penalty. For instance, if clone appears in the late round (10 nM) but not the early round (1 nM) then the minimum ROI count from early round is divided by a correction factor:

$${\text{Corrected frequency}}_{absent\_i} = \frac{{\left( {{\raise0.7ex\hbox{${{\text{min}}\left( {ROI_{{count_{i} }} } \right)}$} \!\mathord{\left/ {\vphantom {{{\text{min}}\left( {ROI_{{count_{i} }} } \right)} {{\text{Correction}}\_{\text{factor}}}}}\right.\kern-0pt} \!\lower0.7ex\hbox{${{\text{Correction}}\_{\text{factor}}}$}}} \right)}}{{SUM\left( {ROI_{count} } \right)}} \times 100$$

whereby the minimum count of a given population and concentration to which it is absent is divided by a correction factor before calculation of the relative frequency. For instance, if a given ROI is present in 1 nM but not 10 nM sort, then the minimum value from 10 nM population is obtained (e.g., count = 1). If the ROI is absent in 10 nM sort but not the 1 nM sort, we use a correction of 2. If the ROI is absent in 1 nM but present in 10 nM then we apply a correction factor of 10. In this way we penalize depletion more heavily than the benefit for enrichment more significant enrichment.

### IgG reformatting expression and purification

The variable fragments corresponding to the heavy and light chain of the antibodies identified during the Sanger sequencing screening where subclined into a 2-vector system (one for the heavy chain and one for the light chain, expressed and purified as described in Leal et al. (manuscript under revision). For remaining antibodies, we outsourced at Genscript who supplied antibodies at 100 µg/mL concentration.

### LSA kinetics

The kinetics were carried out using an HC200M sensor chip (Carterra #4297), which was activated with 33 mM *N*-hydroxysulfoccinimide (S-NHS, sigma #56,485), 133 mM *N*-(3-Dimethylaminopropyl)-*N*′-ethylcarbodiimide hydrochloride (EDC, sigma #E7750) diluted in 0.1 M MES, pH 5.5 for 5 min. The capture antibody, an anti-human Fc (Southern Biotecch, #2048–01) was diluted to 50 µg/ml in 10 mM acetate, pH 4.33, and immobilized on chip for 20 min. The chip surface was deactivated using 1 M ethanolamine solution, pH 8.5, to prevent any additional primary amine coupling. Individual antibody clnoes were diluted to 10 µg/mL in 1xHBSTE (Carterra #3630) printed onto the chip for 12 min. The rbd (acro biosystems #SPD-C52H3)**,** s1 (acro biosystems #S1N-C82E8)**,** or trimer (acro biosystems #SPD-C52H9)**,** was prepared across a 7-point dilution series from 100 nM to 137 pM (Trimer) or 300 nM to 411 pM (RBD, S1). All data was fit using the Kinetics software suite (Carterra) using a one-site model.

### LSA binning

For each kinetics experiment an HC30M sensor ship (Carterra #4279) was activated with 33 mM *N*-hydroxysulfoccinimide (S-NHS, sigma #56,485), 133 mM *N*-(3-Dimethylaminopropyl)-*N*′-ethylcarbodiimide hydrochloride (EDC, sigma #E7750) diluted in 0.1 M MES, pH 5.5 for 5 min. All ligand antibodies (panel of antibodies to be tested) were diluted in 10 mM NaAcetate, pH 4.33 with 0.01% tween. All antibodies were coupled at 10 µg/mL in a 96-well plate, with the S-NHS/EDC occurring over a 5 min period with coupling to ligand mAbs at 10 min. Hydrolysis to unused S-NHS esters back to original form was performed with 50 mM borate buffer for 15 min followed by a 1 M ethanolamine wash or 2 × 30 s to block any potential non-hydrolyzed sites from 50 mM Borate wash. From prior studies we determine that use of glycine, pH 2.8 with 1 M NaCl was optimal for regeneration. Different antigen concentrations were used, typically at 20 × the K_D_ of the antibodies used in the panel to ensure saturation. Analyte mAbs at 30 µg/mL were diluted in running buffer (1xHBSTE + 0.5 mg/mL BSA. Injections were to occur every 12 cycles with a panel of ~ 200 antibodies tested within each of the runs. Injection times were set to 1 min baseline, 4 min antigen injection, 4 min for mAb analyte and 1 min of dissociation. 2 × 20 s regeneration cycles were run to regenerate the chip surface.

### Supplementary Information

Supplementary Information 1.

Supplementary Information 2.

Supplementary Information 3.

## Data Availability

Any additional information, datasets used and/or analyzed during the current study, are available from the corresponding author on reasonable request. The datasets generated and/or analyzed during the study are available at GenBank https://www.ncbi.nlm.nih.gov/genbank/ with accession numbers provided in supplementary file called “GenBank_FlatFile.txt” from range OR488140—OR488539.
