# Author Correction: Insights into next generation sequencing guided antibody selection strategies

**DOI:** 10.1038/s41598-023-49214-x

**Published:** 2023-12-18

**Authors:** M. Frank Erasmus, Fortunato Ferrara, Sara D’Angelo, Laura Spector, Camila Leal‑Lopes, André A. Teixeira, Jesper Sørensen, Suhani Nagpal, Kathryn Perea‑Schmittle, Alok Choudhary, William Honnen, David Calianese, Luis Antonio Rodriguez Carnero, Simon Cocklin, Victor Greiff, Abraham Pinter, Andrew R. M. Bradbury

**Affiliations:** 1 Specifica LLC, a Q2 Solutions Company, Santa Fe, USA; 2 New Mexico Consortium, Los Alamos, USA; 3 OpenEye, Cadence Molecular Sciences, Santa Fe, USA; 4 Public Health Research Institute, New Jersey Medical School, Rutgers, The State University of New Jersey, Newark, NJ 07103 USA; 5 University of Oslo, Oslo, Norway

Correction to: *Scientific Reports* https://doi.org/10.1038/s41598-023-45538-w, published online 26 October 2023

The original version of this Article contained an error in Reference 11, which was incorrectly given as:

(!!! INVALID CITATION !!! 3).

It has now been deleted. As a result, the subsequent References have been renumbered.

In addition, the supplementary binding data was omitted from the Supplementary Information file. It now accompanies the original Article.

The original Article has been corrected.

