# Author Correction: Insights into next generation sequencing guided antibody selection strategies

**DOI:** 10.1038/s41598-024-53751-4

**Published:** 2024-02-07

**Authors:** M. Frank Erasmus, Fortunato Ferrara, Sara D’Angelo, Laura Spector, Camila Leal-Lopes, André A. Teixeira, Jesper Sørensen, Suhani Nagpal, Kathryn Perea-Schmittle, Alok Choudhary, William Honnen, David Calianese, Luis Antonio Rodriguez Carnero, Simon Cocklin, Victor Greiff, Abraham Pinter, Andrew R. M. Bradbury

**Affiliations:** 1 Specifica LLC, a Q2 Solutions Company, Santa Fe, USA; 2 New Mexico Consortium, Los Alamos, USA; 3 OpenEye, Cadence Molecular Sciences, Santa Fe, USA; 4 Public Health Research Institute, New Jersey Medical School, Rutgers, The State University of New Jersey, Newark, NJ 07103 USA; 5 University of Oslo, Oslo, Norway

Correction to: *Scientific Reports* 10.1038/s41598-023-45538-w, published online 26 October 2023

The original version of this Article contained an error in Figure 1, panel b, where the flow plot in the bottom row, left panel was incorrectly duplicated from that in the middle row, left panel. Additionally, Figure 1b has been updated to include the appropriate flow plots that were carried out at appropriate time of experiments. The original Figure 1 and accompanying legend appear below.

**Figure 1:**
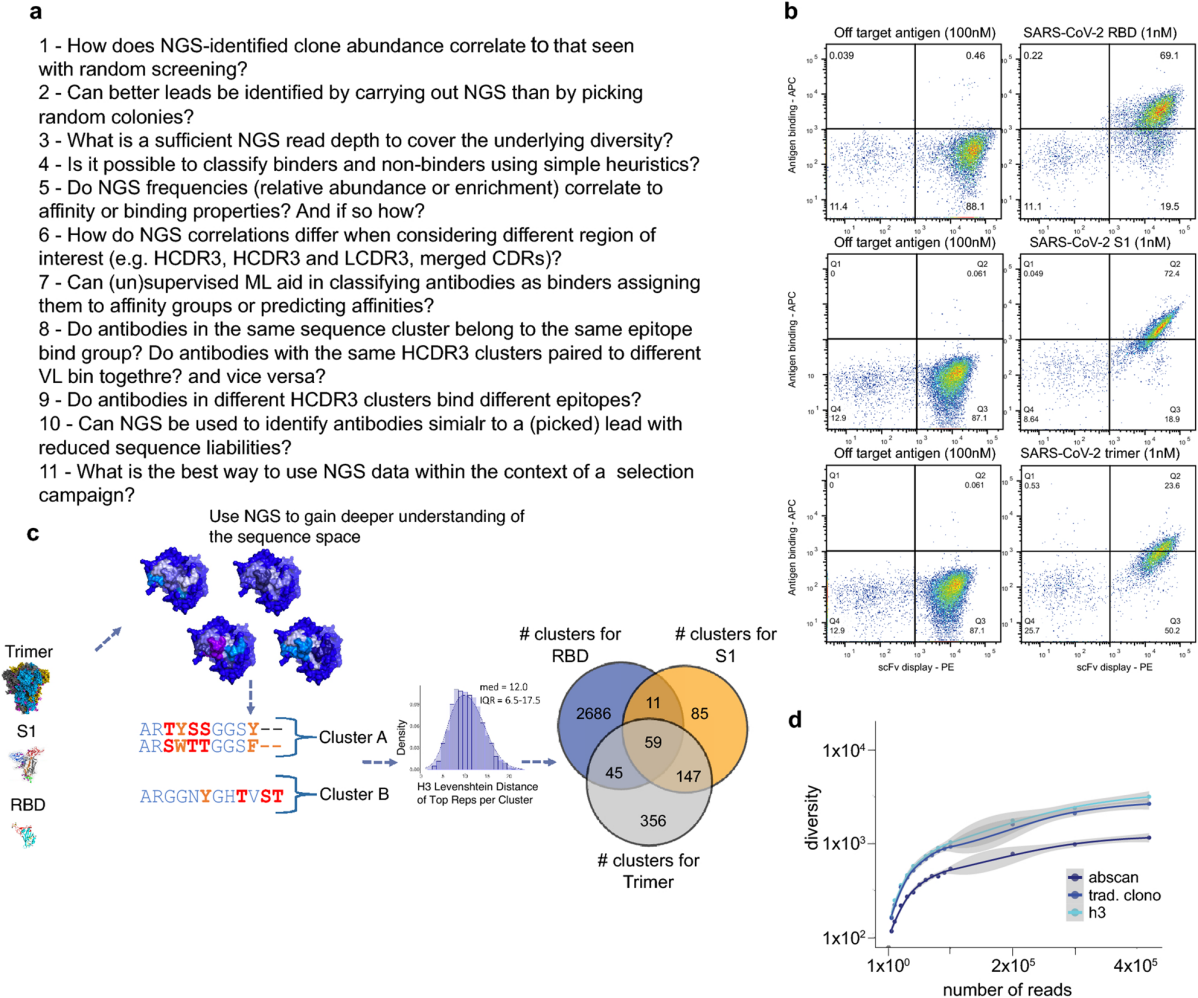
NGS-guided strategy. (**a**) Key questions relevant to any NGS-guided selection campaign (**b**) Final flow plots of yeast displayed selection outputs against RBD, S1 and trimer. (**c**) NGS-guided selection strategy and median differences among different sequences in cluster population. (**d**) Diversity accumulation by read count by given region or clustering method.

The original Article has been corrected.

