# Supplementary material for: Insights into next generation sequencing guided antibody selection strategies
Source: Sci Rep. 2023 Oct 26;13:18370. doi: 10.1038/s41598-023-45538-w (PMC10603065; doi:10.1038/s41598-023-45538-w)
Supplement: Supplementary file 2 — Supplementary Information 2. [file 41598_2023_45538_MOESM2_ESM.pptx]

## Slide 1
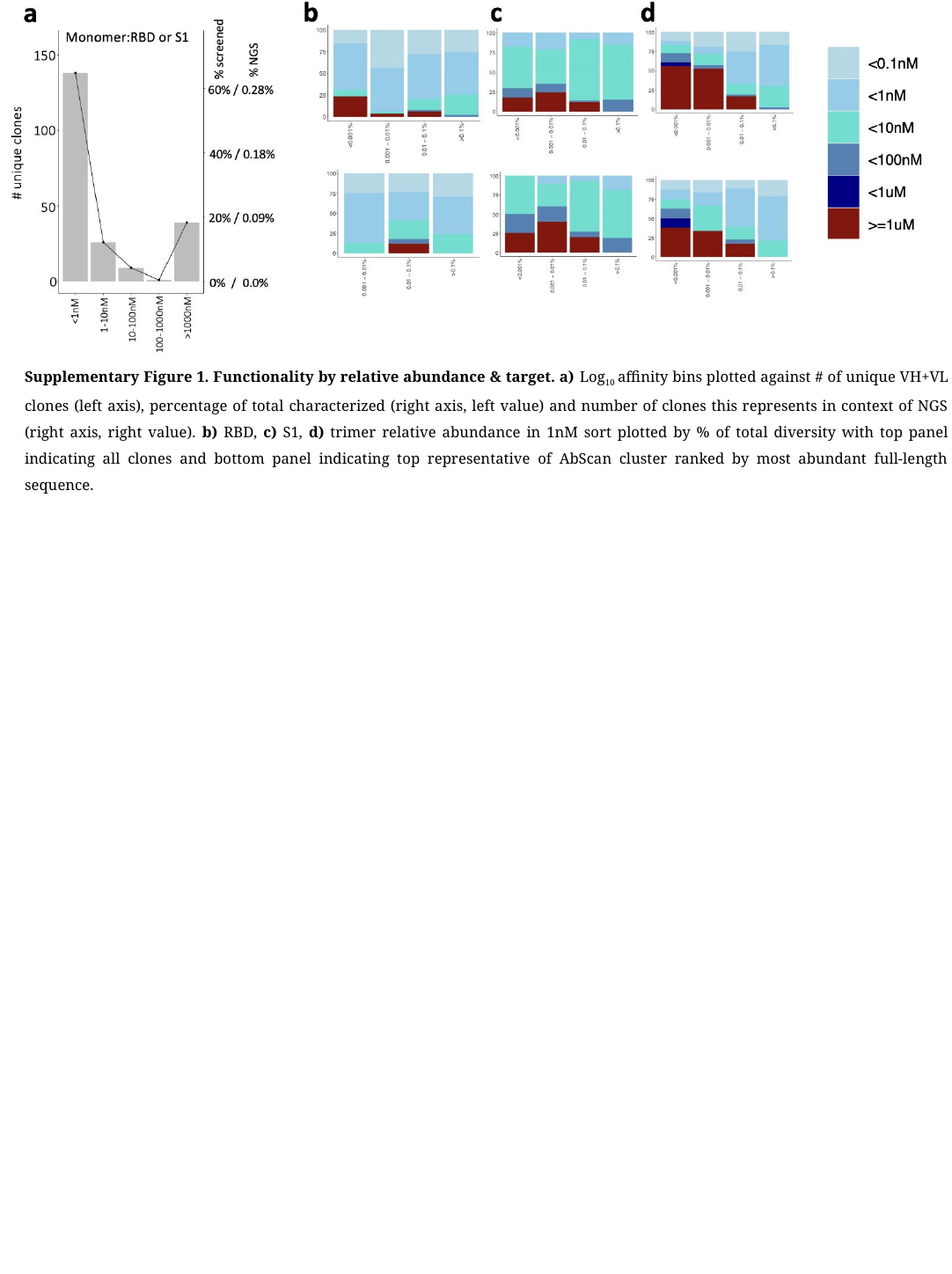

Supplementary Figure 1. Functionality by relative abundance & target. a) Log10 affinity bins plotted against # of unique VH+VL clones (left axis), percentage of total characterized (right axis, left value) and number of clones this represents in context of NGS (right axis, right value). b) RBD, c) S1, d) trimer relative abundance in 1nM sort plotted by % of total diversity with top panel indicating all clones and bottom panel indicating top representative of AbScan cluster ranked by most abundant full-length sequence.

## Slide 2
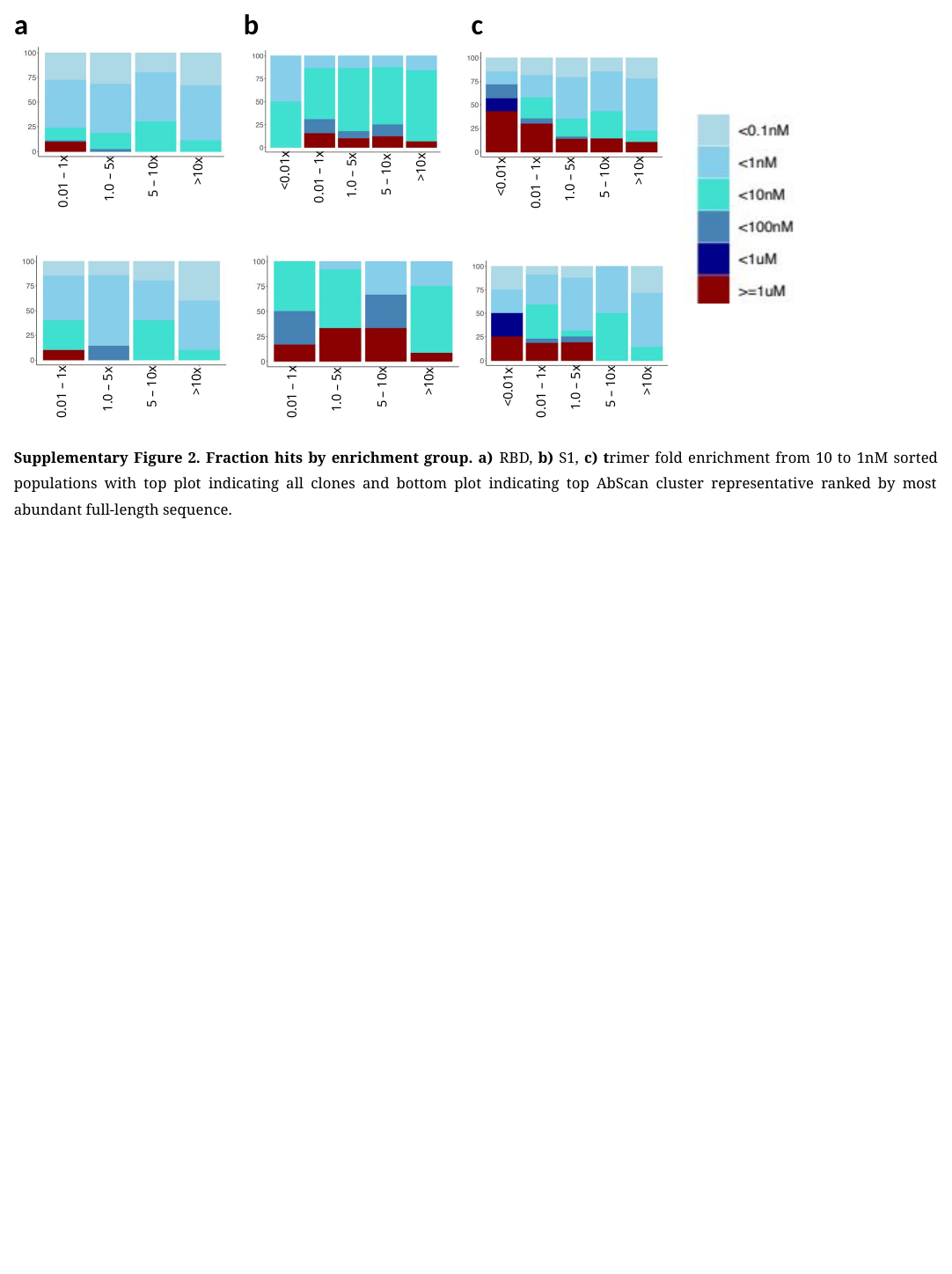

a
b
c
>10x
5 – 10x
1.0 – 5x
0.01 – 1x
>10x
5 – 10x
1.0 – 5x
0.01 – 1x
>10x
<0.01x
<0.01x
5 – 10x
1.0 – 5x
0.01 – 1x
>10x
5 – 10x
<0.01x
1.0 – 5x
0.01 – 1x
>10x
5 – 10x
1.0 – 5x
0.01 – 1x
>10x
5 – 10x
1.0 – 5x
0.01 – 1x
Supplementary Figure 2. Fraction hits by enrichment group. a) RBD, b) S1, c) trimer fold enrichment from 10 to 1nM sorted populations with top plot indicating all clones and bottom plot indicating top AbScan cluster representative ranked by most abundant full-length sequence.

## Slide 3
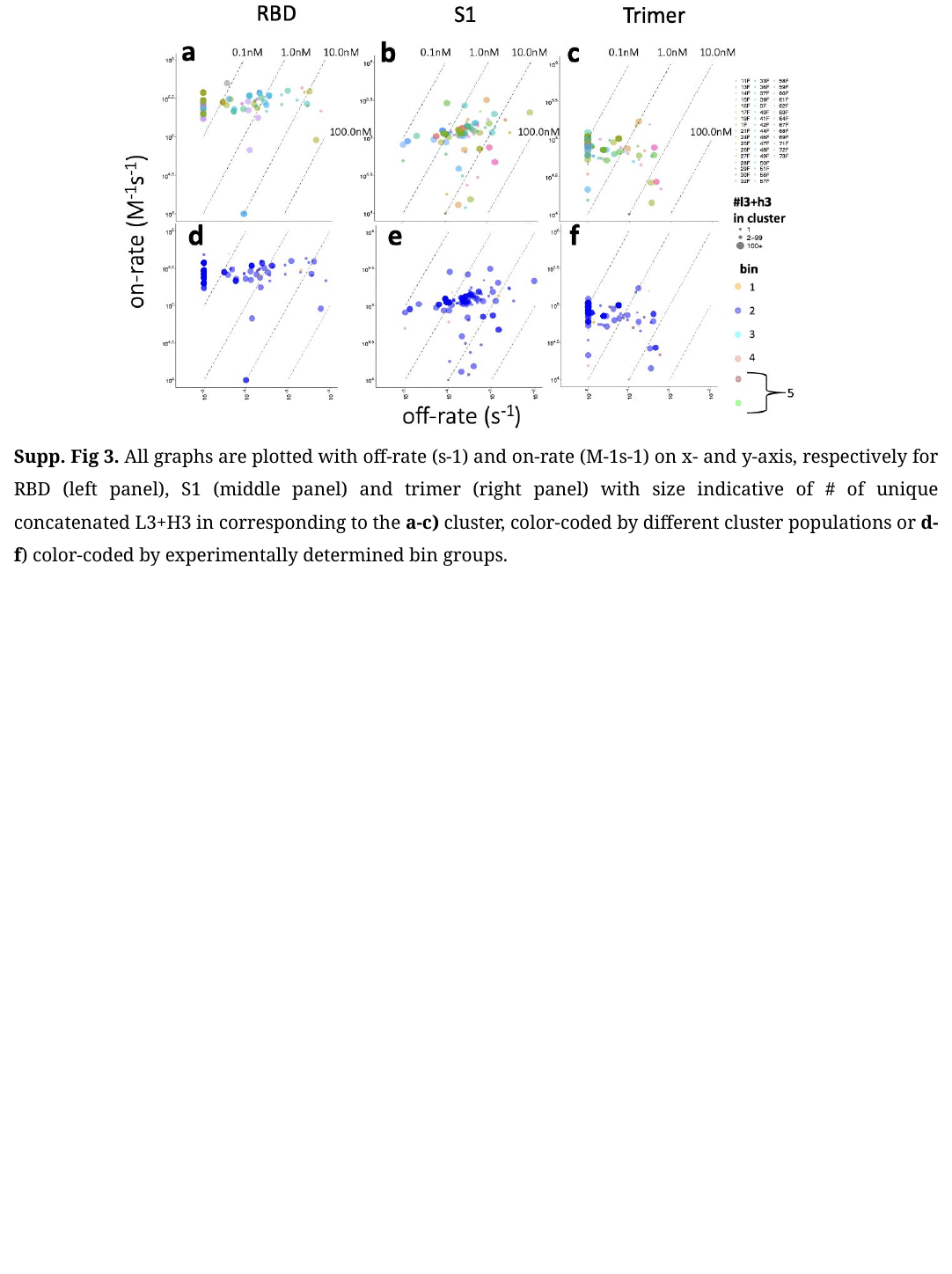

Supp. Fig 3. All graphs are plotted with off-rate (s-1) and on-rate (M-1s-1) on x- and y-axis, respectively for RBD (left panel), S1 (middle panel) and trimer (right panel) with size indicative of # of unique concatenated L3+H3 in corresponding to the a-c) cluster, color-coded by different cluster populations or d-f) color-coded by experimentally determined bin groups.

## Slide 4
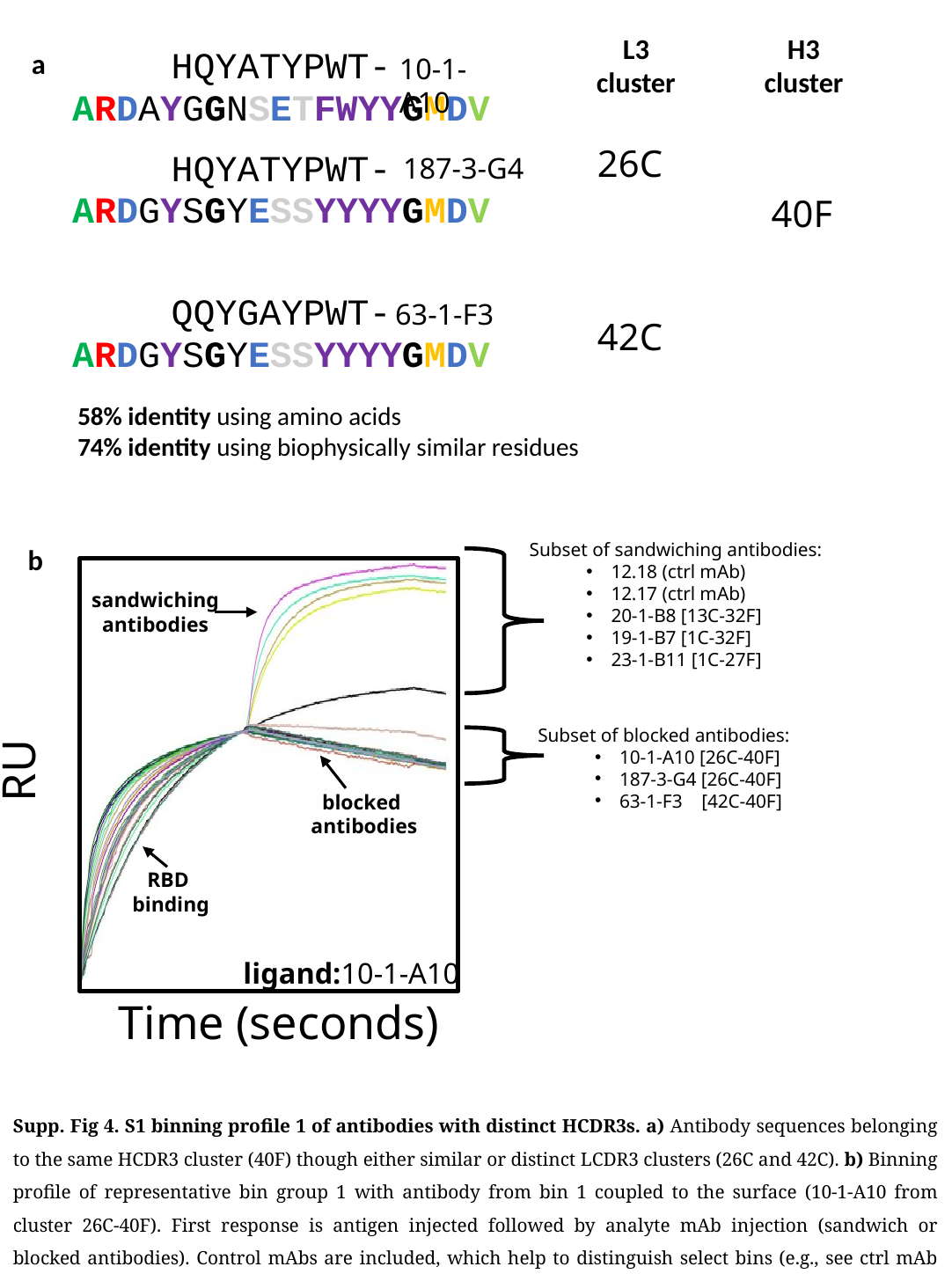

L3
cluster
H3
cluster
| HQYATYPWT-ARDAYGGNSETFWYYGMDV | 26C | 40F |
| --- | --- | --- |
| HQYATYPWT-ARDGYSGYESSYYYYGMDV | 446C | |
| QQYGAYPWT-ARDGYSGYESSYYYYGMDV | 42C | 40F |
a
10-1-A10
187-3-G4
63-1-F3
58% identity using amino acids
74% identity using biophysically similar residues
Subset of sandwiching antibodies:
12.18 (ctrl mAb)
12.17 (ctrl mAb)
20-1-B8 [13C-32F]
19-1-B7 [1C-32F]
23-1-B11 [1C-27F]
b
sandwiching antibodies
Subset of blocked antibodies:
10-1-A10 [26C-40F]
187-3-G4 [26C-40F]
63-1-F3 [42C-40F]
RU
blocked
antibodies
RBD
binding
ligand:10-1-A10
Time (seconds)
Supp. Fig 4. S1 binning profile 1 of antibodies with distinct HCDR3s. a) Antibody sequences belonging to the same HCDR3 cluster (40F) though either similar or distinct LCDR3 clusters (26C and 42C). b) Binning profile of representative bin group 1 with antibody from bin 1 coupled to the surface (10-1-A10 from cluster 26C-40F). First response is antigen injected followed by analyte mAb injection (sandwich or blocked antibodies). Control mAbs are included, which help to distinguish select bins (e.g., see ctrl mAb 12.18).

## Slide 5
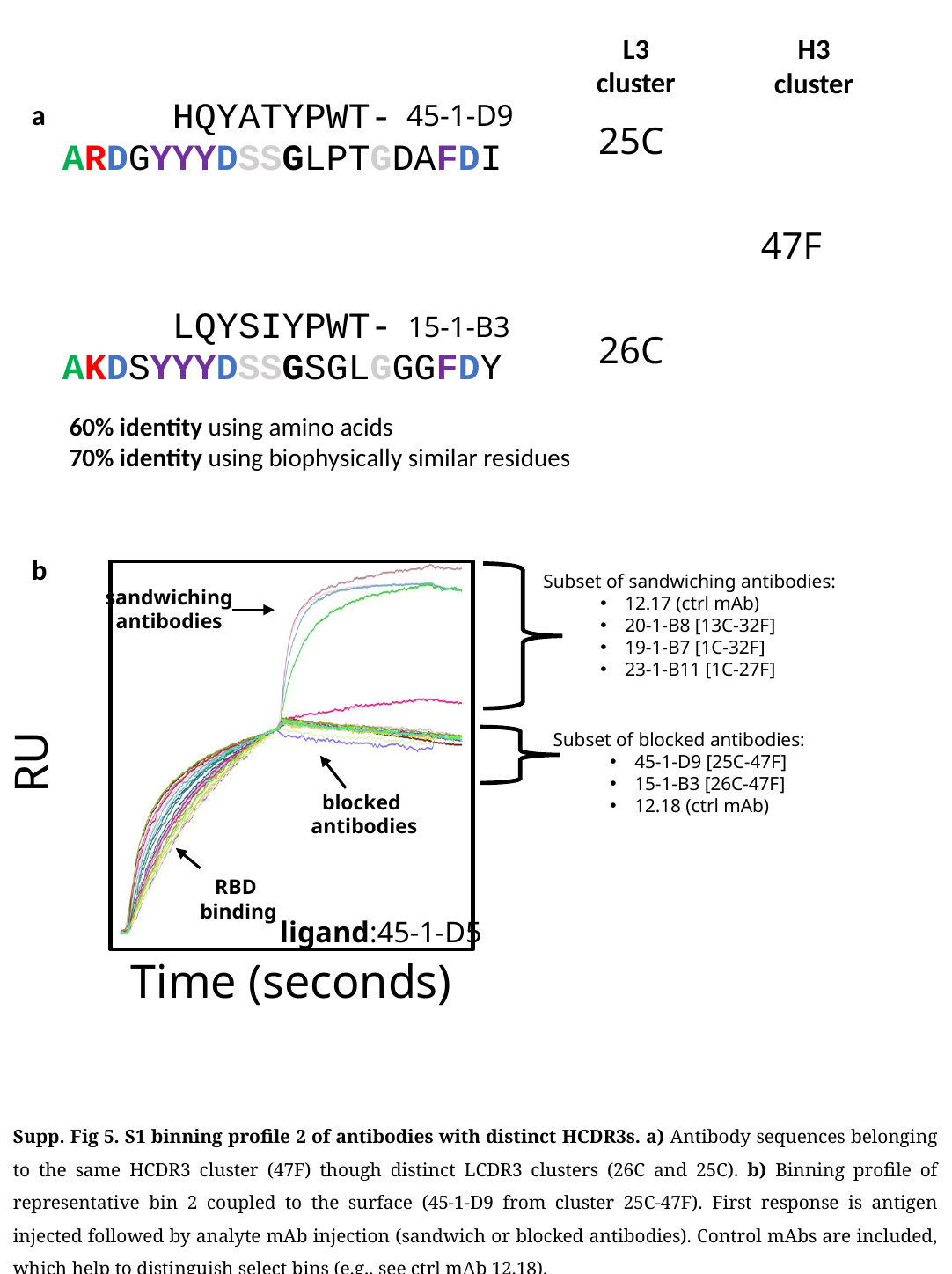

L3
cluster
H3
cluster
| HQYATYPWT- ARDGYYYDSSGLPTGDAFDI | 25C | 47F |
| --- | --- | --- |
| LQYSIYPWT-AKDSYYYDSSGSGLGGGFDY | 26C | |
a
45-1-D9
15-1-B3
60% identity using amino acids
70% identity using biophysically similar residues
b
RU
Time (seconds)
Subset of sandwiching antibodies:
12.17 (ctrl mAb)
20-1-B8 [13C-32F]
19-1-B7 [1C-32F]
23-1-B11 [1C-27F]
sandwiching antibodies
Subset of blocked antibodies:
45-1-D9 [25C-47F]
15-1-B3 [26C-47F]
12.18 (ctrl mAb)
blocked
antibodies
RBD
binding
ligand:45-1-D5
Supp. Fig 5. S1 binning profile 2 of antibodies with distinct HCDR3s. a) Antibody sequences belonging to the same HCDR3 cluster (47F) though distinct LCDR3 clusters (26C and 25C). b) Binning profile of representative bin 2 coupled to the surface (45-1-D9 from cluster 25C-47F). First response is antigen injected followed by analyte mAb injection (sandwich or blocked antibodies). Control mAbs are included, which help to distinguish select bins (e.g., see ctrl mAb 12.18).

## Slide 6
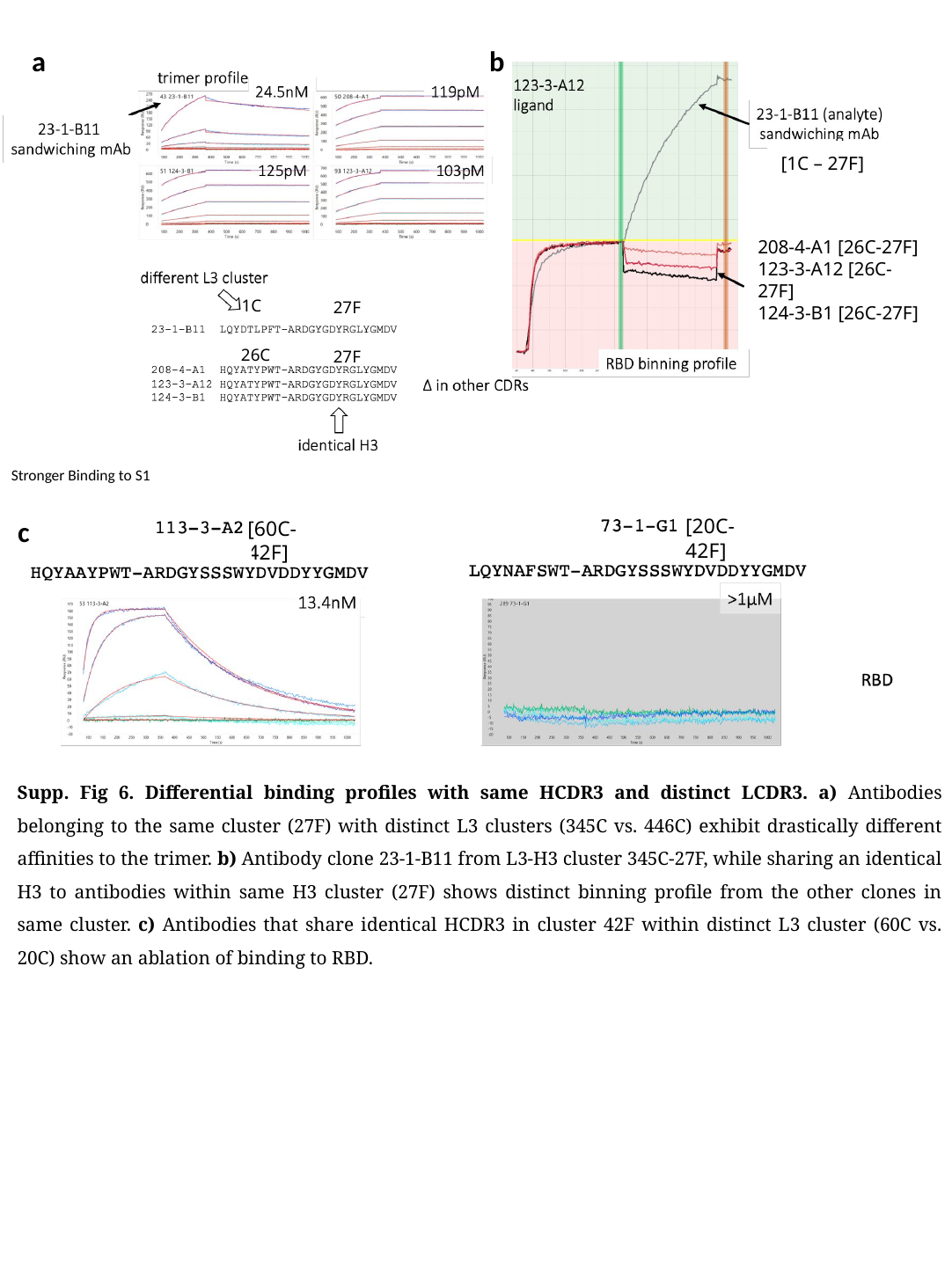

a
b
[1C – 27F]
208-4-A1 [26C-27F]
123-3-A12 [26C-27F]
124-3-B1 [26C-27F]
1C
27F
26C
27F
Stronger Binding to S1
c
[20C-42F]
[60C-42F]
Supp. Fig 6. Differential binding profiles with same HCDR3 and distinct LCDR3. a) Antibodies belonging to the same cluster (27F) with distinct L3 clusters (345C vs. 446C) exhibit drastically different affinities to the trimer. b) Antibody clone 23-1-B11 from L3-H3 cluster 345C-27F, while sharing an identical H3 to antibodies within same H3 cluster (27F) shows distinct binning profile from the other clones in same cluster. c) Antibodies that share identical HCDR3 in cluster 42F within distinct L3 cluster (60C vs. 20C) show an ablation of binding to RBD.

## Slide 7
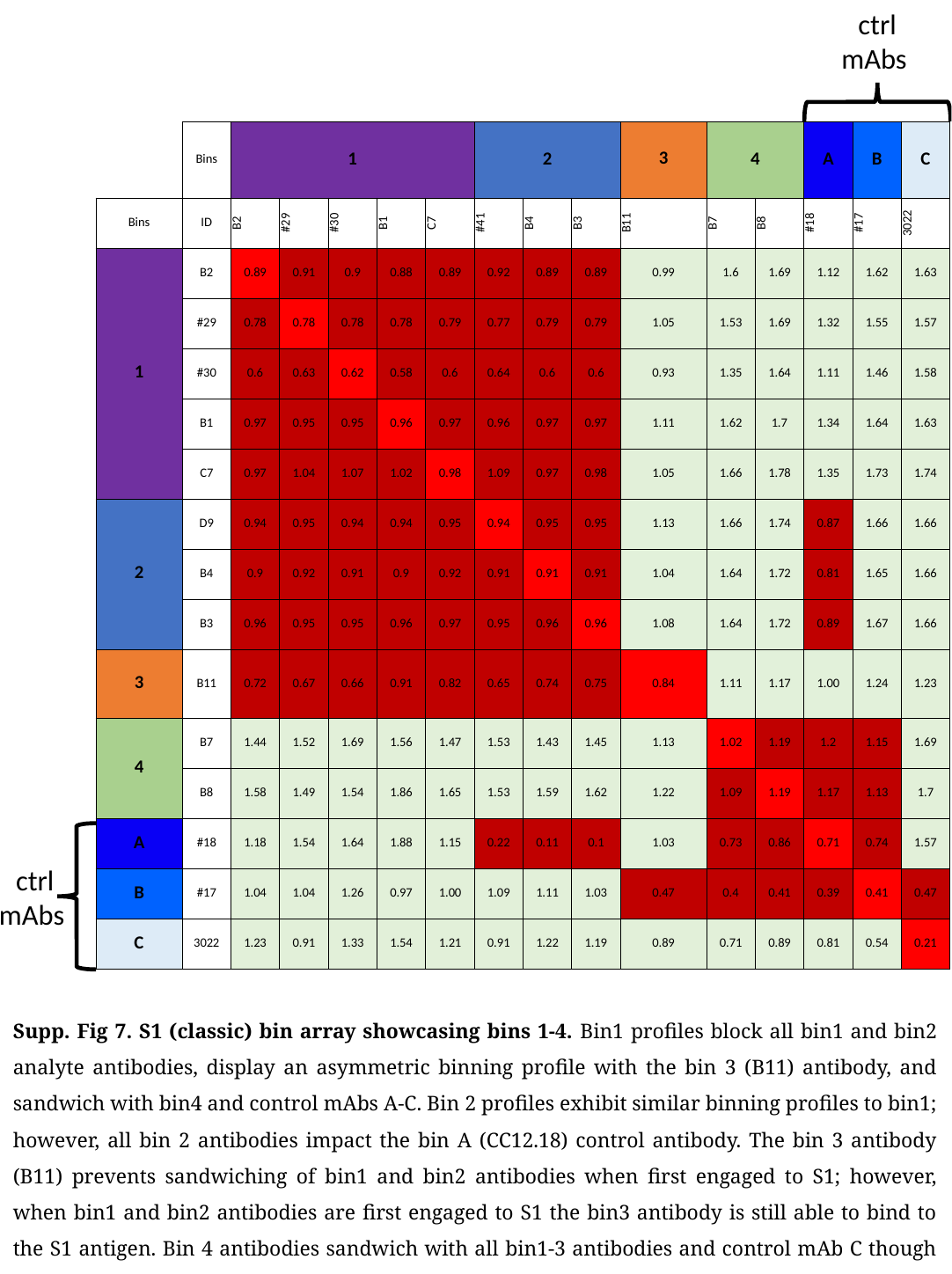

ctrl
mAbs
| | Bins | 1 | | | | | 2 | | | 3 | 4 | 7 | A | B | C |
| --- | --- | --- | --- | --- | --- | --- | --- | --- | --- | --- | --- | --- | --- | --- | --- |
| Bins | ID | B2 | #29 | #30 | B1 | C7 | #41 | B4 | B3 | B11 | B7 | B8 | #18 | #17 | 3022 |
| 1 | B2 | 0.89 | 0.91 | 0.9 | 0.88 | 0.89 | 0.92 | 0.89 | 0.89 | 0.99 | 1.6 | 1.69 | 1.12 | 1.62 | 1.63 |
| | #29 | 0.78 | 0.78 | 0.78 | 0.78 | 0.79 | 0.77 | 0.79 | 0.79 | 1.05 | 1.53 | 1.69 | 1.32 | 1.55 | 1.57 |
| | #30 | 0.6 | 0.63 | 0.62 | 0.58 | 0.6 | 0.64 | 0.6 | 0.6 | 0.93 | 1.35 | 1.64 | 1.11 | 1.46 | 1.58 |
| | B1 | 0.97 | 0.95 | 0.95 | 0.96 | 0.97 | 0.96 | 0.97 | 0.97 | 1.11 | 1.62 | 1.7 | 1.34 | 1.64 | 1.63 |
| | C7 | 0.97 | 1.04 | 1.07 | 1.02 | 0.98 | 1.09 | 0.97 | 0.98 | 1.05 | 1.66 | 1.78 | 1.35 | 1.73 | 1.74 |
| 2 | D9 | 0.94 | 0.95 | 0.94 | 0.94 | 0.95 | 0.94 | 0.95 | 0.95 | 1.13 | 1.66 | 1.74 | 0.87 | 1.66 | 1.66 |
| | B4 | 0.9 | 0.92 | 0.91 | 0.9 | 0.92 | 0.91 | 0.91 | 0.91 | 1.04 | 1.64 | 1.72 | 0.81 | 1.65 | 1.66 |
| | B3 | 0.96 | 0.95 | 0.95 | 0.96 | 0.97 | 0.95 | 0.96 | 0.96 | 1.08 | 1.64 | 1.72 | 0.89 | 1.67 | 1.66 |
| 3 | B11 | 0.72 | 0.67 | 0.66 | 0.91 | 0.82 | 0.65 | 0.74 | 0.75 | 0.84 | 1.11 | 1.17 | 1.00 | 1.24 | 1.23 |
| 4 | B7 | 1.44 | 1.52 | 1.69 | 1.56 | 1.47 | 1.53 | 1.43 | 1.45 | 1.13 | 1.02 | 1.19 | 1.2 | 1.15 | 1.69 |
| | B8 | 1.58 | 1.49 | 1.54 | 1.86 | 1.65 | 1.53 | 1.59 | 1.62 | 1.22 | 1.09 | 1.19 | 1.17 | 1.13 | 1.7 |
| A | #18 | 1.18 | 1.54 | 1.64 | 1.88 | 1.15 | 0.22 | 0.11 | 0.1 | 1.03 | 0.73 | 0.86 | 0.71 | 0.74 | 1.57 |
| B | #17 | 1.04 | 1.04 | 1.26 | 0.97 | 1.00 | 1.09 | 1.11 | 1.03 | 0.47 | 0.4 | 0.41 | 0.39 | 0.41 | 0.47 |
| C | 3022 | 1.23 | 0.91 | 1.33 | 1.54 | 1.21 | 0.91 | 1.22 | 1.19 | 0.89 | 0.71 | 0.89 | 0.81 | 0.54 | 0.21 |
ctrl
mAbs
Supp. Fig 7. S1 (classic) bin array showcasing bins 1-4. Bin1 profiles block all bin1 and bin2 analyte antibodies, display an asymmetric binning profile with the bin 3 (B11) antibody, and sandwich with bin4 and control mAbs A-C. Bin 2 profiles exhibit similar binning profiles to bin1; however, all bin 2 antibodies impact the bin A (CC12.18) control antibody. The bin 3 antibody (B11) prevents sandwiching of bin1 and bin2 antibodies when first engaged to S1; however, when bin1 and bin2 antibodies are first engaged to S1 the bin3 antibody is still able to bind to the S1 antigen. Bin 4 antibodies sandwich with all bin1-3 antibodies and control mAb C though prevent binding of control antibodies from bin A and B.

## Slide 8
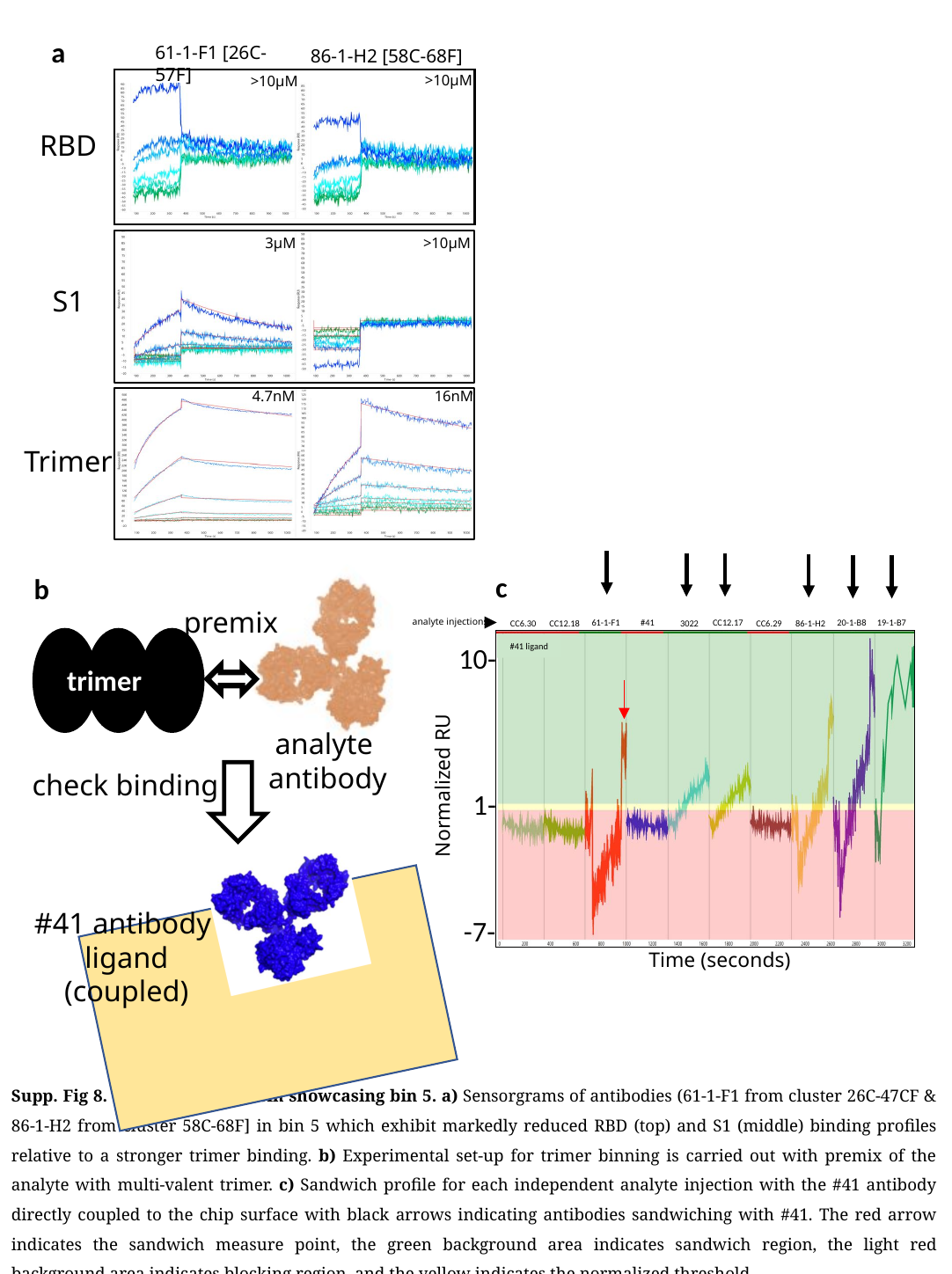

a
c
b
premix
analyte injections
61-1-F1
#41
CC12.17
19-1-B7
20-1-B8
CC6.29
86-1-H2
CC12.18
3022
CC6.30
#41 ligand
trimer
10-
analyte
antibody
check binding
Normalized RU
1-
#41 antibody
ligand
(coupled)
-7-
Time (seconds)
61-1-F1 [26C-57F]
86-1-H2 [58C-68F]
>10µM
>10µM
RBD
3µM
>10µM
S1
4.7nM
16nM
Trimer
Supp. Fig 8. Trimer (premix) bin showcasing bin 5. a) Sensorgrams of antibodies (61-1-F1 from cluster 26C-47CF & 86-1-H2 from cluster 58C-68F] in bin 5 which exhibit markedly reduced RBD (top) and S1 (middle) binding profiles relative to a stronger trimer binding. b) Experimental set-up for trimer binning is carried out with premix of the analyte with multi-valent trimer. c) Sandwich profile for each independent analyte injection with the #41 antibody directly coupled to the chip surface with black arrows indicating antibodies sandwiching with #41. The red arrow indicates the sandwich measure point, the green background area indicates sandwich region, the light red background area indicates blocking region, and the yellow indicates the normalized threshold.

## Slide 9
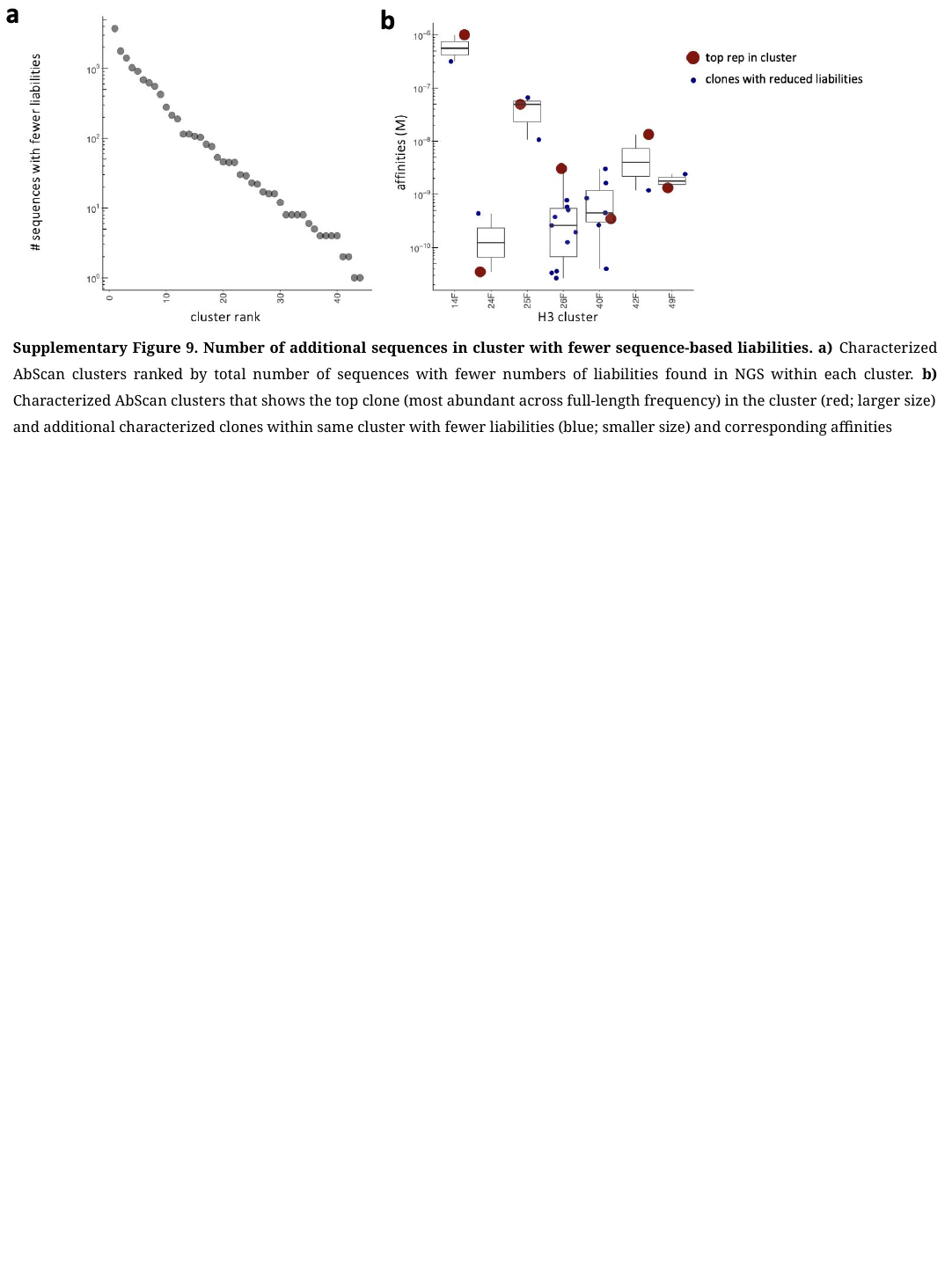

Supplementary Figure 9. Number of additional sequences in cluster with fewer sequence-based liabilities. a) Characterized AbScan clusters ranked by total number of sequences with fewer numbers of liabilities found in NGS within each cluster. b) Characterized AbScan clusters that shows the top clone (most abundant across full-length frequency) in the cluster (red; larger size) and additional characterized clones within same cluster with fewer liabilities (blue; smaller size) and corresponding affinities

## Slide 10
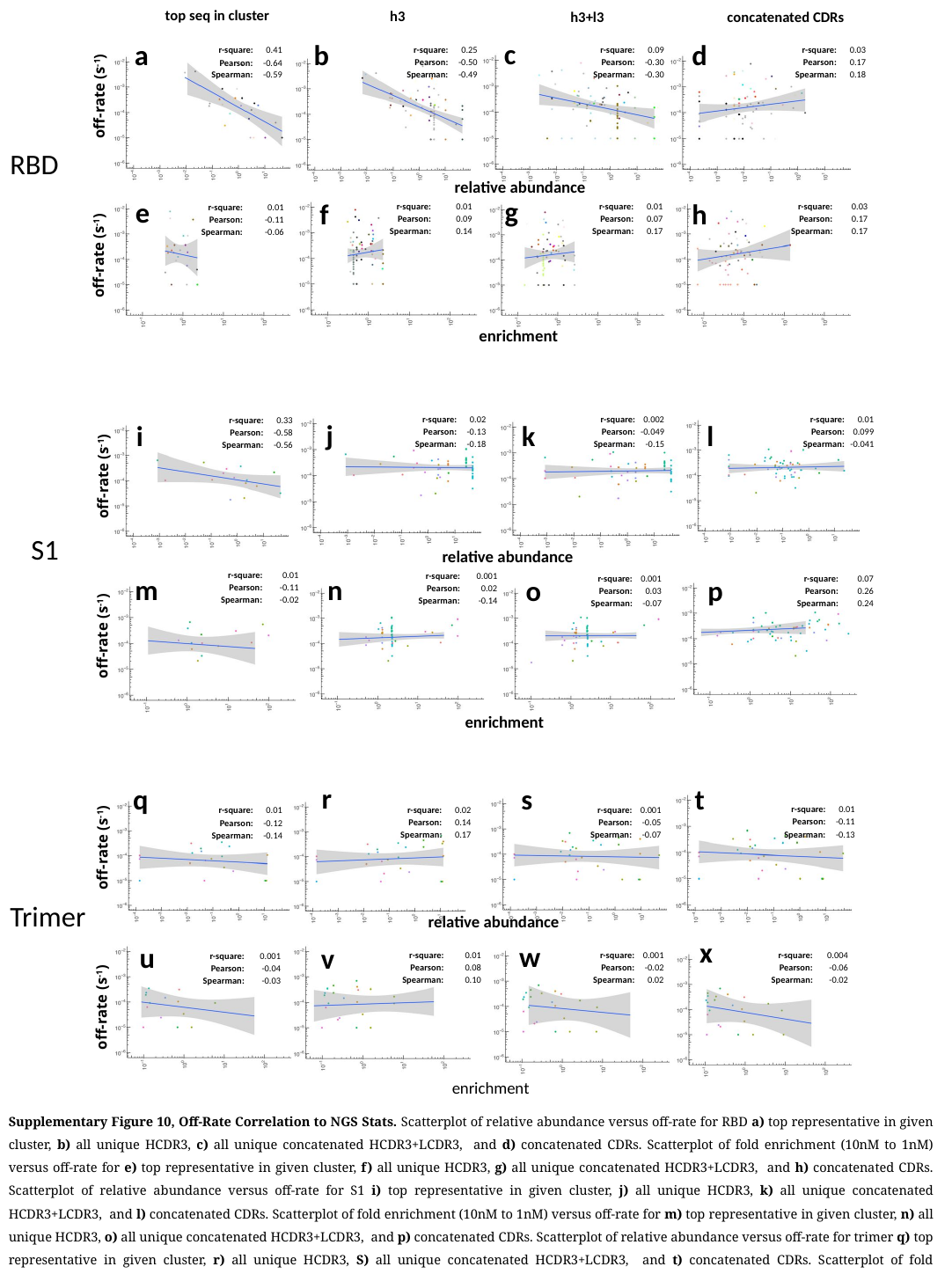

top seq in cluster
 h3
 h3+l3
concatenated CDRs
c
b
a
d
| r-square: | 0.25 |
| --- | --- |
| Pearson: | -0.50 |
| Spearman: | -0.49 |
| r-square: | 0.03 |
| --- | --- |
| Pearson: | 0.17 |
| Spearman: | 0.18 |
| r-square: | 0.09 |
| --- | --- |
| Pearson: | -0.30 |
| Spearman: | -0.30 |
| r-square: | 0.41 |
| --- | --- |
| Pearson: | -0.64 |
| Spearman: | -0.59 |
off-rate (s-1)
off-rate (s-1)
RBD
relative abundance
g
e
h
f
| r-square: | 0.01 |
| --- | --- |
| Pearson: | 0.07 |
| Spearman: | 0.17 |
| r-square: | 0.03 |
| --- | --- |
| Pearson: | 0.17 |
| Spearman: | 0.17 |
| r-square: | 0.01 |
| --- | --- |
| Pearson: | 0.09 |
| Spearman: | 0.14 |
| r-square: | 0.01 |
| --- | --- |
| Pearson: | -0.11 |
| Spearman: | -0.06 |
enrichment
j
l
i
k
| r-square: | 0.02 |
| --- | --- |
| Pearson: | -0.13 |
| Spearman: | -0.18 |
| r-square: | 0.002 |
| --- | --- |
| Pearson: | -0.049 |
| Spearman: | -0.15 |
| r-square: | 0.01 |
| --- | --- |
| Pearson: | 0.099 |
| Spearman: | -0.041 |
| r-square: | 0.33 |
| --- | --- |
| Pearson: | -0.58 |
| Spearman: | -0.56 |
off-rate (s-1)
off-rate (s-1)
S1
relative abundance
n
m
p
o
| r-square: | 0.01 |
| --- | --- |
| Pearson: | -0.11 |
| Spearman: | -0.02 |
| r-square: | 0.001 |
| --- | --- |
| Pearson: | 0.02 |
| Spearman: | -0.14 |
| r-square: | 0.07 |
| --- | --- |
| Pearson: | 0.26 |
| Spearman: | 0.24 |
| r-square: | 0.001 |
| --- | --- |
| Pearson: | 0.03 |
| Spearman: | -0.07 |
enrichment
r
t
q
s
off-rate (s-1)
off-rate (s-1)
| r-square: | 0.01 |
| --- | --- |
| Pearson: | -0.11 |
| Spearman: | -0.13 |
| r-square: | 0.02 |
| --- | --- |
| Pearson: | 0.14 |
| Spearman: | 0.17 |
| r-square: | 0.001 |
| --- | --- |
| Pearson: | -0.05 |
| Spearman: | -0.07 |
| r-square: | 0.01 |
| --- | --- |
| Pearson: | -0.12 |
| Spearman: | -0.14 |
Trimer
relative abundance
x
w
u
v
| r-square: | 0.004 |
| --- | --- |
| Pearson: | -0.06 |
| Spearman: | -0.02 |
| r-square: | 0.01 |
| --- | --- |
| Pearson: | 0.08 |
| Spearman: | 0.10 |
| r-square: | 0.001 |
| --- | --- |
| Pearson: | -0.02 |
| Spearman: | 0.02 |
| r-square: | 0.001 |
| --- | --- |
| Pearson: | -0.04 |
| Spearman: | -0.03 |
enrichment
Supplementary Figure 10, Off-Rate Correlation to NGS Stats. Scatterplot of relative abundance versus off-rate for RBD a) top representative in given cluster, b) all unique HCDR3, c) all unique concatenated HCDR3+LCDR3, and d) concatenated CDRs. Scatterplot of fold enrichment (10nM to 1nM) versus off-rate for e) top representative in given cluster, f) all unique HCDR3, g) all unique concatenated HCDR3+LCDR3, and h) concatenated CDRs. Scatterplot of relative abundance versus off-rate for S1 i) top representative in given cluster, j) all unique HCDR3, k) all unique concatenated HCDR3+LCDR3, and l) concatenated CDRs. Scatterplot of fold enrichment (10nM to 1nM) versus off-rate for m) top representative in given cluster, n) all unique HCDR3, o) all unique concatenated HCDR3+LCDR3, and p) concatenated CDRs. Scatterplot of relative abundance versus off-rate for trimer q) top representative in given cluster, r) all unique HCDR3, S) all unique concatenated HCDR3+LCDR3, and t) concatenated CDRs. Scatterplot of fold enrichment (10nM to 1nM) versus off-rate for u) top representative in given cluster, v) all unique HCDR3, w) all unique concatenated HCDR3+LCDR3, and x) concatenated CDRs.

## Slide 11
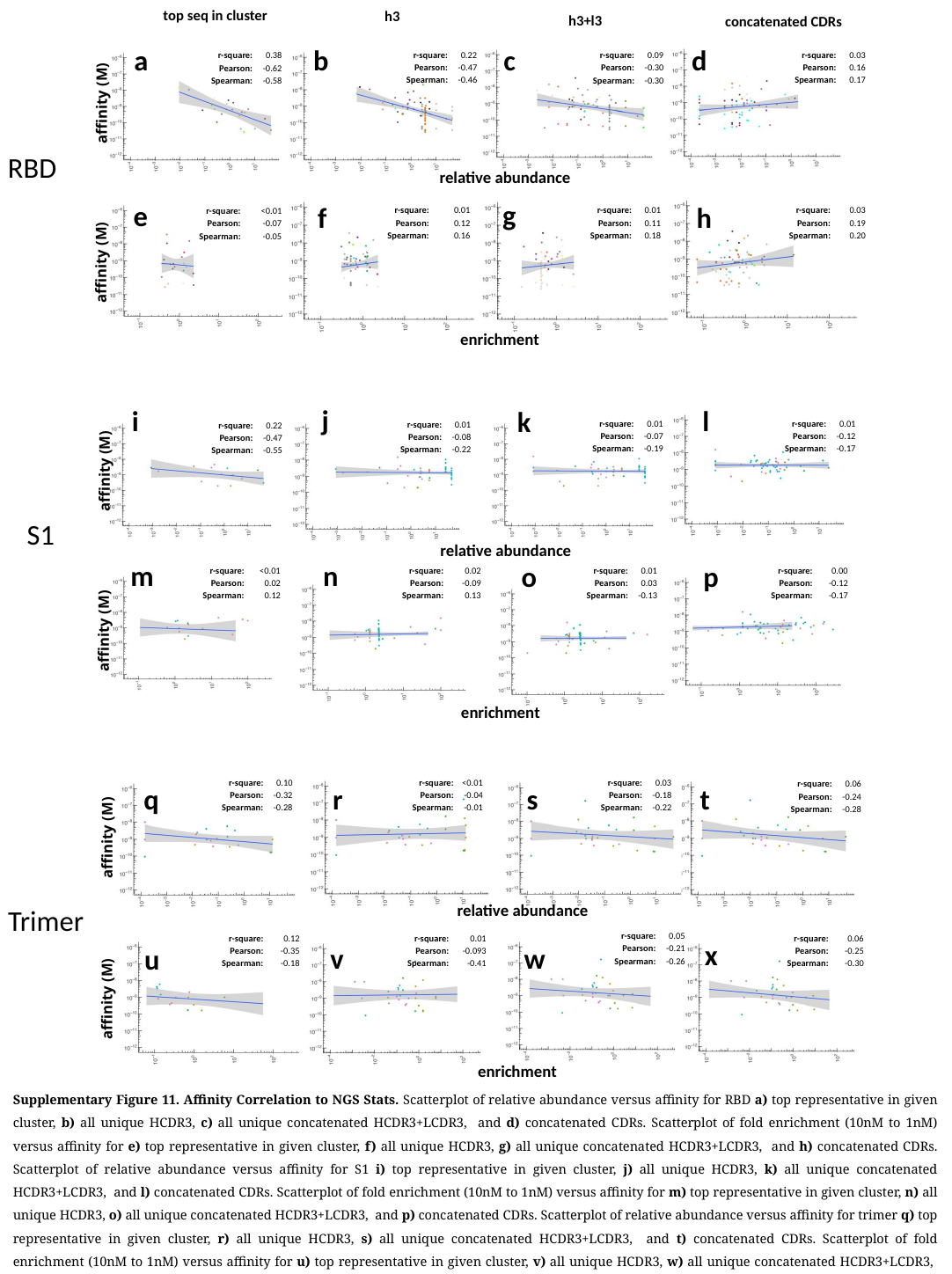

top seq in cluster
 h3
 h3+l3
concatenated CDRs
c
b
a
d
| r-square: | 0.22 |
| --- | --- |
| Pearson: | -0.47 |
| Spearman: | -0.46 |
| r-square: | 0.03 |
| --- | --- |
| Pearson: | 0.16 |
| Spearman: | 0.17 |
| r-square: | 0.09 |
| --- | --- |
| Pearson: | -0.30 |
| Spearman: | -0.30 |
| r-square: | 0.38 |
| --- | --- |
| Pearson: | -0.62 |
| Spearman: | -0.58 |
affinity (M)
affinity (M)
RBD
relative abundance
g
e
f
h
| r-square: | 0.01 |
| --- | --- |
| Pearson: | 0.11 |
| Spearman: | 0.18 |
| r-square: | 0.03 |
| --- | --- |
| Pearson: | 0.19 |
| Spearman: | 0.20 |
| r-square: | 0.01 |
| --- | --- |
| Pearson: | 0.12 |
| Spearman: | 0.16 |
| r-square: | <0.01 |
| --- | --- |
| Pearson: | -0.07 |
| Spearman: | -0.05 |
enrichment
j
l
i
k
| r-square: | 0.01 |
| --- | --- |
| Pearson: | -0.07 |
| Spearman: | -0.19 |
| r-square: | 0.01 |
| --- | --- |
| Pearson: | -0.12 |
| Spearman: | -0.17 |
| r-square: | 0.01 |
| --- | --- |
| Pearson: | -0.08 |
| Spearman: | -0.22 |
| r-square: | 0.22 |
| --- | --- |
| Pearson: | -0.47 |
| Spearman: | -0.55 |
affinity (M)
affinity (M)
S1
relative abundance
n
m
p
o
| r-square: | 0.01 |
| --- | --- |
| Pearson: | 0.03 |
| Spearman: | -0.13 |
| r-square: | 0.00 |
| --- | --- |
| Pearson: | -0.12 |
| Spearman: | -0.17 |
| r-square: | 0.02 |
| --- | --- |
| Pearson: | -0.09 |
| Spearman: | 0.13 |
| r-square: | <0.01 |
| --- | --- |
| Pearson: | 0.02 |
| Spearman: | 0.12 |
enrichment
t
s
r
q
| r-square: | 0.10 |
| --- | --- |
| Pearson: | -0.32 |
| Spearman: | -0.28 |
| r-square: | <0.01 |
| --- | --- |
| Pearson: | -0.04 |
| Spearman: | -0.01 |
| r-square: | 0.03 |
| --- | --- |
| Pearson: | -0.18 |
| Spearman: | -0.22 |
| r-square: | 0.06 |
| --- | --- |
| Pearson: | -0.24 |
| Spearman: | -0.28 |
affinity (M)
affinity (M)
relative abundance
Trimer
| r-square: | 0.05 |
| --- | --- |
| Pearson: | -0.21 |
| Spearman: | -0.26 |
x
| r-square: | 0.12 |
| --- | --- |
| Pearson: | -0.35 |
| Spearman: | -0.18 |
| r-square: | 0.01 |
| --- | --- |
| Pearson: | -0.093 |
| Spearman: | -0.41 |
| r-square: | 0.06 |
| --- | --- |
| Pearson: | -0.25 |
| Spearman: | -0.30 |
v
w
u
enrichment
Supplementary Figure 11. Affinity Correlation to NGS Stats. Scatterplot of relative abundance versus affinity for RBD a) top representative in given cluster, b) all unique HCDR3, c) all unique concatenated HCDR3+LCDR3, and d) concatenated CDRs. Scatterplot of fold enrichment (10nM to 1nM) versus affinity for e) top representative in given cluster, f) all unique HCDR3, g) all unique concatenated HCDR3+LCDR3, and h) concatenated CDRs. Scatterplot of relative abundance versus affinity for S1 i) top representative in given cluster, j) all unique HCDR3, k) all unique concatenated HCDR3+LCDR3, and l) concatenated CDRs. Scatterplot of fold enrichment (10nM to 1nM) versus affinity for m) top representative in given cluster, n) all unique HCDR3, o) all unique concatenated HCDR3+LCDR3, and p) concatenated CDRs. Scatterplot of relative abundance versus affinity for trimer q) top representative in given cluster, r) all unique HCDR3, s) all unique concatenated HCDR3+LCDR3, and t) concatenated CDRs. Scatterplot of fold enrichment (10nM to 1nM) versus affinity for u) top representative in given cluster, v) all unique HCDR3, w) all unique concatenated HCDR3+LCDR3, and x) concatenated CDRs.

## Slide 12
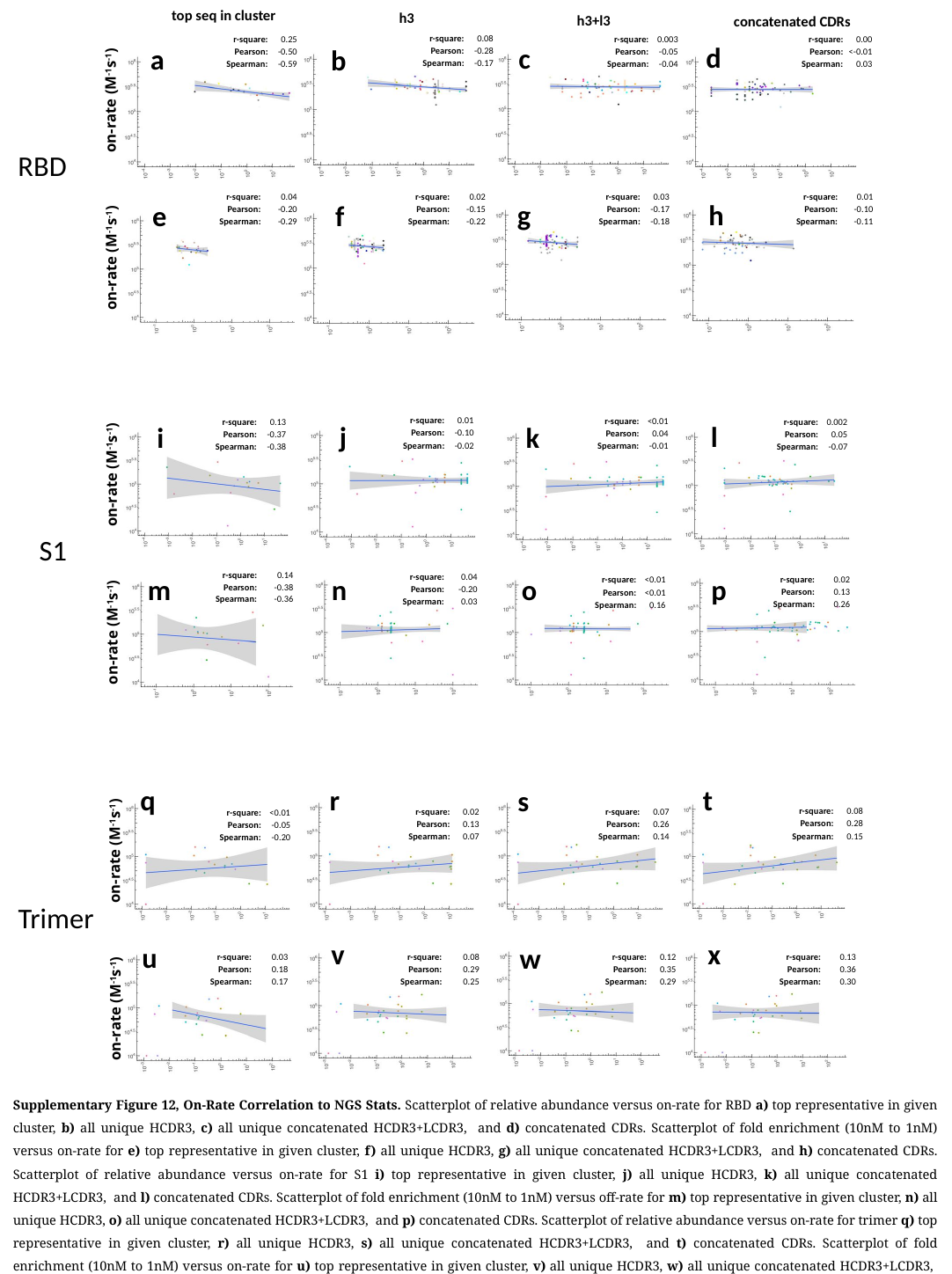

top seq in cluster
 h3
 h3+l3
concatenated CDRs
| r-square: | 0.08 |
| --- | --- |
| Pearson: | -0.28 |
| Spearman: | -0.17 |
| r-square: | 0.25 |
| --- | --- |
| Pearson: | -0.50 |
| Spearman: | -0.59 |
| r-square: | 0.003 |
| --- | --- |
| Pearson: | -0.05 |
| Spearman: | -0.04 |
d
| r-square: | 0.00 |
| --- | --- |
| Pearson: | <-0.01 |
| Spearman: | 0.03 |
c
a
b
on-rate (M-1s-1)
on-rate (M-1s-1)
RBD
| r-square: | 0.04 |
| --- | --- |
| Pearson: | -0.20 |
| Spearman: | -0.29 |
| r-square: | 0.03 |
| --- | --- |
| Pearson: | -0.17 |
| Spearman: | -0.18 |
| r-square: | 0.01 |
| --- | --- |
| Pearson: | -0.10 |
| Spearman: | -0.11 |
| r-square: | 0.02 |
| --- | --- |
| Pearson: | -0.15 |
| Spearman: | -0.22 |
h
g
e
f
j
l
i
k
on-rate (M-1s-1)
on-rate (M-1s-1)
| r-square: | 0.01 |
| --- | --- |
| Pearson: | -0.10 |
| Spearman: | -0.02 |
| r-square: | <0.01 |
| --- | --- |
| Pearson: | 0.04 |
| Spearman: | -0.01 |
| r-square: | 0.13 |
| --- | --- |
| Pearson: | -0.37 |
| Spearman: | -0.38 |
| r-square: | 0.002 |
| --- | --- |
| Pearson: | 0.05 |
| Spearman: | -0.07 |
S1
n
o
p
m
| r-square: | 0.14 |
| --- | --- |
| Pearson: | -0.38 |
| Spearman: | -0.36 |
| r-square: | 0.04 |
| --- | --- |
| Pearson: | -0.20 |
| Spearman: | 0.03 |
| r-square: | 0.02 |
| --- | --- |
| Pearson: | 0.13 |
| Spearman: | 0.26 |
| r-square: | <0.01 |
| --- | --- |
| Pearson: | <0.01 |
| Spearman: | 0.16 |
r
q
t
s
on-rate (M-1s-1)
on-rate (M-1s-1)
| r-square: | 0.08 |
| --- | --- |
| Pearson: | 0.28 |
| Spearman: | 0.15 |
| r-square: | 0.02 |
| --- | --- |
| Pearson: | 0.13 |
| Spearman: | 0.07 |
| r-square: | 0.07 |
| --- | --- |
| Pearson: | 0.26 |
| Spearman: | 0.14 |
| r-square: | <0.01 |
| --- | --- |
| Pearson: | -0.05 |
| Spearman: | -0.20 |
Trimer
v
x
u
w
| r-square: | 0.08 |
| --- | --- |
| Pearson: | 0.29 |
| Spearman: | 0.25 |
| r-square: | 0.03 |
| --- | --- |
| Pearson: | 0.18 |
| Spearman: | 0.17 |
| r-square: | 0.12 |
| --- | --- |
| Pearson: | 0.35 |
| Spearman: | 0.29 |
| r-square: | 0.13 |
| --- | --- |
| Pearson: | 0.36 |
| Spearman: | 0.30 |
Supplementary Figure 12, On-Rate Correlation to NGS Stats. Scatterplot of relative abundance versus on-rate for RBD a) top representative in given cluster, b) all unique HCDR3, c) all unique concatenated HCDR3+LCDR3, and d) concatenated CDRs. Scatterplot of fold enrichment (10nM to 1nM) versus on-rate for e) top representative in given cluster, f) all unique HCDR3, g) all unique concatenated HCDR3+LCDR3, and h) concatenated CDRs. Scatterplot of relative abundance versus on-rate for S1 i) top representative in given cluster, j) all unique HCDR3, k) all unique concatenated HCDR3+LCDR3, and l) concatenated CDRs. Scatterplot of fold enrichment (10nM to 1nM) versus off-rate for m) top representative in given cluster, n) all unique HCDR3, o) all unique concatenated HCDR3+LCDR3, and p) concatenated CDRs. Scatterplot of relative abundance versus on-rate for trimer q) top representative in given cluster, r) all unique HCDR3, s) all unique concatenated HCDR3+LCDR3, and t) concatenated CDRs. Scatterplot of fold enrichment (10nM to 1nM) versus on-rate for u) top representative in given cluster, v) all unique HCDR3, w) all unique concatenated HCDR3+LCDR3, and x) concatenated CDRs.

## Slide 13
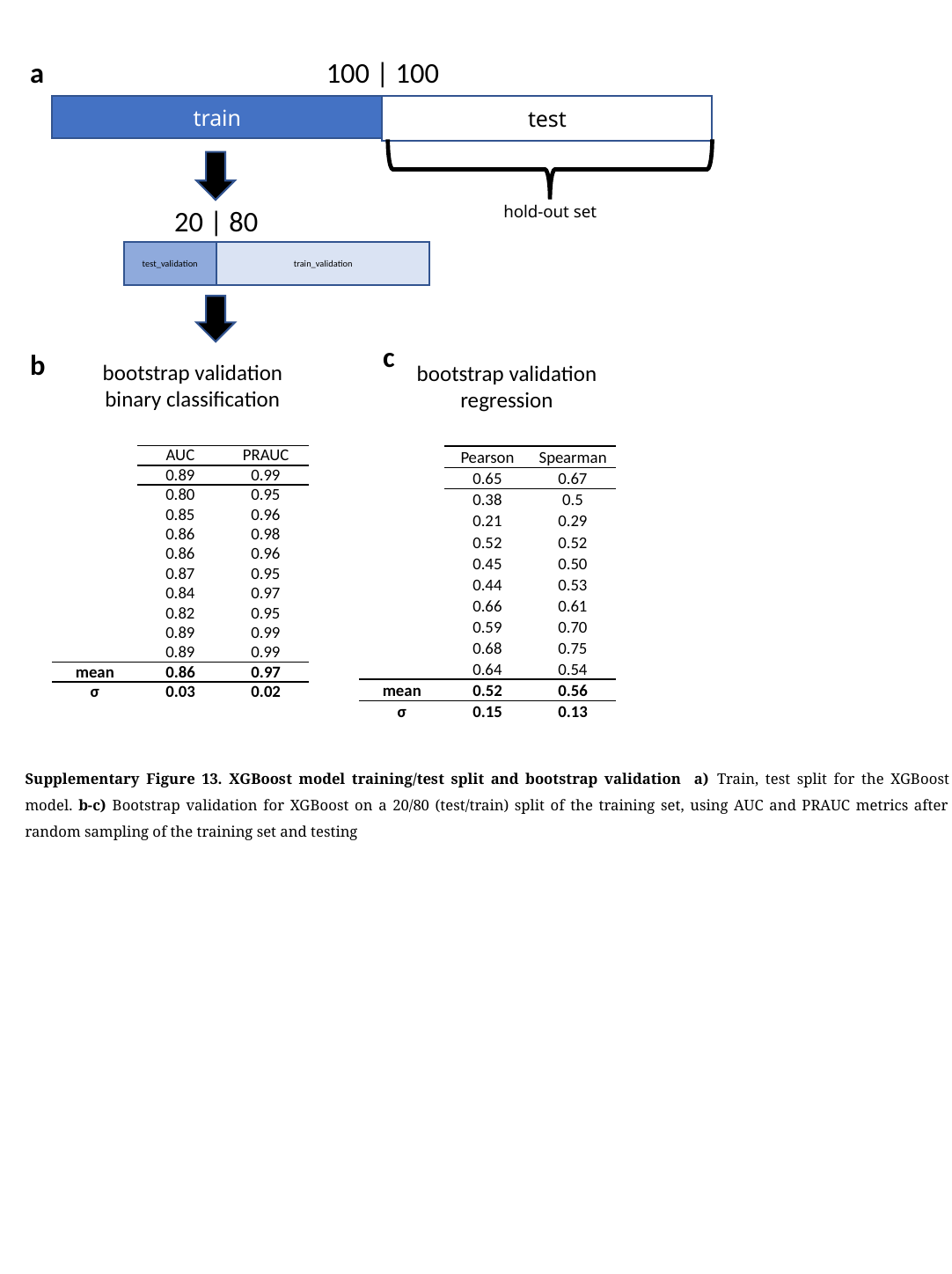

100 | 100
a
test
train
hold-out set
20 | 80
test_validation
train_validation
c
b
bootstrap validation
binary classification
bootstrap validation
regression
| | AUC | PRAUC |
| --- | --- | --- |
| | 0.89 | 0.99 |
| | 0.80 | 0.95 |
| | 0.85 | 0.96 |
| | 0.86 | 0.98 |
| | 0.86 | 0.96 |
| | 0.87 | 0.95 |
| | 0.84 | 0.97 |
| | 0.82 | 0.95 |
| | 0.89 | 0.99 |
| | 0.89 | 0.99 |
| mean | 0.86 | 0.97 |
| σ | 0.03 | 0.02 |
| | Pearson | Spearman |
| --- | --- | --- |
| | 0.65 | 0.67 |
| | 0.38 | 0.5 |
| | 0.21 | 0.29 |
| | 0.52 | 0.52 |
| | 0.45 | 0.50 |
| | 0.44 | 0.53 |
| | 0.66 | 0.61 |
| | 0.59 | 0.70 |
| | 0.68 | 0.75 |
| | 0.64 | 0.54 |
| mean | 0.52 | 0.56 |
| σ | 0.15 | 0.13 |
Supplementary Figure 13. XGBoost model training/test split and bootstrap validation a) Train, test split for the XGBoost model. b-c) Bootstrap validation for XGBoost on a 20/80 (test/train) split of the training set, using AUC and PRAUC metrics after random sampling of the training set and testing

## Slide 14
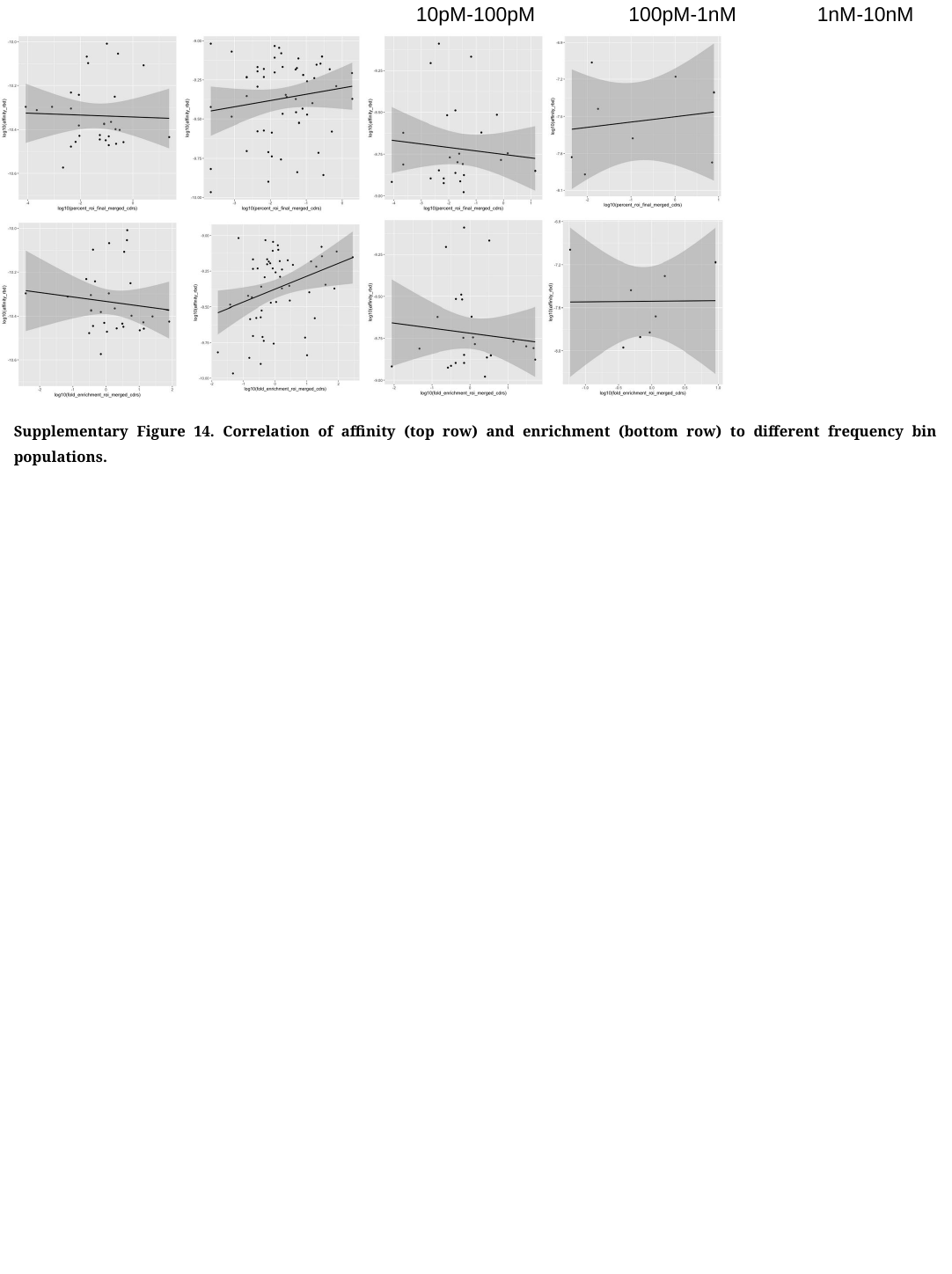

10pM-100pM
100pM-1nM
1nM-10nM
10nM-100nM
Supplementary Figure 14. Correlation of affinity (top row) and enrichment (bottom row) to different frequency bin populations.

## Slide 15
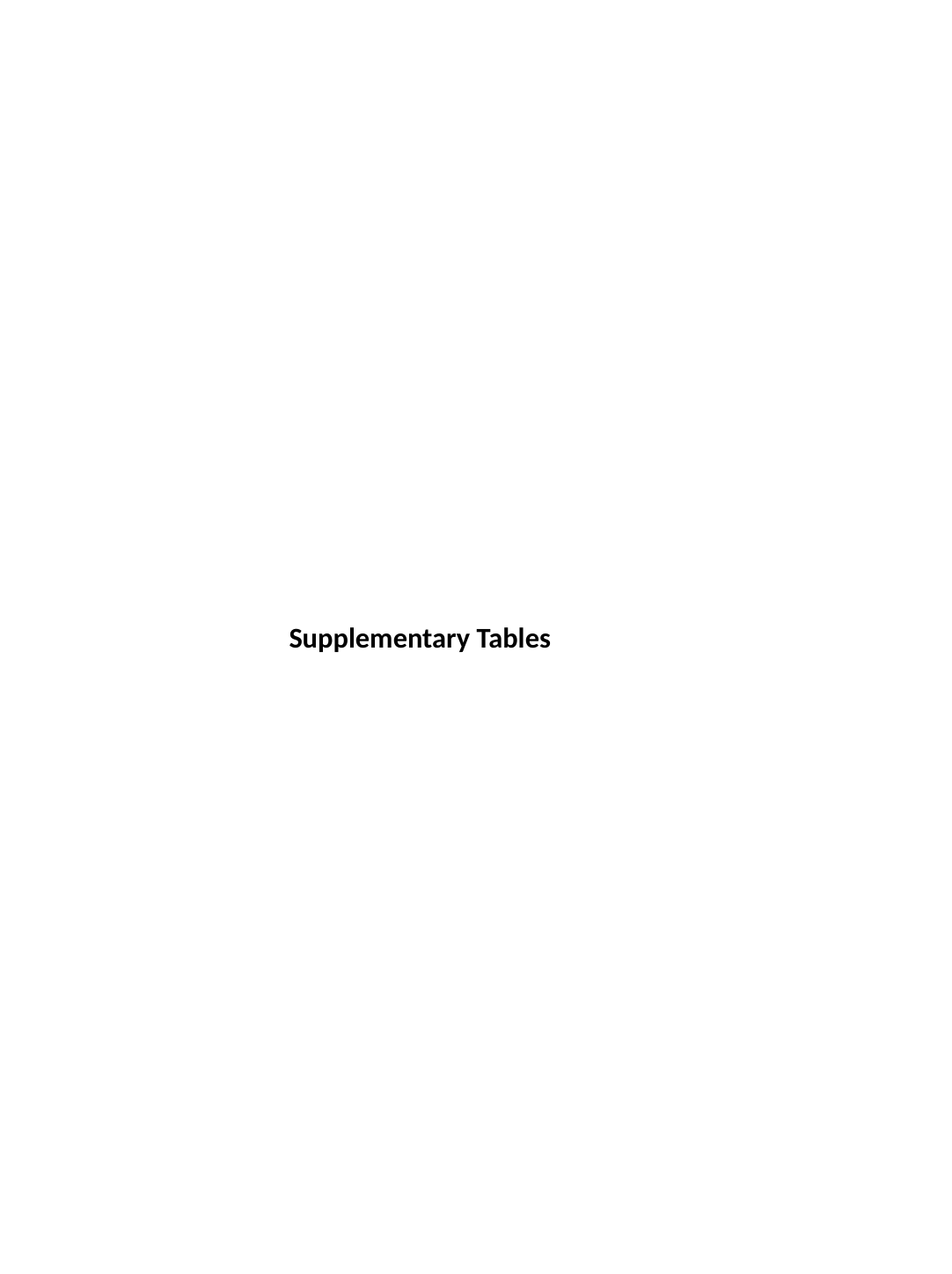

Supplementary Tables

## Slide 16
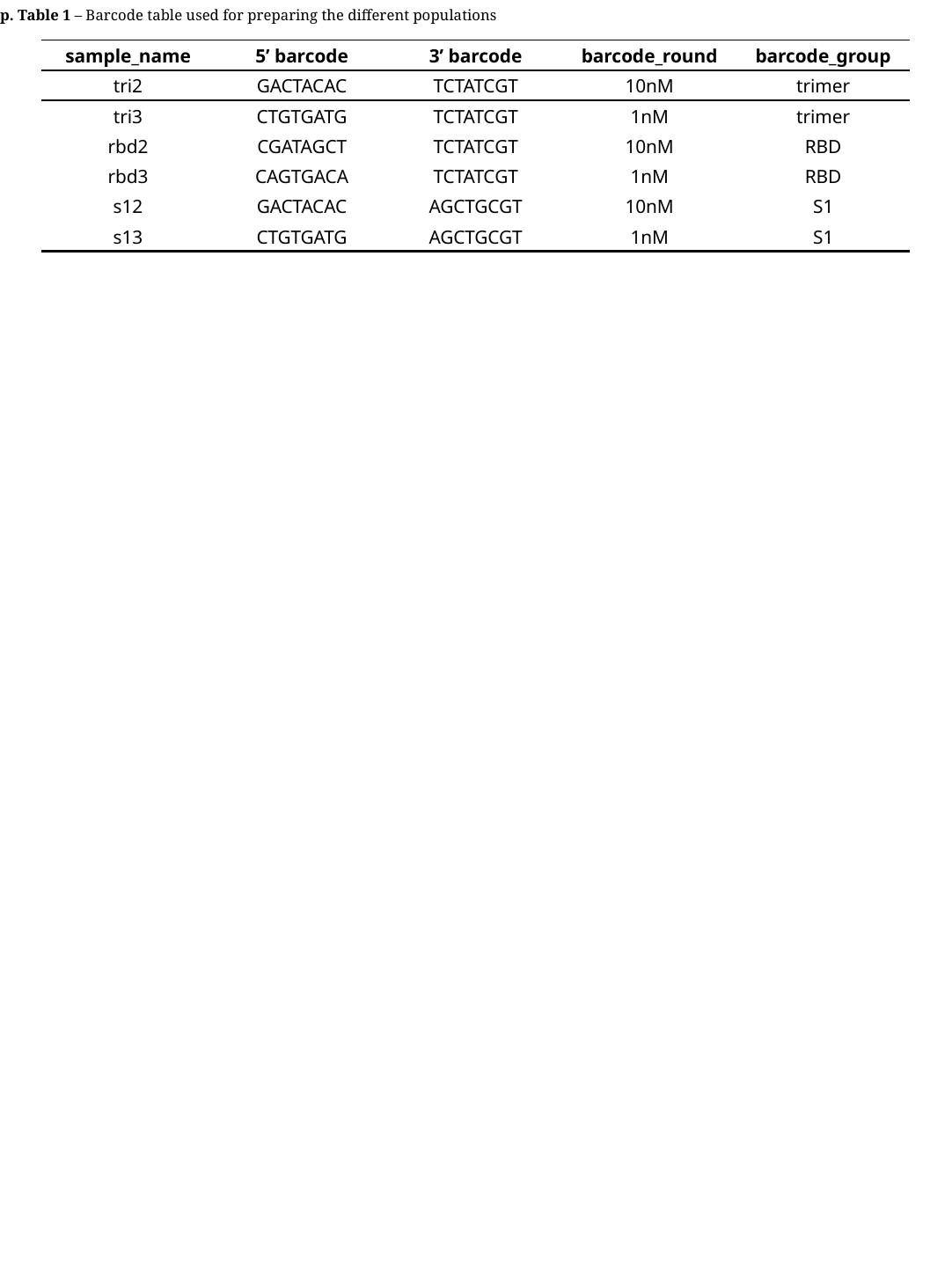

Supp. Table 1 – Barcode table used for preparing the different populations
| sample\_name | 5’ barcode | 3’ barcode | barcode\_round | barcode\_group |
| --- | --- | --- | --- | --- |
| tri2 | GACTACAC | TCTATCGT | 10nM | trimer |
| tri3 | CTGTGATG | TCTATCGT | 1nM | trimer |
| rbd2 | CGATAGCT | TCTATCGT | 10nM | RBD |
| rbd3 | CAGTGACA | TCTATCGT | 1nM | RBD |
| s12 | GACTACAC | AGCTGCGT | 10nM | S1 |
| s13 | CTGTGATG | AGCTGCGT | 1nM | S1 |

## Slide 17
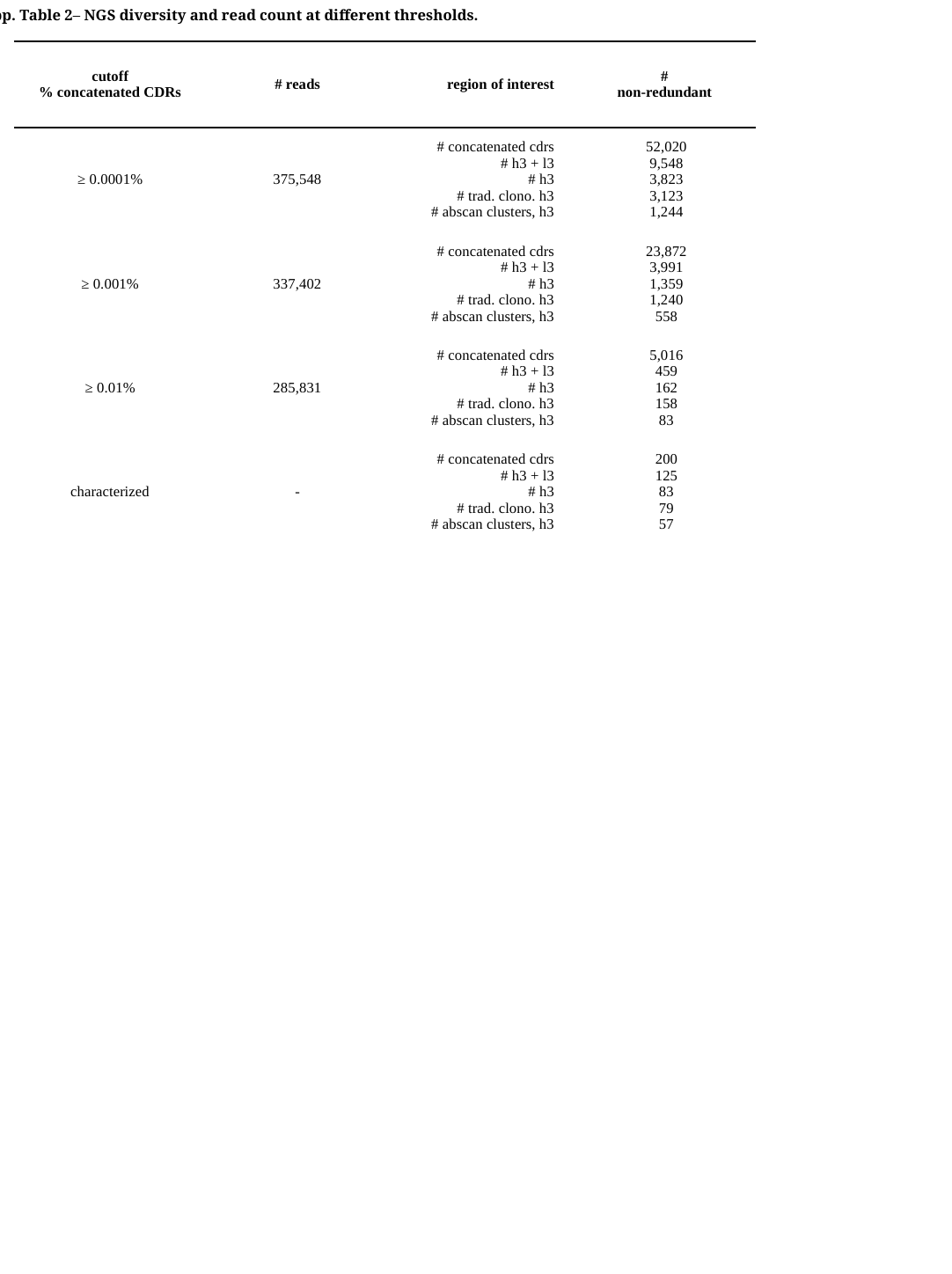

Supp. Table 2– NGS diversity and read count at different thresholds.
| cutoff % concatenated CDRs | # reads | region of interest | # non-redundant |
| --- | --- | --- | --- |
| ≥ 0.0001% | 375,548 | # concatenated cdrs # h3 + l3 # h3 # trad. clono. h3 # abscan clusters, h3 | 52,020 9,548 3,823 3,123 1,244 |
| ≥ 0.001% | 337,402 | # concatenated cdrs # h3 + l3 # h3 # trad. clono. h3 # abscan clusters, h3 | 23,872 3,991 1,359 1,240 558 |
| ≥ 0.01% | 285,831 | # concatenated cdrs # h3 + l3 # h3 # trad. clono. h3 # abscan clusters, h3 | 5,016 459 162 158 83 |
| characterized | - | # concatenated cdrs # h3 + l3 # h3 # trad. clono. h3 # abscan clusters, h3 | 200 125 83 79 57 |

## Slide 18
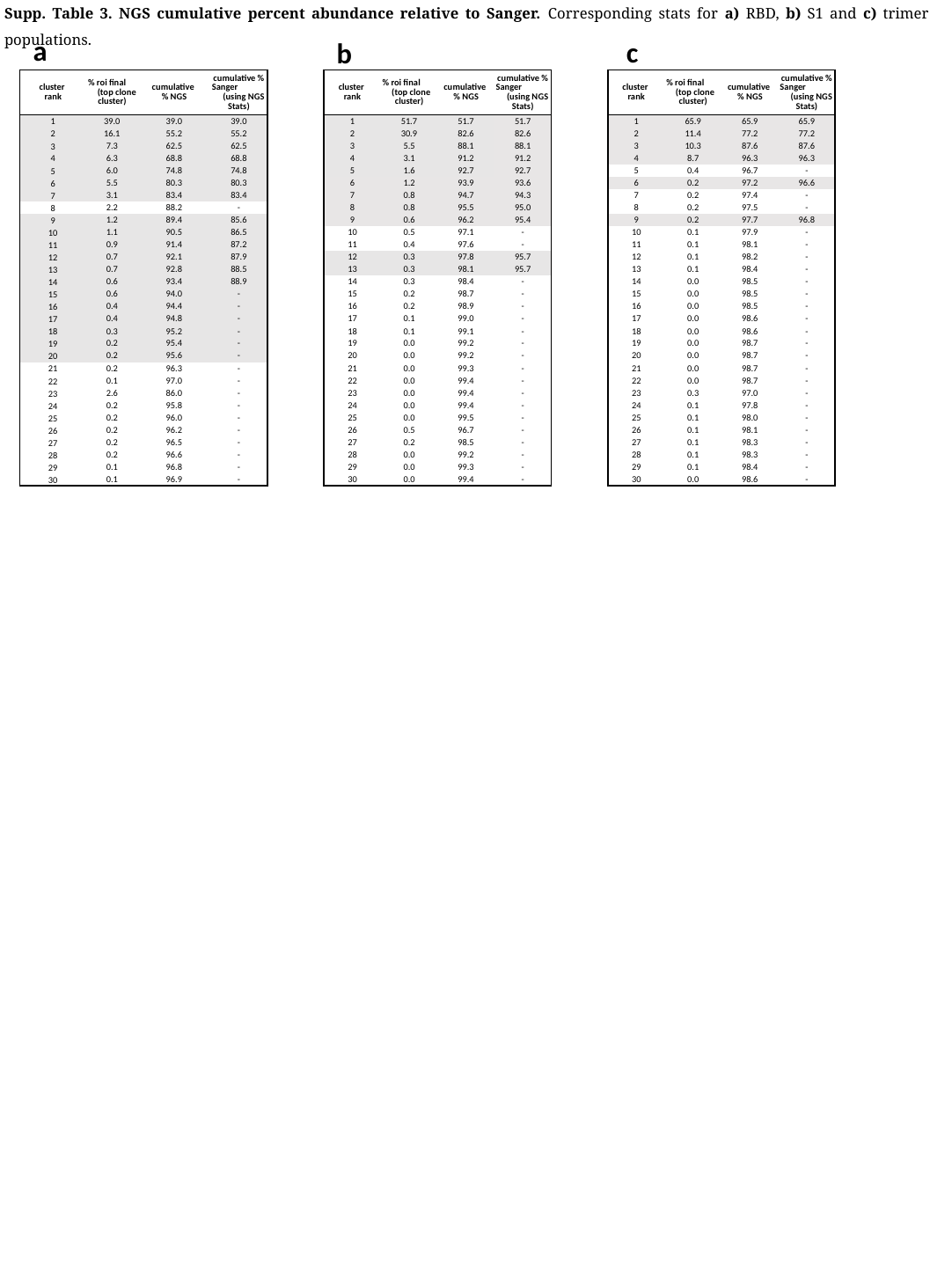

Supp. Table 3. NGS cumulative percent abundance relative to Sanger. Corresponding stats for a) RBD, b) S1 and c) trimer populations.
a
c
b
| cluster rank | % roi final (top clone cluster) | cumulative % NGS | cumulative % Sanger (using NGS Stats) | | cluster rank | % roi final (top clone cluster) | cumulative % NGS | cumulative % Sanger (using NGS Stats) | | cluster rank | % roi final (top clone cluster) | cumulative % NGS | cumulative % Sanger (using NGS Stats) |
| --- | --- | --- | --- | --- | --- | --- | --- | --- | --- | --- | --- | --- | --- |
| 1 | 39.0 | 39.0 | 39.0 | | 1 | 51.7 | 51.7 | 51.7 | | 1 | 65.9 | 65.9 | 65.9 |
| 2 | 16.1 | 55.2 | 55.2 | | 2 | 30.9 | 82.6 | 82.6 | | 2 | 11.4 | 77.2 | 77.2 |
| 3 | 7.3 | 62.5 | 62.5 | | 3 | 5.5 | 88.1 | 88.1 | | 3 | 10.3 | 87.6 | 87.6 |
| 4 | 6.3 | 68.8 | 68.8 | | 4 | 3.1 | 91.2 | 91.2 | | 4 | 8.7 | 96.3 | 96.3 |
| 5 | 6.0 | 74.8 | 74.8 | | 5 | 1.6 | 92.7 | 92.7 | | 5 | 0.4 | 96.7 | - |
| 6 | 5.5 | 80.3 | 80.3 | | 6 | 1.2 | 93.9 | 93.6 | | 6 | 0.2 | 97.2 | 96.6 |
| 7 | 3.1 | 83.4 | 83.4 | | 7 | 0.8 | 94.7 | 94.3 | | 7 | 0.2 | 97.4 | - |
| 8 | 2.2 | 88.2 | - | | 8 | 0.8 | 95.5 | 95.0 | | 8 | 0.2 | 97.5 | - |
| 9 | 1.2 | 89.4 | 85.6 | | 9 | 0.6 | 96.2 | 95.4 | | 9 | 0.2 | 97.7 | 96.8 |
| 10 | 1.1 | 90.5 | 86.5 | | 10 | 0.5 | 97.1 | - | | 10 | 0.1 | 97.9 | - |
| 11 | 0.9 | 91.4 | 87.2 | | 11 | 0.4 | 97.6 | - | | 11 | 0.1 | 98.1 | - |
| 12 | 0.7 | 92.1 | 87.9 | | 12 | 0.3 | 97.8 | 95.7 | | 12 | 0.1 | 98.2 | - |
| 13 | 0.7 | 92.8 | 88.5 | | 13 | 0.3 | 98.1 | 95.7 | | 13 | 0.1 | 98.4 | - |
| 14 | 0.6 | 93.4 | 88.9 | | 14 | 0.3 | 98.4 | - | | 14 | 0.0 | 98.5 | - |
| 15 | 0.6 | 94.0 | - | | 15 | 0.2 | 98.7 | - | | 15 | 0.0 | 98.5 | - |
| 16 | 0.4 | 94.4 | - | | 16 | 0.2 | 98.9 | - | | 16 | 0.0 | 98.5 | - |
| 17 | 0.4 | 94.8 | - | | 17 | 0.1 | 99.0 | - | | 17 | 0.0 | 98.6 | - |
| 18 | 0.3 | 95.2 | - | | 18 | 0.1 | 99.1 | - | | 18 | 0.0 | 98.6 | - |
| 19 | 0.2 | 95.4 | - | | 19 | 0.0 | 99.2 | - | | 19 | 0.0 | 98.7 | - |
| 20 | 0.2 | 95.6 | - | | 20 | 0.0 | 99.2 | - | | 20 | 0.0 | 98.7 | - |
| 21 | 0.2 | 96.3 | - | | 21 | 0.0 | 99.3 | - | | 21 | 0.0 | 98.7 | - |
| 22 | 0.1 | 97.0 | - | | 22 | 0.0 | 99.4 | - | | 22 | 0.0 | 98.7 | - |
| 23 | 2.6 | 86.0 | - | | 23 | 0.0 | 99.4 | - | | 23 | 0.3 | 97.0 | - |
| 24 | 0.2 | 95.8 | - | | 24 | 0.0 | 99.4 | - | | 24 | 0.1 | 97.8 | - |
| 25 | 0.2 | 96.0 | - | | 25 | 0.0 | 99.5 | - | | 25 | 0.1 | 98.0 | - |
| 26 | 0.2 | 96.2 | - | | 26 | 0.5 | 96.7 | - | | 26 | 0.1 | 98.1 | - |
| 27 | 0.2 | 96.5 | - | | 27 | 0.2 | 98.5 | - | | 27 | 0.1 | 98.3 | - |
| 28 | 0.2 | 96.6 | - | | 28 | 0.0 | 99.2 | - | | 28 | 0.1 | 98.3 | - |
| 29 | 0.1 | 96.8 | - | | 29 | 0.0 | 99.3 | - | | 29 | 0.1 | 98.4 | - |
| 30 | 0.1 | 96.9 | - | | 30 | 0.0 | 99.4 | - | | 30 | 0.0 | 98.6 | - |

## Slide 19
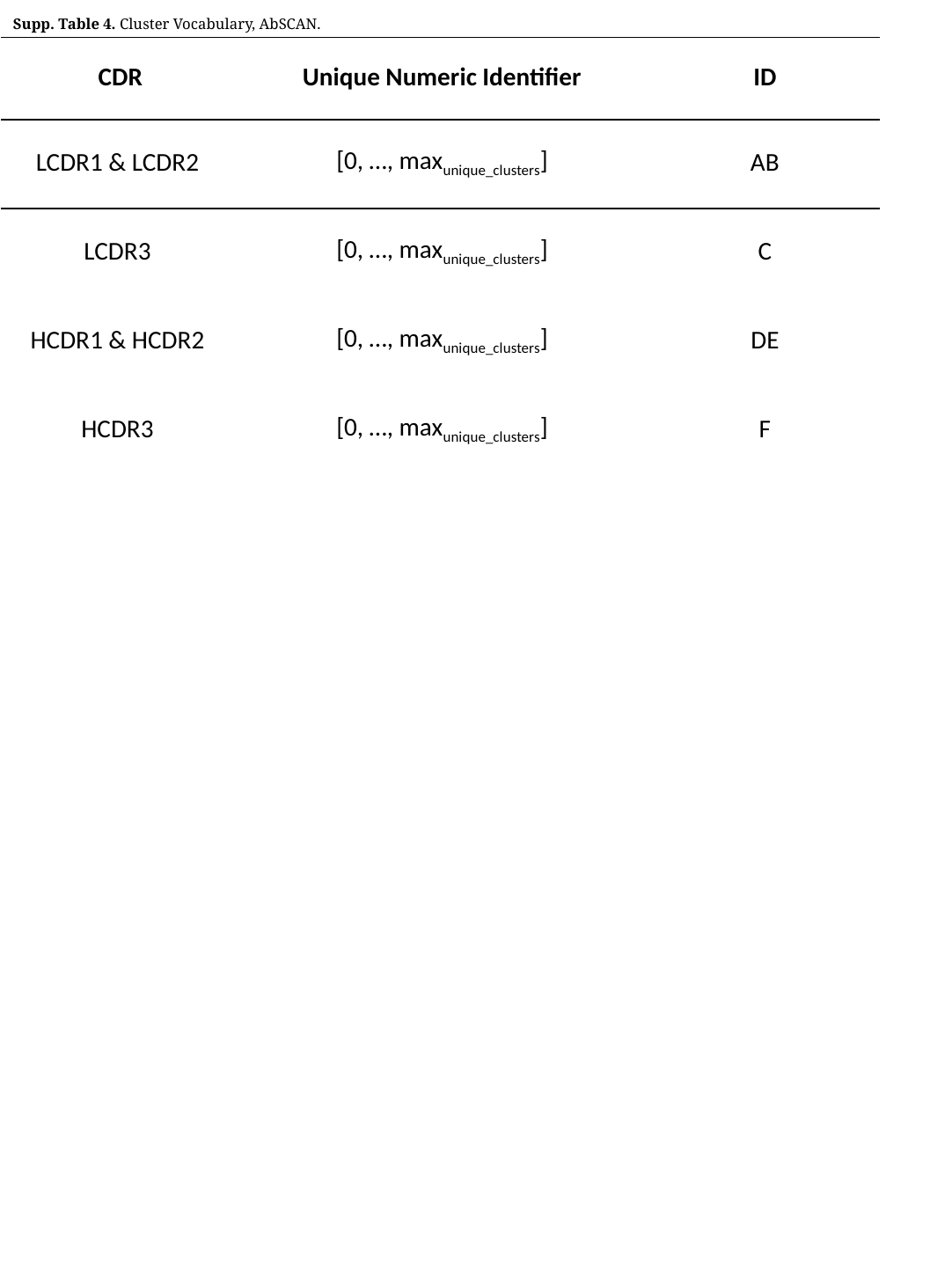

Supp. Table 4. Cluster Vocabulary, AbSCAN.
| CDR | Unique Numeric Identifier | ID |
| --- | --- | --- |
| LCDR1 & LCDR2 | [0, …, maxunique\_clusters] | AB |
| LCDR3 | [0, …, maxunique\_clusters] | C |
| HCDR1 & HCDR2 | [0, …, maxunique\_clusters] | DE |
| HCDR3 | [0, …, maxunique\_clusters] | F |

## Slide 20
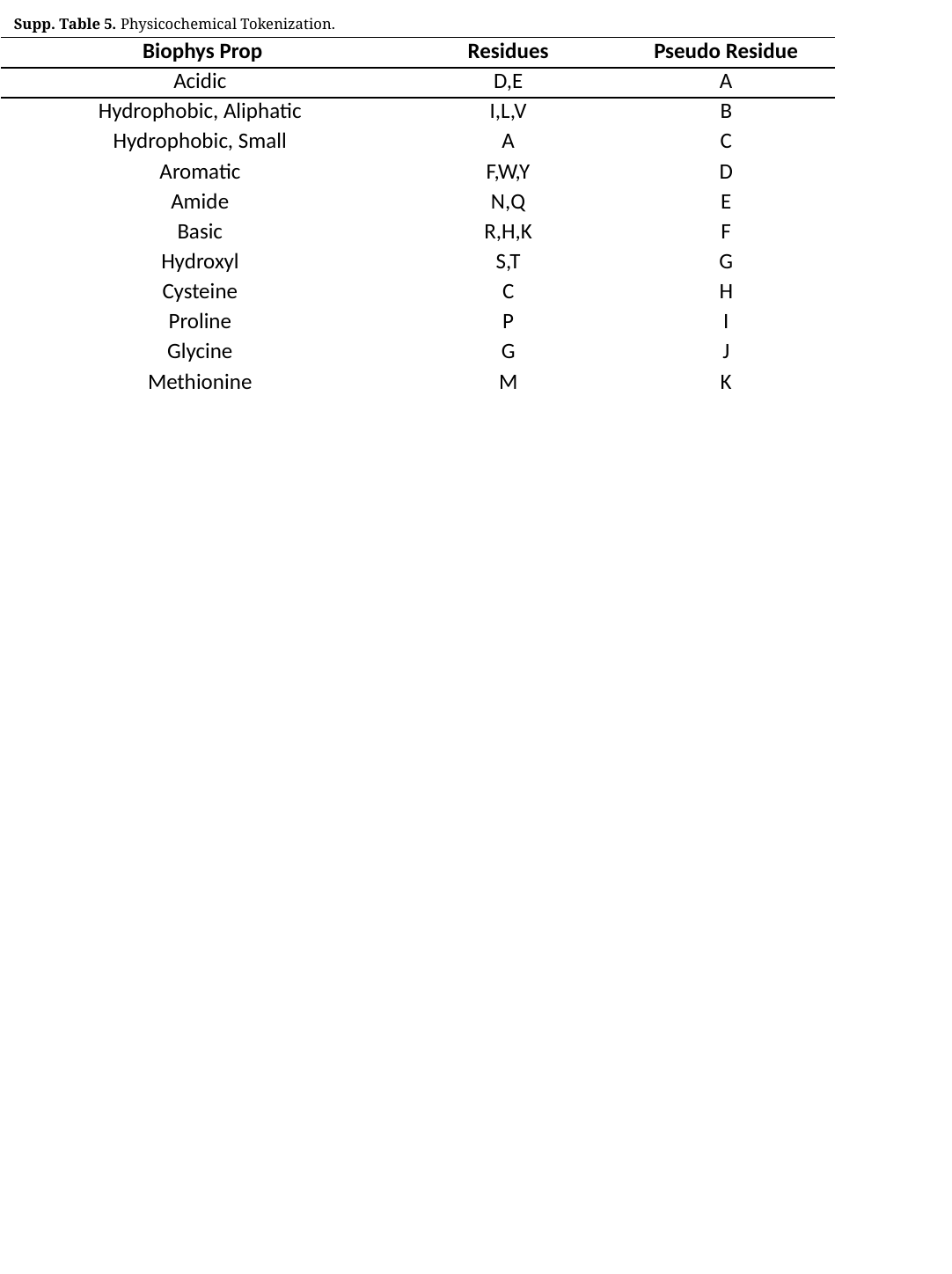

Supp. Table 5. Physicochemical Tokenization.
| Biophys Prop | Residues | Pseudo Residue |
| --- | --- | --- |
| Acidic | D,E | A |
| Hydrophobic, Aliphatic | I,L,V | B |
| Hydrophobic, Small | A | C |
| Aromatic | F,W,Y | D |
| Amide | N,Q | E |
| Basic | R,H,K | F |
| Hydroxyl | S,T | G |
| Cysteine | C | H |
| Proline | P | I |
| Glycine | G | J |
| Methionine | M | K |

## Slide 21
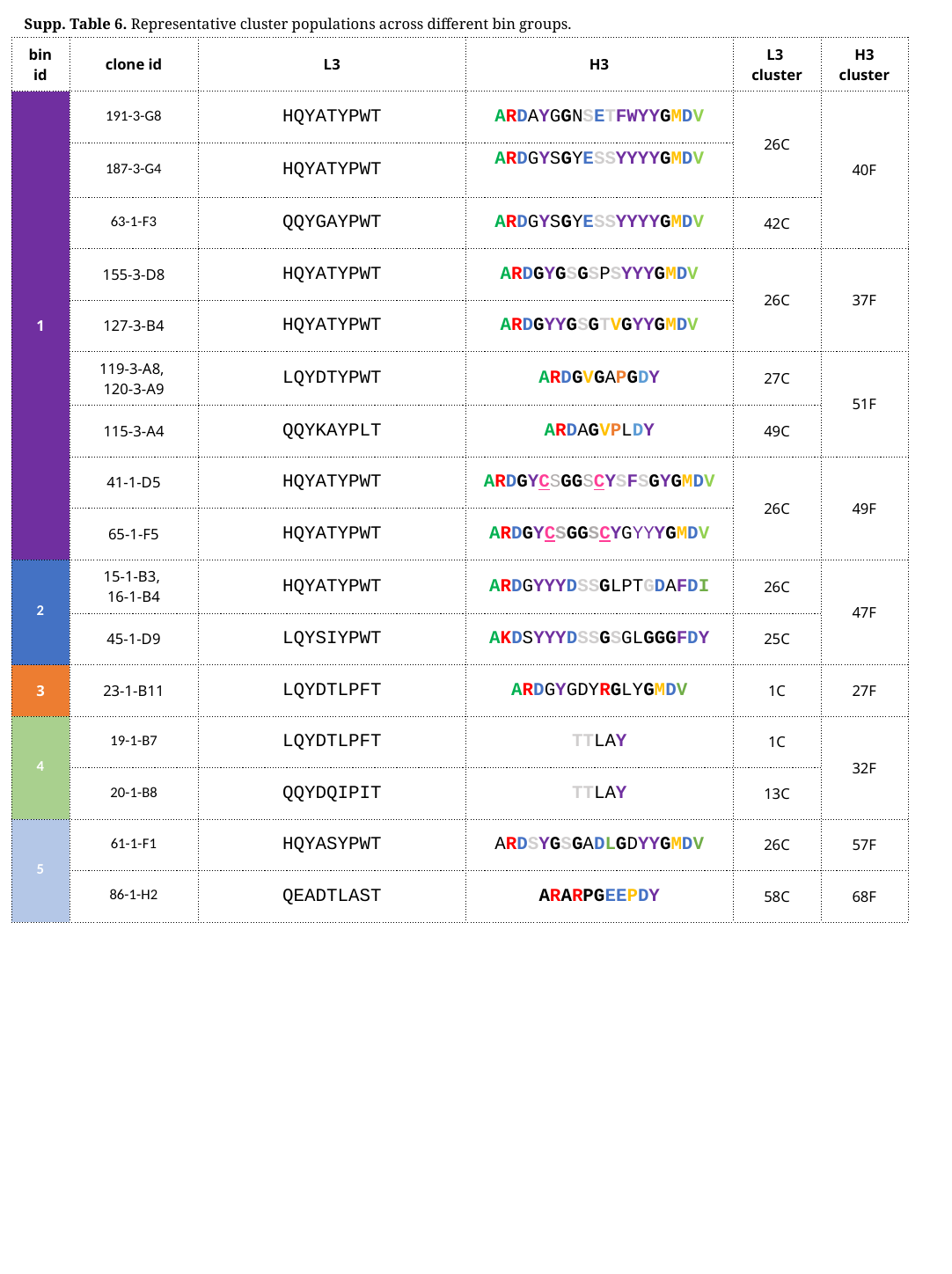

Supp. Table 6. Representative cluster populations across different bin groups.
| bin id | clone id | L3 | H3 | L3 cluster | H3 cluster |
| --- | --- | --- | --- | --- | --- |
| 1 | 191-3-G8 | HQYATYPWT | ARDAYGGNSETFWYYGMDV | 26C | 40F |
| 2 | 187-3-G4 | HQYATYPWT | ARDGYSGYESSYYYYGMDV | 446C | |
| 3 | 63-1-F3 | QQYGAYPWT | ARDGYSGYESSYYYYGMDV | 42C | 40F |
| 1 | 155-3-D8 | HQYATYPWT | ARDGYGSGSPSYYYGMDV | 26C | 37F |
| | 127-3-B4 | HQYATYPWT | ARDGYYGSGTVGYYGMDV | | |
| | 119-3-A8, 120-3-A9 | LQYDTYPWT | ARDGVGAPGDY | 27C | 51F |
| | 115-3-A4 | QQYKAYPLT | ARDAGVPLDY | 49C | |
| | 41-1-D5 | HQYATYPWT | ARDGYCSGGSCYSFSGYGMDV | 26C | 49F |
| | 65-1-F5 | HQYATYPWT | ARDGYCSGGSCYGYYYGMDV | | |
| 2 | 15-1-B3, 16-1-B4 | HQYATYPWT | ARDGYYYDSSGLPTGDAFDI | 26C | 47F |
| 2 | 45-1-D9 | LQYSIYPWT | AKDSYYYDSSGSGLGGGFDY | 25C | |
| 3 | 23-1-B11 | LQYDTLPFT | ARDGYGDYRGLYGMDV | 1C | 27F |
| 4 | 19-1-B7 | LQYDTLPFT | TTLAY | 1C | 32F |
| 5b | 20-1-B8 | QQYDQIPIT | TTLAY | 13C | |
| 5 | 61-1-F1 | HQYASYPWT | ARDSYGSGADLGDYYGMDV | 26C | 57F |
| 5b | 86-1-H2 | QEADTLAST | ARARPGEEPDY | 58C | 68F |

## Slide 22
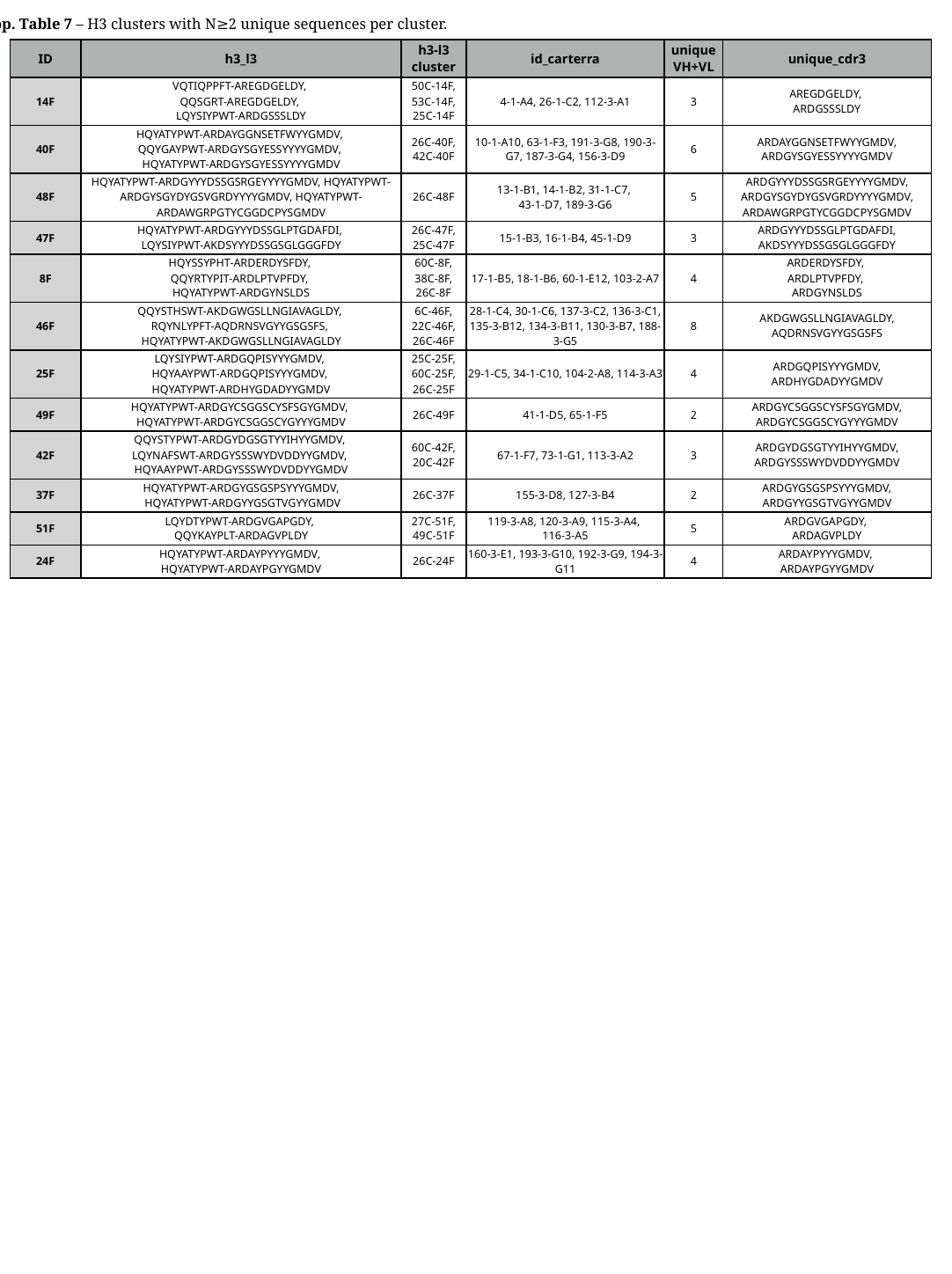

Supp. Table 7 – H3 clusters with N≥2 unique sequences per cluster.
| ID | h3\_l3 | h3-l3 cluster | id\_carterra | unique VH+VL | unique\_cdr3 |
| --- | --- | --- | --- | --- | --- |
| 14F | VQTIQPPFT-AREGDGELDY, QQSGRT-AREGDGELDY, LQYSIYPWT-ARDGSSSLDY | 50C-14F, 53C-14F, 25C-14F | 4-1-A4, 26-1-C2, 112-3-A1 | 3 | AREGDGELDY, ARDGSSSLDY |
| 40F | HQYATYPWT-ARDAYGGNSETFWYYGMDV, QQYGAYPWT-ARDGYSGYESSYYYYGMDV, HQYATYPWT-ARDGYSGYESSYYYYGMDV | 26C-40F, 42C-40F | 10-1-A10, 63-1-F3, 191-3-G8, 190-3-G7, 187-3-G4, 156-3-D9 | 6 | ARDAYGGNSETFWYYGMDV, ARDGYSGYESSYYYYGMDV |
| 48F | HQYATYPWT-ARDGYYYDSSGSRGEYYYYGMDV, HQYATYPWT-ARDGYSGYDYGSVGRDYYYYGMDV, HQYATYPWT-ARDAWGRPGTYCGGDCPYSGMDV | 26C-48F | 13-1-B1, 14-1-B2, 31-1-C7, 43-1-D7, 189-3-G6 | 5 | ARDGYYYDSSGSRGEYYYYGMDV, ARDGYSGYDYGSVGRDYYYYGMDV, ARDAWGRPGTYCGGDCPYSGMDV |
| 47F | HQYATYPWT-ARDGYYYDSSGLPTGDAFDI, LQYSIYPWT-AKDSYYYDSSGSGLGGGFDY | 26C-47F, 25C-47F | 15-1-B3, 16-1-B4, 45-1-D9 | 3 | ARDGYYYDSSGLPTGDAFDI, AKDSYYYDSSGSGLGGGFDY |
| 8F | HQYSSYPHT-ARDERDYSFDY, QQYRTYPIT-ARDLPTVPFDY, HQYATYPWT-ARDGYNSLDS | 60C-8F, 38C-8F, 26C-8F | 17-1-B5, 18-1-B6, 60-1-E12, 103-2-A7 | 4 | ARDERDYSFDY, ARDLPTVPFDY, ARDGYNSLDS |
| 46F | QQYSTHSWT-AKDGWGSLLNGIAVAGLDY, RQYNLYPFT-AQDRNSVGYYGSGSFS, HQYATYPWT-AKDGWGSLLNGIAVAGLDY | 6C-46F, 22C-46F, 26C-46F | 28-1-C4, 30-1-C6, 137-3-C2, 136-3-C1, 135-3-B12, 134-3-B11, 130-3-B7, 188-3-G5 | 8 | AKDGWGSLLNGIAVAGLDY, AQDRNSVGYYGSGSFS |
| 25F | LQYSIYPWT-ARDGQPISYYYGMDV, HQYAAYPWT-ARDGQPISYYYGMDV, HQYATYPWT-ARDHYGDADYYGMDV | 25C-25F, 60C-25F, 26C-25F | 29-1-C5, 34-1-C10, 104-2-A8, 114-3-A3 | 4 | ARDGQPISYYYGMDV, ARDHYGDADYYGMDV |
| 49F | HQYATYPWT-ARDGYCSGGSCYSFSGYGMDV, HQYATYPWT-ARDGYCSGGSCYGYYYGMDV | 26C-49F | 41-1-D5, 65-1-F5 | 2 | ARDGYCSGGSCYSFSGYGMDV, ARDGYCSGGSCYGYYYGMDV |
| 42F | QQYSTYPWT-ARDGYDGSGTYYIHYYGMDV, LQYNAFSWT-ARDGYSSSWYDVDDYYGMDV, HQYAAYPWT-ARDGYSSSWYDVDDYYGMDV | 60C-42F, 20C-42F | 67-1-F7, 73-1-G1, 113-3-A2 | 3 | ARDGYDGSGTYYIHYYGMDV, ARDGYSSSWYDVDDYYGMDV |
| 37F | HQYATYPWT-ARDGYGSGSPSYYYGMDV, HQYATYPWT-ARDGYYGSGTVGYYGMDV | 26C-37F | 155-3-D8, 127-3-B4 | 2 | ARDGYGSGSPSYYYGMDV, ARDGYYGSGTVGYYGMDV |
| 51F | LQYDTYPWT-ARDGVGAPGDY, QQYKAYPLT-ARDAGVPLDY | 27C-51F, 49C-51F | 119-3-A8, 120-3-A9, 115-3-A4, 116-3-A5 | 5 | ARDGVGAPGDY, ARDAGVPLDY |
| 24F | HQYATYPWT-ARDAYPYYYGMDV, HQYATYPWT-ARDAYPGYYGMDV | 26C-24F | 160-3-E1, 193-3-G10, 192-3-G9, 194-3-G11 | 4 | ARDAYPYYYGMDV, ARDAYPGYYGMDV |

## Slide 23
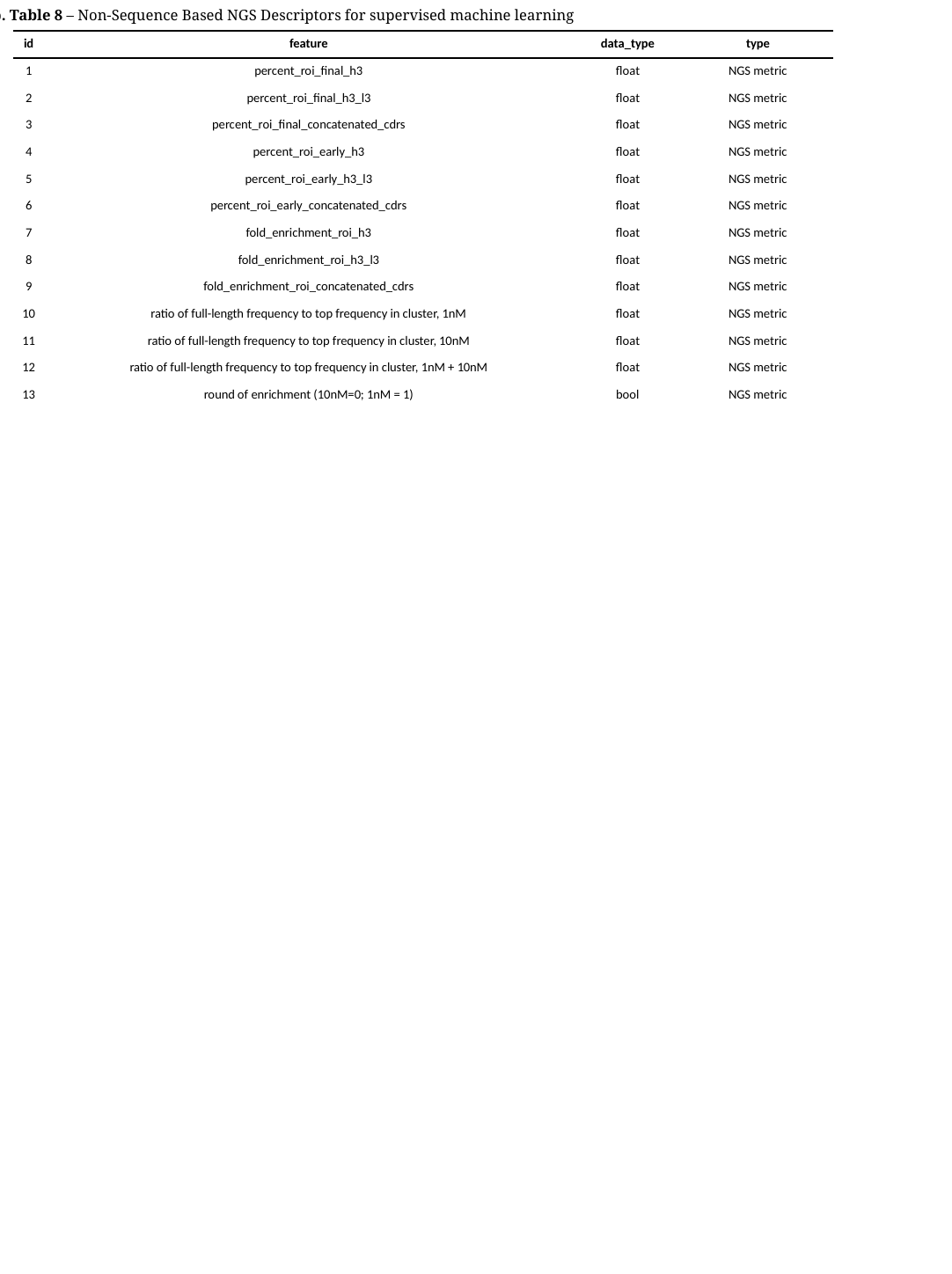

Supp. Table 8 – Non-Sequence Based NGS Descriptors for supervised machine learning
| id | feature | data\_type | type |
| --- | --- | --- | --- |
| 1 | percent\_roi\_final\_h3 | float | NGS metric |
| 2 | percent\_roi\_final\_h3\_l3 | float | NGS metric |
| 3 | percent\_roi\_final\_concatenated\_cdrs | float | NGS metric |
| 4 | percent\_roi\_early\_h3 | float | NGS metric |
| 5 | percent\_roi\_early\_h3\_l3 | float | NGS metric |
| 6 | percent\_roi\_early\_concatenated\_cdrs | float | NGS metric |
| 7 | fold\_enrichment\_roi\_h3 | float | NGS metric |
| 8 | fold\_enrichment\_roi\_h3\_l3 | float | NGS metric |
| 9 | fold\_enrichment\_roi\_concatenated\_cdrs | float | NGS metric |
| 10 | ratio of full-length frequency to top frequency in cluster, 1nM | float | NGS metric |
| 11 | ratio of full-length frequency to top frequency in cluster, 10nM | float | NGS metric |
| 12 | ratio of full-length frequency to top frequency in cluster, 1nM + 10nM | float | NGS metric |
| 13 | round of enrichment (10nM=0; 1nM = 1) | bool | NGS metric |

## Slide 24
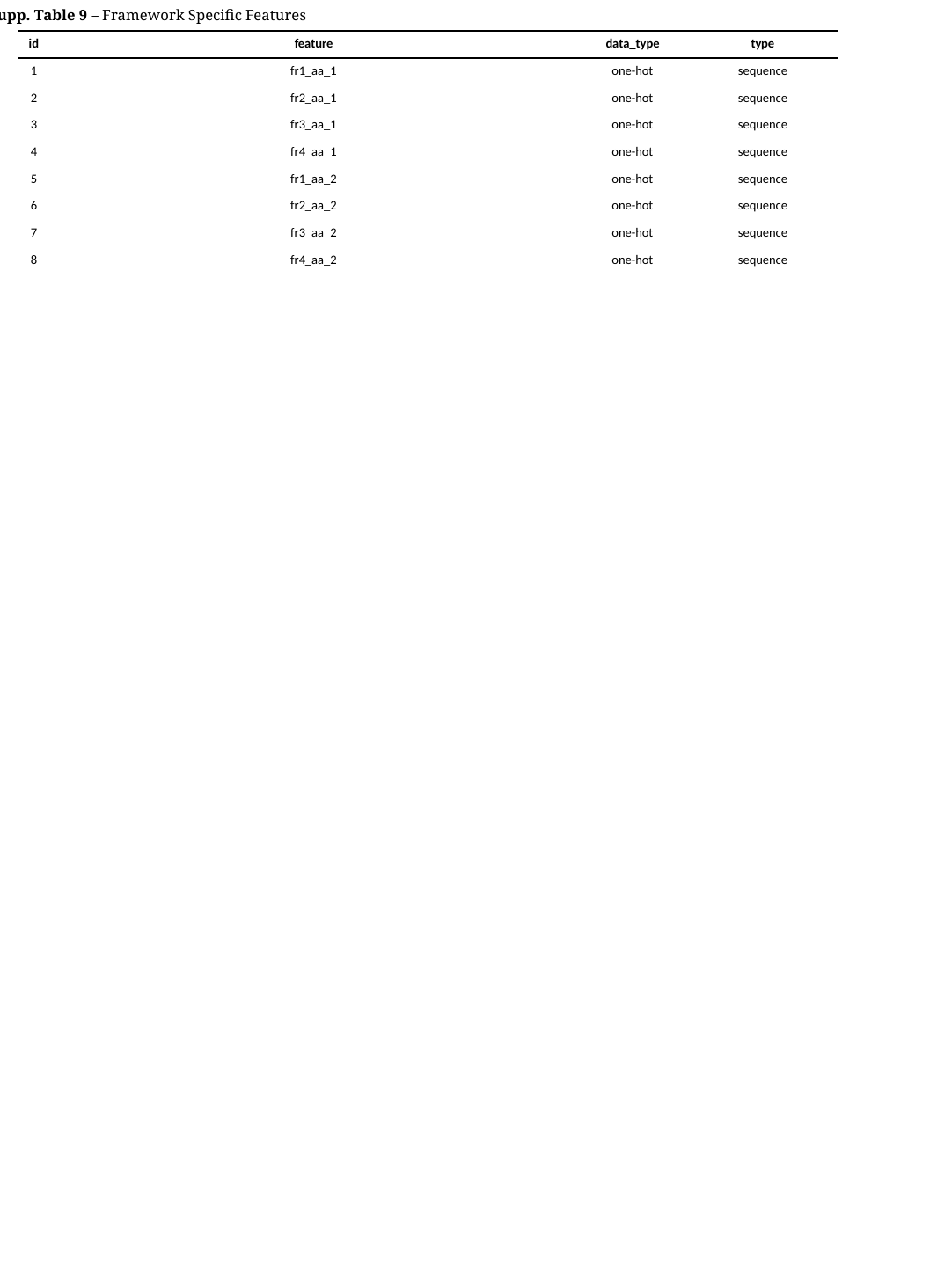

Supp. Table 9 – Framework Specific Features
| id | feature | data\_type | type |
| --- | --- | --- | --- |
| 1 | fr1\_aa\_1 | one-hot | sequence |
| 2 | fr2\_aa\_1 | one-hot | sequence |
| 3 | fr3\_aa\_1 | one-hot | sequence |
| 4 | fr4\_aa\_1 | one-hot | sequence |
| 5 | fr1\_aa\_2 | one-hot | sequence |
| 6 | fr2\_aa\_2 | one-hot | sequence |
| 7 | fr3\_aa\_2 | one-hot | sequence |
| 8 | fr4\_aa\_2 | one-hot | sequence |

## Slide 25
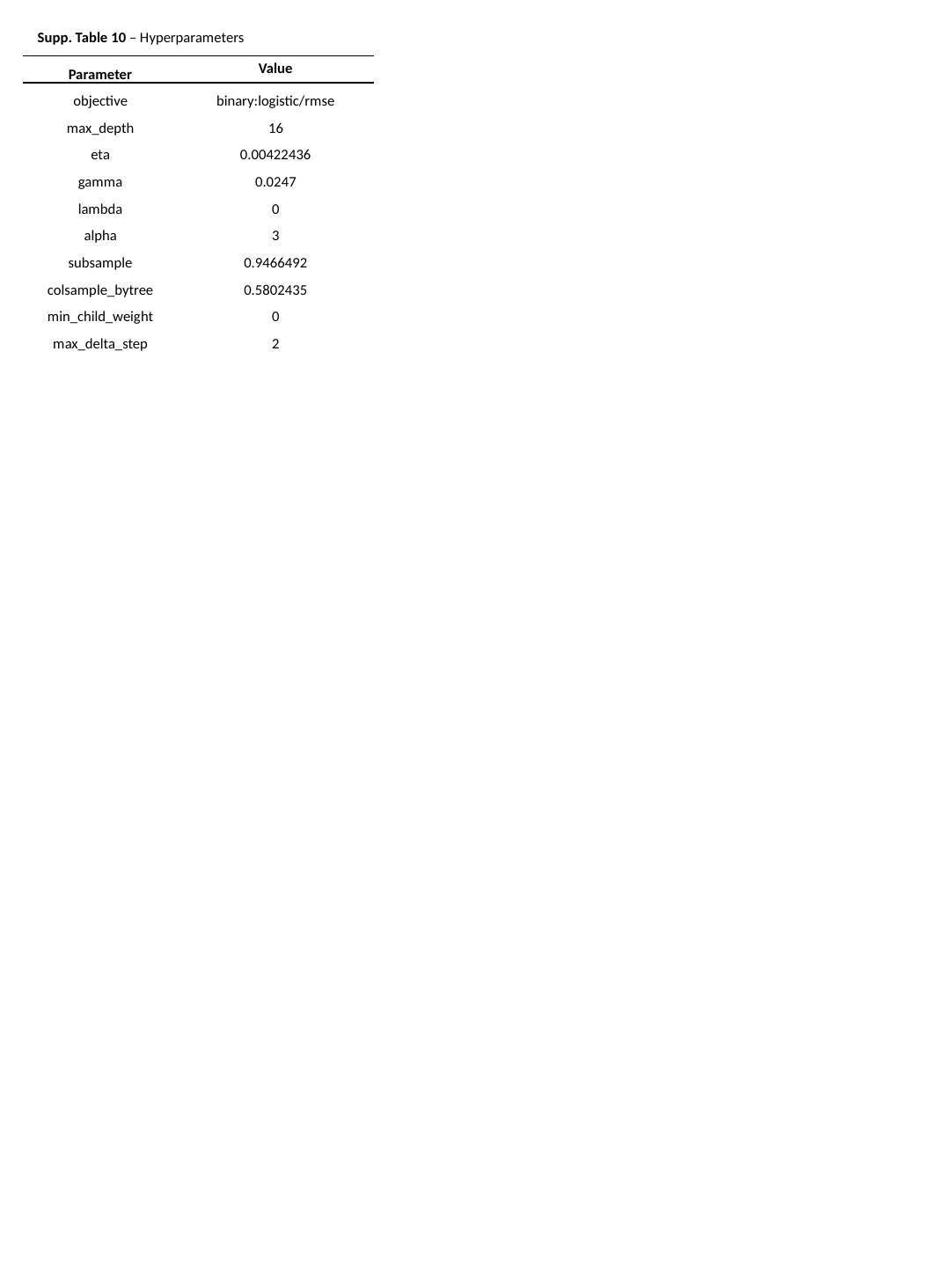

Supp. Table 10 – Hyperparameters
| Parameter | Value |
| --- | --- |
| objective | binary:logistic/rmse |
| max\_depth | 16 |
| eta | 0.00422436 |
| gamma | 0.0247 |
| lambda | 0 |
| alpha | 3 |
| subsample | 0.9466492 |
| colsample\_bytree | 0.5802435 |
| min\_child\_weight | 0 |
| max\_delta\_step | 2 |
